# The Ink4a/Arf locus operates as a regulator of the circadian clock modulating RAS activity

**DOI:** 10.1371/journal.pbio.2002940

**Published:** 2017-12-07

**Authors:** Rukeia El-Athman, Nikolai N. Genov, Jeannine Mazuch, Kaiyang Zhang, Yong Yu, Luise Fuhr, Mónica Abreu, Yin Li, Thomas Wallach, Achim Kramer, Clemens A. Schmitt, Angela Relógio

**Affiliations:** 1 Charité - Universitätsmedizin Berlin, Corporate Member of Freie Universität Berlin, Humboldt - Universität zu Berlin, and Berlin Institute of Health, Institute for Theoretical Biology, Germany; 2 Charité - Universitätsmedizin Berlin, Corporate Member of Freie Universität Berlin, Humboldt - Universität zu Berlin, and Berlin Institute of Health, Medical Department of Hematology, Oncology, and Tumor Immunology, and Molecular Cancer Research Center, Germany; 3 Max-Delbrück-Center for Molecular Medicine in the Helmholtz Association, Berlin, Germany; 4 Charité - Universitätsmedizin Berlin, Corporate Member of Freie Universität Berlin, Humboldt - Universität zu Berlin, and Berlin Institute of Health, Laboratory of Chronobiology, Berlin, Germany; Washington University in St. Louis, United States of America

## Abstract

The mammalian circadian clock and the cell cycle are two major biological oscillators whose coupling influences cell fate decisions. In the present study, we use a model-driven experimental approach to investigate the interplay between clock and cell cycle components and the dysregulatory effects of RAS on this coupled system. In particular, we focus on the *Ink4a/Arf* locus as one of the bridging clock-cell cycle elements. Upon perturbations by the rat sarcoma viral oncogene (RAS), differential effects on the circadian phenotype were observed in wild-type and *Ink4a/Arf* knock-out mouse embryonic fibroblasts (MEFs), which could be reproduced by our modelling simulations and correlated with opposing cell cycle fate decisions. Interestingly, the observed changes can be attributed to in silico phase shifts in the expression of core-clock elements. A genome-wide analysis revealed a set of differentially expressed genes that form an intricate network with the circadian system with enriched pathways involved in opposing cell cycle phenotypes. In addition, a machine learning approach complemented by cell cycle analysis classified the observed cell cycle fate decisions as dependent on *Ink4a/Arf* and the oncogene RAS and highlighted a putative fine-tuning role of *Bmal1* as an elicitor of such processes, ultimately resulting in increased cell proliferation in the *Ink4a/Arf* knock-out scenario. This indicates that the dysregulation of the core-clock might work as an enhancer of RAS-mediated regulation of the cell cycle. Our combined in silico and in vitro approach highlights the important role of the circadian clock as an *Ink4a/Arf*-dependent modulator of oncogene-induced cell fate decisions, reinforcing its function as a tumour-suppressor and the close interplay between the clock and the cell cycle network.

## Author summary

In mammals, the circadian clock controls the punctual regulation of biological processes, which, in turn, affect physiology and behaviour, allowing for the synchronisation of internal time to environmental light-dark cycles. Malfunctions of the circadian clock are associated with pathological phenotypes including cancer. Given the range of molecular time-dependent processes, including metabolism, DNA repair, and the cell cycle, the clock is hypothesised to act as a tumour suppressor. With the help of mathematical modelling and whole-genome analysis combined with machine learning, we investigated the RAS-dependent dysregulation of the circadian clock. We find that the tumour-suppressor *Ink4a/Arf* acts as a key mediator of RAS oncogene-induced changes in the circadian system, thereby mediating the interplay between the clock and the cell cycle.

## Introduction

Earth’s rotation within repetitive cycles of 24 hours (h) led to the evolution of an endogenous timing system across all phyla: the circadian clock, which allows organisms to anticipate and adapt to environmental changes such as light and darkness. At the molecular level, the generation of circadian rhythms in each cell is based on a complex interplay of positive and negative transcriptional/translational feedback loops. In mammals, two main feedback loops have been dissected in greater detail [1]. In the period (PER)/cryptochrome (CRY) loop, a heterodimer of the proteins circadian locomotor output cycles kaput (CLOCK) and brain and muscle aryl hydrocarbon receptor nuclear translocator-like protein (BMAL) drives the expression of genes from the *Per* and *Cry* families. The resulting proteins translocate back to the nucleus, form complexes, and bind to the CLOCK/BMAL heterodimer, thereby inhibiting their own synthesis. CLOCK/BMAL also induces the transcription of the nuclear receptor gene families reverse strand of ERBA (*Rev-Erb*) and RAR-related orphan receptors (*Ror*) within the ROR/*Bmal*/REV-ERB loop. REV-ERB and ROR compete for the binding to ROR response elements (ROREs) in the promoter region of *Bmal1*, regulating its transcription in antagonistic ways. By controlling rhythmic RNA and protein abundance, the cellular endogenous clock regulates circadian rhythms of a variety of biological processes such as rest/activity cycles, metabolism, hormone level, immune functions, and the metabolism of drugs [2,3]. Disturbances of the circadian system are associated with many pathological phenotypes including cancer [4,5], though the effect of the circadian clock in tumourigenesis is still an issue of ongoing debate. Several epidemiological studies reported increased occurrence of cancer in long-term shift workers [6,7], indicating that disrupted circadian rhythms may constitute a cancer risk factor. However, a causative connection is still disputed [8]. Particularly at the molecular level, there is a need for further investigation regarding the putative role of the circadian clock in tumourigenesis. An oncogenic-driven mouse model identified CLOCK and BMAL1 as playing a key role in regulating proliferation and differentiation pointing to a complex role of the circadian clock in cancer progression [9]. Perturbations of the circadian clock (via experimental chronic jet lag) in mice led to accelerated tumour growth, which could be counterbalanced by regular timing of food access [10]. While the disruption of core-clock genes such as *Per1* and *Per2* has also been associated with cancer promoting mechanisms, [11,12], their role in tumourigenesis is still debatable because a recent study showed that *Per1* or *Per2* deficiency does not lead to more tumour-prone phenotypes in mice [13]. In turn, cancer strongly influences circadian rhythms of biological processes, such as fatty acid and cholesterol biosynthesis, and metabolic oscillations [14,15]. Current attempts of chronobiological cancer treatment provide promising results, leading to a reduced toxic effect of drugs and increasing survival times of cancer patients [16,17].

One of the processes via which the circadian clock could potentially influence tumourigenesis, namely by triggering malignant proliferation, might be the cell-division cycle in whose regulation the clock is involved [18]. Several links between the circadian clock and the cell cycle have been reported for mammalian cells [19–22]. The clock was found to unidirectionally gate the cell cycle in mouse liver cells via a circadian expression of the cell cycle regulator Wee1 [19], whereas in mouse fibroblasts, the circadian clock was reported to be phase-shifted by mitosis, possibly via concentration changes of the PER-CRY complexes [20]. More recently, the non-POU domain containing octamer binding (NONO)-PER protein complex has been reported to couple the clock and the cell cycle by activating the rhythmic transcription of the cell cycle checkpoint gene *Ink4a* [22], which encodes, as part of the *Cdkn2a* locus, the cyclin-dependent kinase (CDK) inhibitor p16^Ink4a^ [23]. p16^Ink4a^ can be activated by the oncogene RAS, leading to cell cycle arrest in the Gap 1 (G1) phase, a tumour-suppressive mechanism counteracting abnormal cell proliferation [24]. A correlation between the expression level of *Per2* and the expression of *Ink4a* mRNA has also been reported [25], indicating that PER is a positive regulator of *Ink4a* expression and might be responsible for its circadian expression. In addition, *Cdkn2a* encodes for another tumour suppressor and cell cycle regulator protein, alternate open reading frame (ARF) [26], which is mainly activated by mitogenic stimulation through the cell cycle checkpoint protein avian myelocytomatosis viral oncogene homolog (MYC) [27], but can also be induced via oncogenic RAS [28]. ARF interacts in a circadian time (CT)-dependent manner with a mouse double minute 2 homolog (MDM2), a negative regulator of the tumour suppressor gene p53 [29]. p53, in turn, directly modulates the expression of *Per2* by binding to a response element in its promoter region, which is overlapping with the E-box *cis*-element essential for the CLOCK/BMAL binding and the transcriptional activation of *Per2* [30]. In addition, the transcription factor E2F was described as a putative bridging element between the circadian clock and the cell cycle [31].

Recently, we demonstrated that RAS induces a dysregulation of the mammalian circadian clock in HaCaT keratinocyte cells [32]. We showed that the induction of RAS leads to a lengthening of the circadian period while inhibition of the RAS/mitogen-activated protein kinases (MAPK) pathway causes a period shortening. These findings were supported by using a mathematical model to analyse the rhythmic properties of the core-clock network in silico [1,32]. Other mathematical models have been developed that investigate the cell cycle-related functions of the tumour suppressor genes *p53* and *Arf*, as well as *Mdm2* [33–35]. Despite the accumulating experimental and in silico evidence pointing to a role of the circadian clock as a suppressor of aberrant cell proliferation, the underlying molecular mechanisms are not yet fully understood. Although mathematical models exist that couple elements of the cell cycle to the components of the circadian clock [36–42], none of them include the cell cycle regulatory genes *Ink4a* and *Arf*, nor do they include specific parameters for the investigation of the role of oncogenic-mediated signalling via RAS.

Using mouse embryonic fibroblasts (MEFs) as a model system, we investigated the *Ink4a/Arf*- and *Bmal1*-dependent influence of RAS on the circadian phenotype. To attain a deeper understanding concerning the molecular interactions of the circadian clock with components of the cell cycle, we constructed a single-cell semi-quantitative mathematical model. The model allows for the coupling of components of the cell division cycle with the core-clock network, enabling the interpretation of the RAS-mediated effect on the circadian clock phenotype observed in *Ink4a/Arf* knockout MEFs as compared to their wild-type (WT) counterparts. In particular, the model was used to attain a better understanding of the mechanism by which INK4a and ARF might influence properties of the circadian clock. Furthermore, we performed a comprehensive bioinformatics analysis to investigate the systemic effects of different experimental perturbations at the cellular level. Expression data of a set of core-clock genes, as well as in silico simulations, show that RAS overexpression influences the transcriptional expression and period of core-clock genes such as *Bmal* and *Per* differentially in the Ink4a/Arf^+/+^ and Ink4a/Arf^-/-^ scenarios, which points to a regulatory role of *Ink4a/Arf* in the RAS-mediated effect on the circadian clock. By analysing the relevance of selected cell cycle components in mediating the RAS-induced change on the circadian period via *Ink4a/Arf* in silico, we show that the presence of the transcription factors E2F1 and p53 is necessary for simulating this phenotype. Genome-wide transcriptional profiling data from Ink4a/Arf^-/-^ MEFs and their corresponding WT MEFs (Ink4a/Arf^+/+^) are in line with the mathematical predictions showing that *Ink4a/Arf* play an essential role in the RAS-mediated effect on the gene expression levels of core-clock and cell cycle-related genes. Hence, our findings resulting from a combined computational and experimental approach provide a deeper insight into the dynamics of the cross-talk between oncogene-induced perturbations of the circadian system and the cell cycle and reveal a novel role for *Ink4a/Arf* as an important regulator of the RAS-mediated effect on the circadian clock phenotype.

## Results

### *Ink4a/Arf*^*+/+*^ and *Ink4a/Arf*^*-/-*^ MEFs show different circadian phenotypes upon oncogenic perturbation

**Fig 1:**
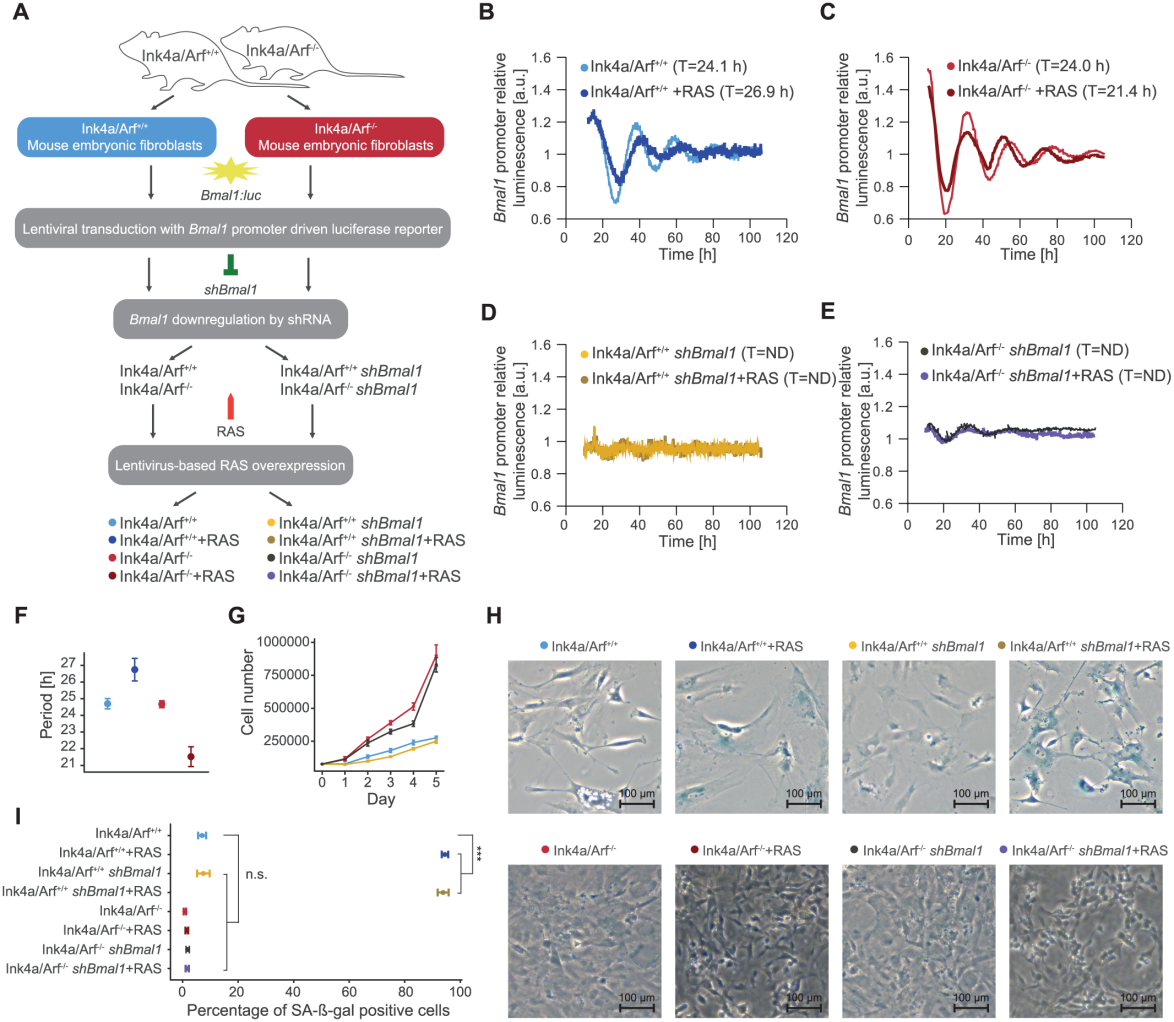
Ink4a/Arf^+/+^ and Ink4a/Arf^-/-^ MEFs show different circadian phenotypes upon RAS overexpression. (A) Schematic representation of the experimental setup to investigate the influence of RAS on the circadian phenotype via *Ink4a/Arf*. MEFs from *Ink4a/Arf* WT (Ink4a/Arf^+/+^) and their *Ink4a/Arf* knock-out littermates (Ink4a/Arf^-/-^) were lentivirally transduced with a *Bmal1*-promoter driven luciferase construct (*Bmal1*:*Luc*). To investigate the effect of the core-clock in this cell model system, *Bmal1* was downregulated by shRNA. The effect of the oncogene RAS was examined by overexpression of RAS. Bioluminescence was measured over five days. Shown are representative data for eight different conditions, as indicated. (B) RAS overexpression leads to an increase of the period in Ink4a/Arf^+/+^ MEFs (26.9 h, dark blue) compared to the corresponding control (24.1 h, light blue). (C) RAS overexpression shortens the period of Ink4a/Arf^-/-^ MEFs (24.0 h, dark red) compared to the corresponding control (21.4 h, light red). RAS overexpression disrupts the circadian clock in *Bmal1* knock-down conditions in (D) Ink4a/Arf^+/+^ MEFs and (E) Ink4a/Arf^-/-^ MEFs. (F) Summary of circadian period phenotype measurements for Ink4a/Arf^+/+^ MEFs and Ink4a/Arf^-/-^ MEFs with and without RAS overexpression (*n* = 5; mean and SEM). (G) The cell number of Ink4a/Arf^+/+^ MEFs and Ink4a/Arf^-/-^ MEFs with and without shBmal1 was monitored over five days (*n* = 3; mean and SEM). Ink4a/Arf^-/-^ MEFs proliferate faster than their corresponding Ink4a/Arf^+/+^ littermates, independent from *Bmal1* knockdown. (H) The cell cycle arrest phenotypes were estimated by SA-ß-Gal staining in Ink4a/Arf^+/+^ MEFs and their Ink4a/Arf^-/-^ littermates with or without RAS overexpression and *Bmal1* downregulation. (I) Percentage of SA-ß-Gal staining positive cells (*n* = 3; mean and SEM). RAS overexpression significantly increased the number of senescent cells in the Ink4a/Arf^+/+^ MEFs compared to the WT. There is no effect of *Bmal1* downregulation in the Ink4a/Arf^-/-^ cell population. Statistical significance was determined by *t* test with *p*-values corrected for multiple testing with the Benjamini and Hochberg method. ***p < 0.001. Numerical values are provided in S1 Data. MEF, mouse embryonic fibroblasts; ND, not defined; n.s., not significant; RAS, rat sarcoma viral oncogene; shRNA, short hairpin RNA; T, period; WT, wild-type.

Recently, we showed that oncogenic RAS influences properties of the circadian clock by lengthening the circadian period in different cell types [32]. To attain a deeper mechanistic insight on how RAS perturbs the circadian clock and on its subsequent effect on the cell cycle and cell proliferation, we used a well-established cellular model system of MEFs. We compared the circadian phenotypes of MEFs from WT mice (Ink4a/Arf^+/+^) to their littermate knock-out MEFs (Ink4a/Arf^-/-^), which carry a targeted deletion of exons 2 and 3 of the tumour suppressor gene *Cdkn2a*, disrupting both *Ink4a* and *Arf* [23]. We analysed the effect of RAS on the circadian clock phenotype by bioluminescence recordings and the induction of senescent cell cycle arrest by SA-ß-Gal staining. MEFs were lentivirally transduced with a *Bmal1*-promoter driven luciferase construct (*Bmal1*:*Luc*) and bioluminescence was recorded for 5 days as schematically represented in Fig 1A. The bioluminescence data shows similar clock phenotypes for Ink4a/Arf^-/-^ MEFs and their corresponding Ink4a/Arf^+/+^ littermates with average period values of around 24 h (T = 24.7 ± 0.3 h for Ink4a/Arf^+/+^ MEFs and T = 24.2 ± 0.2 h for Ink4a/Arf^-/-^ MEFs, *n* = 5; mean and SEM; representative results in Fig 1B and 1C, summary of the data in Table 1), indicating that the period of the circadian clock is not influenced by the knock-out of *Ink4a/Arf*. The viability of the cells was not affected by the knockout (S1A Fig).

**Table 1:** The RAS-mediated effect on the circadian period is dependent on *Ink4a/Arf*.

	Mean Values Unmodified RAS Period ± SEM (h)	Mean Values RAS Overexpression Period ± SEM (h)
**Ink4a/Arf^+/+^**	24.7 ± 0.3	26.7 ± 0.7
**Ink4a/Arf^-/-^**	24.2 ± 0.2	21.7 ± 0.6
**Ink4a/Arf^+/+^*shBmal1***	ND	ND
**Ink4a/Arf^-/-^*shBmal1***	ND	ND

The circadian period was estimated by bioluminescence recordings of a *Bmal1*:*Luc* reporter construct in Ink4a/Arf^+/+^ and Ink4a/Arf^-/-^ MEFs. Average values for the period ± SEM are given (*n* = 5 WT mice and their corresponding Ink4a/Arf^-/-^ littermates, with 2 additional independent transductions per condition). RAS overexpression induces a lengthening of the period in Ink4a/Arf^+/+^ MEFs (*p* < 0.005) and a shortening in Ink4a/Arf^-/-^ MEFs (*p* < 0.0005). MEF, mouse embryonic fibroblasts; ND, not defined; RAS, rat sarcoma viral oncogene; WT, wild-type.

To specifically study the interaction of the RAS/MAPK pathway with components of the circadian clock, oncogenic RAS (H-RAS G12V) was overexpressed in both cell types. Interestingly, upon RAS overexpression, we observed a change of the circadian clock phenotype: the RAS overexpressing Ink4a/Arf^+/+^ cells (T = 26.7 ± 0.7 h, *n* = 5; mean and SEM) show a 2 h longer period as compared to the WT condition (T = 24.7 ± 0.3 h, *n* = 5; mean and SEM; *p* = 0.004, Student *t* test), whereas in the RAS-infected Ink4a/Arf^-/-^ MEFs, a decrease of 2.5 h in the period was observed (T = 21.7 ± 0.6 h, *n* = 5; mean and SEM; *p* = 0.0003, Student *t* test) as compared to cells with unmodified RAS levels (T = 24.2 ± 0.2 h, *n* = 5; mean and SEM; representative results in Fig 1B and 1C, summary of the data in Table 1). To investigate a putative effect of RAS on core-clock components, *Bmal1* was downregulated by short hairpin RNA (shRNA) prior to the stable expression of oncogenic RAS in both Ink4a/Arf^-/-^ MEFs and their WT littermates. As expected, the downregulation of *Bmal1* disrupted circadian rhythmicity (representative results in Fig 1D and 1E, summary of the data in Table 1). Bioluminescence data from several independent experiments (five WT mice and Ink4a/Arf^-/-^ littermates, with two independent transductions per condition) are summarised in Fig 1F and Table 1 and reproduced our representative results shown in Fig 1B and 1C. Ink4a/Arf^-/-^ MEFs proliferate faster than the Ink4a/Arf^+/+^ MEFs, independent of the downregulation of *Bmal1* (Fig 1G).

To investigate the effect of RAS overexpression and *Bmal1* downregulation on cellular senescence, we performed SA-ß-Gal staining using Ink4a/Arf^-/-^ MEFS and WT littermates (*n* = 3 WT mice and Ink4a/Arf^-/-^ littermates) and tested for senescence-associated galactosidase activity. While the Ink4a/Arf^+/+^ MEF population has a low amount of senescence cells (7 ± 1.44%, *n* = 3; mean and SEM), RAS overexpression leads to a strong increase (94.5 ± 1.15%, *n* = 3; mean and SEM; Fig 1H and 1I). The results are in agreement with published data showing that RAS overexpression increases the percentage of senescent cells in WT MEFs [24]. In comparison, the population of Ink4a/Arf^-/-^ MEFs shows the lowest number of senescent cells (0.83 ± 0.44%, *n* = 3; mean and SEM) independent of RAS overexpression (1.5 ± 0.5%, *n* = 3; mean and SEM; Fig 1H and 1I). The downregulation of *Bmal1* shows no significant effect on the senescence phenotype (Fig 1H and 1I).

### A mathematical model for the coupling of the circadian oscillator with the cell division cycle

To explore the observed *Ink4a/Arf*-dependent effect of RAS overexpression on the clock—an increased circadian period in WT MEFs but a decreased period in Ink4a/Arf^-/-^ cells (Fig 1B–1E)—we developed a novel semi-quantitative mathematical model coupling the mammalian cell cycle and the circadian clock using ordinary differential equations (ODEs).

**Fig 2:**
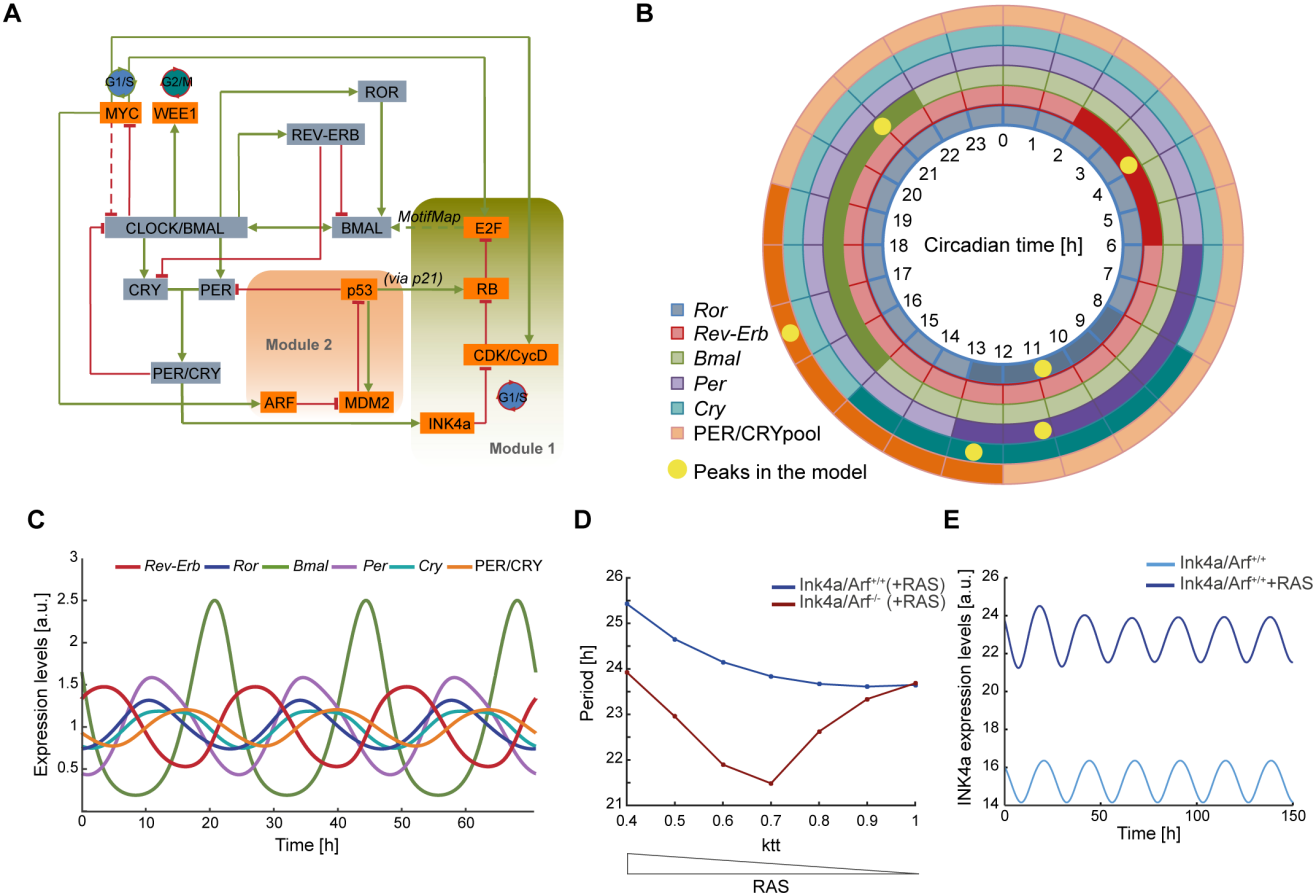
Circadian properties of the mathematical model reproduce the RAS-mediated effect on the clock. (A) Simplified network representation of the mathematical model. The model consists of seven core-clock components (grey boxes) and nine cell cycle components (orange boxes). Activating (green lines) and inhibiting (red lines) interactions known from literature and a potential transcriptional activation (reported in MotifMap) are represented. The dashed red line from MYC to CLOCK/BMAL represents the overall interference with BMAL-mediated transcription by competitive E-box binding. Module 1 consists of the INK4a/RB1/E2F1 pathway (green) and module 2 of the ARF/MDM2/p53 pathway (orange). (B) In silico peak phases of clock genes from the model are consistent with experimental data. The highest mRNA expression intervals (experimental published data) from the core-clock genes *Ror* (blue), *Rev-Erb* (red), *Bmal* (green), *Per* (purple), *Cry* (turquoise) and the cytoplasmic and nuclear PER/CRY protein complexes (orange) are depicted in darker colours within the circles. Yellow dots represent the peak of expression as simulated by the model. (C) Shown are in silico expression profiles for the five core-clock genes *Bmal*, *Per*, *Cry*, *Ror*, and *Rev-Erb* and for the PER/CRY protein complex. The period was set to 23.65 h and the phase of *Bmal* to CT21. (D) The model reproduces experimental clock phenotypes qualitatively. In silico expression data show that upon simulation of RAS overexpression, the Ink4a/Arf^+/+^ system acquires a longer period and Ink4a/Arf^-/-^ system a shorter period compared to the corresponding simulated WT system. RAS overexpression was simulated by decreasing the parameter *ktt* to 0.4 (*ktt* = 1 WT, *ktt* < 1 increase of RAS). (E) RAS overexpression increases *Ink4a* expression levels in silico. WT, wild-type.

The model contains all elements of our previously published single-cell model of the mammalian circadian core-clock [1], the cell cycle elements INK4a and ARF, and a minimal selection of their respective interaction partners, which allow for the connection to the core-clock. These include the cell cycle checkpoint regulators *Myc* (G1/S) and *Wee1* (G2/M) and components of two INK4a- and ARF-dependent signalling pathways: the INK4a/ retinoblastoma-associated protein 1 (RB1)/E2F1 pathway (module 1) and the ARF/MDM2/p53 pathway (module 2). The resulting regulatory network includes nine additional elements (MYC, WEE1, ARF, MDM2, INK4, CDK/Cyc, p53, RB1, E2F1) and their corresponding transcriptional and translational components (Fig 2A, S2 Fig). In total, the model comprises 46 variables and 170 parameters.

In the model, the CLOCK/BMAL complex activates the transcription of *Wee1* and represses *Myc* transcription as reported in the literature [19,43,44]. The transcriptional activation of *Ink4a* by the PER-NONO complex in a circadian manner [45,46] is modelled as a positive interaction between the PER/CRY complex and *Ink4a* transcription. By forming a complex with D-type CDKs CDK4 and CDK6, INK4a prevents the interaction with the cell cycle checkpoint regulator Cyclin D (CycD), thereby inhibiting the subsequent phosphorylation of the RB1, a key regulator of the E2F family of transcription factors (E2F1, E2F2, and E2F3a) [47–51]. The dephosphorylated active form of RB1 inhibits the dissociation of the RB1/E2F complex leading to the inactivation of E2F-mediated transcription of cell cycle genes. The cell cycle/core-clock loop in module 1 is completed by a predicted transcriptional activation of *Bmal* by E2F because E2F potentially binds to the promoter region of *Bmal1* (as reported by MotifMap, the genome-wide map of candidate regulatory motif sites for humans [52]). The prediction of E2F as an important binding element between the clock and the cell cycle is further supported by data from the unicellular red alga *Cyanidioschyzon merolae* in which time-dependent phosphorylation of E2F promotes the G1/S transition and a mutation of the E2F phosphorylation sites results in an uncoupling of cell cycle progression from the circadian clock [31]. Altogether, these results point to a putative connection between E2F and the clock which—given the existing data—can be assumed to happen via *Bmal1*. Module 2 contains the ARF/MDM2/p53 pathway and represents an indirect feedback from ARF to the core-clock. ARF is encoded by the same gene locus as INK4a and can be activated by oncogenic MYC or oncogenic RAS [47]. Accumulated ARF associates with the p53 inhibitor MDM2 and leads to its rapid proteasomal degradation. This decreases MDM2-mediated ubiquitination of the tumour suppressor p53 and promotes its stabilisation, which in turn activates the transcription of *Mdm2* [53,54]. A recent study describes a p53 response element located in the promoter region of *Per2*, which overlaps with the E-box cis-elements crucial for CLOCK/BMAL-mediated *Per2* transcription [30]. Thus, the binding of p53 strongly represses the transcription of *Per2* by competing with the CLOCK/BMAL binding to its promoter [30]. Apart from the strong negative influence of p53 on *Per2*, PER2 is also known to transcriptionally modulate p53 [55], and this regulation is thought to be positive [39]. For simplicity, we have merged the mutual influence into one negative interaction from p53 to *Per2*. In addition, p53 inhibits the phosphorylation of RB1 via the p21/CDK/CycE/RB1 pathway [56,57]. Although we cannot exclude the possibility that other elements may also be involved in connecting the clock and the cell cycle elements INK4a and ARF, the chosen modules represent a minimal functional set well-supported by published data [44,58–60] that enables us to investigate the properties of the system in silico. The model development and the complete network scheme is described in greater detail in the supplementary information (S1 Text, S2 Fig). The resulting model is robust to perturbations in the range of ± 10% as shown by the control coefficient analysis over all parameters (S1 Text). The period of the model system was adjusted to 23.65 h and the phase of *Bmal* mRNA expression was set to CT 21 h.

To examine whether simulations of the model are in agreement with biological phenotypes of the clock, we compared the peak phases of the in silico mRNA expression patterns of core-clock genes and the PER/CRY protein complexes with experimental data retrieved from the literature [1]. The peak phases for all core-clock genes are within the range of published experimental peak phases of core-clock mRNAs (Fig 2B, Table 2). Furthermore, the model successfully reproduces the correct phase relations among the core-clock components (Fig 2C).

**Table 2:** In silico phase differences of core-clock genes in the mathematical model.

**Core-clock Elements**	Peak Phase (h)
***Bmal***	21.00
***Rev-Erb***	3.80
***Ror***	10.97
***Per***	11.30
***Cry***	12.52
**PER/CRY pool**	16.32

The phase of *Bmal* (CT 21) is taken as reference with the period length set to 23.65 h. Shown are the in silico peak phases for all core-clock genes and the protein complex PER/CRY. CT, circadian time.

### The model reproduces the Ink4a/Arf^-/-^ associated changes in the circadian period

Data from our previous work points to a role of RAS as a regulator of the circadian clock period [32] as was also reported by other studies [61]. With our novel mathematical model, we investigated whether the observed RAS-induced change in the circadian clock period (Figs 1B and 2C) can be simulated both in the *Ink4a/Arf* WT and in the knock-out condition (Fig 2D). For the simulation, we adapted a method from our previous work on RAS-mediated dysregulation of the circadian clock in cancer, in which a factor *ktt* was introduced to the activation/inhibition rates describing CLOCK/BMAL-mediated transcription: *ktt* = 1 describes a normal RAS expression level whereas *ktt* < 1 indicates a reduction in the transcriptional activity of CLOCK/BMAL caused by RAS overexpression [32].

The double knock-out of *Ink4a/Arf* was achieved by setting the initial conditions of INK4a and ARF mRNAs, cytoplasmic and nuclear proteins, as well as their rate of change to 0 (S1 Text, equations 1, 2, 8, 10, 14, 19 = 0). We measured the period in our model for a transient region, defined as the mean of the time between the first four peaks (three periods) after introducing the perturbation of RAS (represented by *ktt* < 1) to the system. In this transient region, there are still fluctuations of the modelled system that can represent the observed biological noise of retrovirus-mediated RAS overexpression. More information concerning the model analysis can be found in S1 Text.

The model predicts a slightly longer circadian period for the Ink4a/Arf^-/-^ system (23.68 h compared to the adjusted period of 23.65 h in the WT) when RAS is expressed at WT levels (*ktt* = 1; Fig 2D), which results in a phase shift over time (S3A Fig). As expected from the experimental data shown in Fig 1 and our previously published results [32], there is a lengthening of the period upon RAS overexpression in Ink4a/Arf^+/+^ MEFs (Fig 2D). Interestingly, the opposite effect on the period is predicted by simulations in the Ink4a/Arf^-/-^ system. Thus, the in silico period changes are in agreement with the experimentally measured phenotypes in Ink4a/Arf^-/-^ MEFs and their WT littermates (Fig 1B and 1C) and show the same tendency of an increase/a decrease of the period length in response to RAS overexpression. The simulations were not fitted to exactly reproduce the values of the experimentally measured periods, which were obtained from MEFs originating from different mice and therefore show a certain biological variation. The simulated Ink4a/Arf^-/-^ system shows a nonmonotonic dependency of the period length on the strength of the RAS overexpression. With increasing RAS, the period length first decreases, reaching its minimum for *ktt* = 0.7, only to increase afterwards in response to even higher simulated levels of RAS (0.4 *< ktt <* 0.7; Fig 2D). After introducing the perturbation of RAS to the system by varying the parameter *ktt*, there are nonmonotonic phase shifts of *Bmal* expression that depend on the strength of RAS overexpression (S1 Text). The change of the *ktt* value causes *Bmal* oscillations to peak at different times: The system with the strongest RAS overexpression and lowest simulated *ktt* value (*ktt* = 0.4) peaks the earliest for the first iteration but is then superseded by the system with *ktt* = 0.7 for the following iterations. The WT system always peaks last. These phase shifts are especially prominent for the Ink4a/Arf^-/-^ system, which represents one possible explanation for the observed nonmonotonic period changes (Fig 2D). Yet the effect of RAS overexpression on the length of the period is also dependent on the time when the perturbation is introduced to the model system (S3B Fig). Interestingly, the model predicts a longer period for an inhibition of RAS (*ktt* > 1) in the Ink4a/Arf^-/-^ system (S1 Text). We were able to confirm this prediction experimentally by inhibiting RAS in the Ink4a/Arf^-/-^ MEFs by using a MEK inhibitor that blocks the downstream chain in the RAS signalling pathway. Contrary to the shortened period observed when overexpressing RAS, we now observed an increase of the period (T = 23.76 ± 0.1 h for Ink4a/Arf^-/-^ MEFs and T = 25.24 ± 0.1 h for Ink4a/Arf^-/-^ -RAS MEFs; *n* = 3; mean and SEM; S1C–S1E Fig). Furthermore, we simulated the overexpression of RAS in the Ink4a/Arf^+/+^ condition (*ktt* = 0.6). The model predicts that RAS overexpression leads to an increase of the expression level of *Ink4a* as compared to the normal RAS scenario (Fig 2E). This is in line with published data showing that RAS activates *Ink4a*, leading to cell cycle arrest [24,27] and correlates with the RAS-induced increased number of senescent cells shown in Fig 1I. Moreover, we downregulated RAS in the Ink4a/Arf^-/-^ MEFs and observed a long period phenotype, as opposed to the period change observed for RAS overexpression (S1C–S1E Fig). Furthermore, we used an RAS-inducible construct to investigate the effects of different levels of RAS induction. Our data indicates that both longer and shorter periods can be observed, in a RAS-dependent manner, in agreement with the simulations from our mathematical model (S1F–S1J Fig).

Taken together, the results demonstrate that the model reproduces important circadian properties, which are in agreement with experimental data, and that it can be used to further elucidate the mechanism of RAS-induced and *Ink4a/Arf*-dependent changes of the circadian period.

### Modular analysis of the role of the INK4a and the ARF pathway in mediating the RAS-induced effect on the circadian clock

In order to investigate the relative influence of the INK4a/RB1/E2F1 (module 1) and ARF/MDM2/p53 (module 2) pathways in mediating the RAS-induced effect on the core-clock in silico, we tested whether the presence of key elements in the network and the oscillations of both modules are necessary to reproduce the experimentally determined clock period phenotypes of Ink4a/Arf^+/+^ and Ink4a/Arf^-/-^ MEFs.

**Fig 3:**
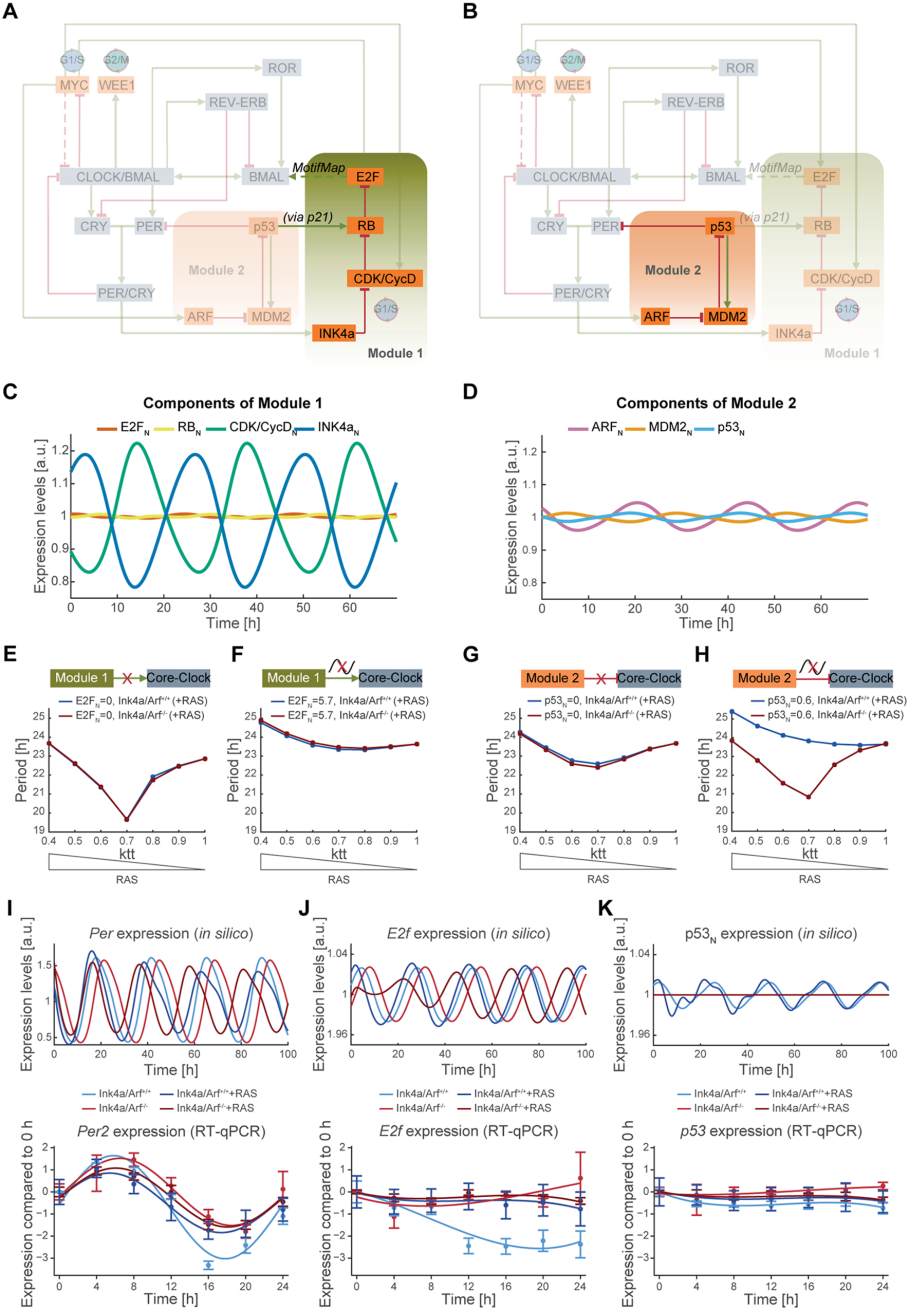
The oscillation of the INK4a/RB1/E2F1 pathway is necessary to mediate the RAS-induced change of the circadian phenotype. The importance of the INK4a/RB1/E2F1 pathway (module 1) (A) and the ARF/MDM2/p53 pathway (module 2) (B) in influencing the circadian period is analysed by simulating different scenarios in silico. The simulated expression profiles for components of module 1 (C) and module 2 (D) show oscillations with different amplitudes and peak phases. Decoupling of module 1 from the core-clock (E) and clamping the oscillatory expression of its component E2F_N_ to its constitutive average value (F) lead to similar changes in the circadian period for the Ink4a/Arf^+/+^, and the Ink4a/Arf^-/-^ condition as does decoupling of module 2 from the core-clock (G). (H) The simulation of a nonrhythmic module 2 by clamping its component p53_N_ to its constitutive average value predicts a change in the circadian period under Ink4a/Arf^+/+^ conditions compared to the knock-out counterpart, indicating that the presence of both modules and the oscillation of the INK4a/RB1/E2F1 pathway are necessary to mediate the RAS-induced change of the circadian period. Represented are the period phenotypes in Ink4a/Arf^+/+^ (blue line) and Ink4a/Arf^-/-^ (red line) systems upon RAS overexpression (*ktt* < 1) compared to normal conditions (*ktt* = 1). The in silico expression levels of *Per* (I), *E2f* (J), and p53 _N_ (K) are compared to 24-h time course RT-qPCR measurements of *Per2*, *E2f* and *p53* in four different conditions (Ink4a/Arf^+/+^, Ink4a/Arf^+/+^+RAS, Ink4a/Arf^-/-^, and Ink4a/Arf^-/-^+RAS; *n* = 2–3; mean and SD). For *Per2*, a harmonic regression and for *E2f* and *p53*, a polynomial (degree 4) is fitted to the data. Numerical values are provided in S1 Data.

As shown in Fig 3A, module 1 represents the connection of INK4a to the clock via the INK4a-dependent inhibition of E2F, a transcription factor that we predict to regulate the transcription of *Bmal* in the model. Still, we cannot exclude the possibility that such a regulation may happen via additional elements that were not investigated within the scope of this study (S3 Text). However, additional elements would not change the validity of the conclusions derived from the model as long as the delays in the expression values between the defined core-clock elements remain. These delays or phase differences, which were retrieved from published experimental data, were used as constraints in our model (S1 Text). Module 2 (Fig 3B) represents the ARF-mediated activation of the transcription factor p53, which is known to repress the CLOCK/BMAL-mediated transcription of *Per2*. In both modules, the nuclear protein elements show oscillations in their simulated expression patterns according to the circadian rhythm of the core-clock system (Figs 3C and 1D).

The connection of module 1 to the core-clock was disrupted by setting the concentration of E2F_N_ to 0 (S1 Text, equations 7, 12, 22 = 0; initial concentration of E2F_N_ = 0), thereby removing its predicted regulation of *Bmal* in the model. *Ink4a/Arf* knockout was modelled as described above. Upon the disconnection of module 1, both the Ink4a/Arf^+/+^ and the Ink4a/Arf^-/-^ system acquire shorter periods when RAS is overexpressed (*ktt* = 0.7) as compared to normal RAS conditions (*ktt* = 1; Fig 3E). These results are not in line with our previous simulations for the full network (Fig 2D) and the experimental observations (Fig 1A and 1B), which show an *Ink4a/Arf*-dependent effect on the period upon RAS overexpression. Thus, it seems that in our modelling scenario the predicted connection between E2F and *Bmal* is indeed necessary to reproduce the observed period changes. When comparing the expression and the period length of *Bmal* oscillations before and after the perturbation by RAS, it becomes evident that the knockout of module 1 results in a lower period value of 22.86 h for both the Ink4a/Arf^+/+^ and the Ink4a/Arf^-/-^ system. This results in a phase shift of *Bmal* oscillations when compared to the oscillations in the WT, causing the differing effect on the *Bmal* period length upon the perturbation by RAS (S1 Text). We further investigated the relevance of the oscillations in module 1 for the RAS-mediated effect on the circadian period. The connection between E2F_N_ and *Bmal* was maintained, but the expression of E2F_N_ was clamped to the constitutive concentration of its mean value (S1 Text, E2F_N_ = 5.7, equations 7, 12, 22 = 0). The constant expression of E2F leads to similar period lengths of 23.63 h for the Ink4a/Arf^+/+^ and the Ink4a/Arf^-/-^ systems (Fig 3F). In this case, RAS overexpression causes a nonmonotonic change of the period length depending on the value of *ktt*, but independent of the *Ink4a/Arf* status resulting in slightly shorter periods for 0.7 ≤ *ktt* < 1 and longer periods for 0.4 ≤ *ktt* < 0.7. Again, this does neither reproduce the results of the previous simulation nor the experimental observations. *Bmal* oscillation profiles of both systems shows that there is only a very small phase shift between the Ink4a/Arf^+/+^ and the Ink4a/Arf^-/-^ systems, potentially explaining why both systems react similar upon perturbation by RAS (S1 Text). These results indicate that in the model, both the connection of the INK4a/RB1/E2F1 module to the core-clock via E2F regulation of *Bmal* and the low-amplitude oscillations of E2F are crucial to simulate the contrary effect of RAS overexpression on the circadian period.

Next, we tested whether the connection of module 2 to the core-clock is also necessary to reproduce the experimentally observed phenotype by setting the concentration of the component p53_N_ to 0 (S1 Text, equation 16 = 0). The expression profile of *Bmal* shows that the perturbation is introduced at a similar oscillation phase for both systems, which differs from that of the WT *Bmal* oscillations (S1 Text). As before, both the Ink4a/Arf^+/+^ and the Ink4a/Arf^-/-^ system show a nonmonotonic change of the period dependent on the strength of the simulated RAS overexpression: for 0.7 ≤ *ktt* < 1, there is a decrease in period length, followed by a slight increase for 0.4 ≤ *ktt* < 0.7. This does not reproduce the experimentally observed RAS-induced period changes indicating that the connection of the ARF/MDM2/p53 pathway to the core-clock is crucial as well. To simulate a constitutive connection of module 2 to the core-clock, we clamped the oscillatory expression of p53_N_ in module 2 to its average expression value (S1 Text, equation 16 = 0.6). Interestingly, by using a constitutive concentration of p53_N_, we were able to simulate circadian phenotypes similar to the simulations of the whole network and the experimental observations (Fig 3H). Although p53 is no longer oscillating, there is a similar phase shift between the Ink4a/Arf^+/+^ and the Ink4a/Arf^-/-^ system as in the original (S1 Text). This suggests that the oscillation of the ARF/MDM2/p53 pathway plays only a minor role in regulating the period in response to RAS overexpression and can instead be substituted by a constitutive expression.

Taken together, these results indicate that upon RAS overexpression, the connections of both the ARF/MDM2/p53 and the INK4a/RB1/E2F1 pathway to the core-clock are necessary to produce the observed period phenotypes. The simulations from the mathematical model predict that a circadian expression of the INK4a/RB1/E2F1 pathway is crucial for reproducing the *Ink4a/Arf*-dependent change in rhythmicity in response to RAS overexpression. This again points to dynamical effects such as phase shifting in gene expression, which are hard to identify experimentally because even small phase shifts can cause large effects in a feedback loop.

Furthermore, we simulated the expression of representative components of the core-clock (*Per*), module 1 (*E2f*), and module 2 (p53_N_) under four different conditions (Ink4a/Arf^+/+^, Ink4a/Arf^+/+^+RAS, Ink4a/Arf^-/-^, and Ink4a/Arf^-/-^+RAS) and compared them to experimental 24-h time-course measurements of *Per2*, *E2f*, and *p53* under the same conditions. The knockout of INK4a and ARF and the overexpression of RAS (*ktt* = 0.6) were modelled as described above. Both the in silico simulations and the experimental measurements show that *Per* is oscillating with a circadian rhythm in all conditions (Fig 3I). As expected, there is a phase shift in the simulated *Per* expression upon the knockout of *Ink4a* and *Arf*, with the knockout peaking slightly later than the WT, which is in line with the phase shift observed in the simulations of *Bmal* expression (S3A Fig). The same tendency can be observed in the experimental time-course measurements of *Per2*. RAS overexpression reduces the amplitude of *Per* oscillations both in the WT and the Ink4a/Arf^-/-^ system (Fig 3I). *E2f* and *p53* exhibit only low expression changes. The simulated expression patterns of *E2f* show oscillations with similar amplitudes in all four conditions while in the experimental measurements, the WT has a higher fold change than the perturbed conditions (Fig 3J). The in silico expression of p53_N_ shows low amplitude oscillations in the WT conditions (both with and without RAS overexpression), which are out-of-phase with Per oscillations (S1 Text) in agreement with a recent work that modelled the spatiotemporal regulation of p53 by Per2 [39]. In the knockout condition, its expression is constitutive and does not change upon overexpression of RAS (Fig 3K), which is reflected in the low fold change observed for *p53*. This supports our prediction that the constitutive, but not the oscillatory expression of the ARF/MDM2/p53 pathway is necessary to reproduce the *Ink4a/Arf*-dependent effect on the circadian period upon RAS overexpression as shown in the modular analysis.

### Genome-wide analysis of expression levels in perturbed MEFs shows a dependency of the RAS-induced effect on Ink4a/Arf

To further investigate the hypothesis that *Ink4a/Arf* plays a crucial role in the RAS-mediated effect on the core-clock with propagating effects on clock-regulated genes, we conducted a genome-wide screening of gene expression upon RAS overexpression and *Bmal1* downregulation using microarrays of Ink4a/Arf^+/+^ and Ink4a/Arf^-/-^ MEFs under the eight different conditions summarised in Fig 1A. The experimental setup was validated by reverse transcription quantitative PCR (RT-qPCR) for *Bmal1*, *Ink4a/Arf*, and the oncogene *HRas* (S1B Fig).

**Fig 4:**
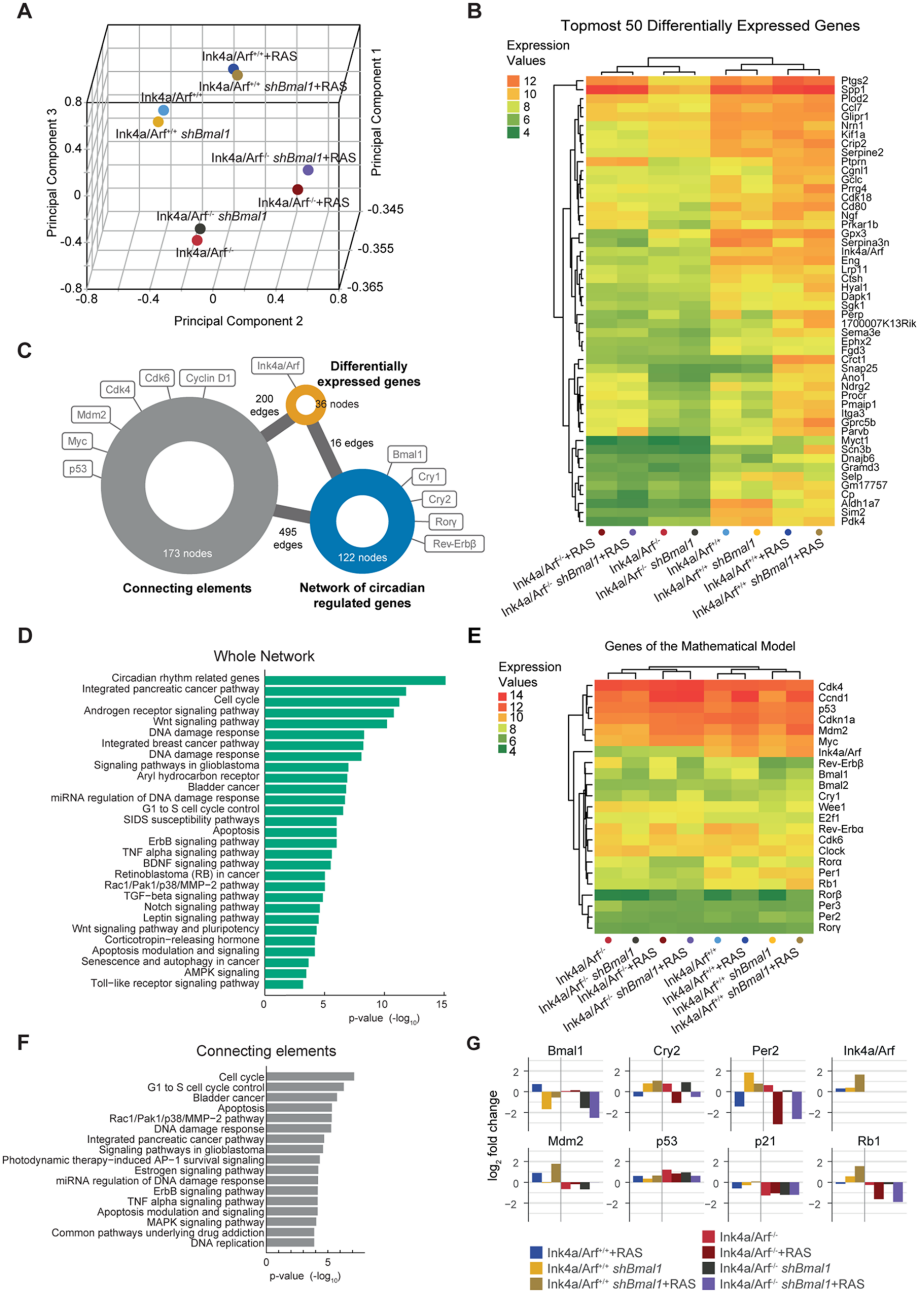
The genome-wide effect of RAS overexpression and Bmal1 downregulation on Ink4a/Arf ^+/+^ and Ink4a/Arf ^-/-^ MEFs can be mirrored by genes from the mathematical model. (A) The first three principal components as determined by genome-wide expression analysis based on microarray data of Ink4a/Arf^+/+^ and Ink4a/Arf^-/-^ MEFs with different perturbations highlighting the differences between the eight experimental conditions. The arrays cluster in four groups depending on the presence of *Ink4a/Arf* and RAS overexpression. (B) Based on the four groups determined in the PCA, the top 50 differentially expressed genes across the different experimental conditions were determined. (C) A network connecting 36 out of the topmost 50 differentially expressed genes (orange) to the previously published NCRG (blue) by at most one connecting element (grey) was generated using information from the IntAct database. The genes that are part of the mathematical model are highlighted. An enrichment analysis was performed to determine the most highly represented pathways for the whole network (D) and the set of connecting elements (F). (E) The clustering based on the expression levels of genes from the mathematical model mimics the genome-wide clustering. (G) The expression changes of selected core-clock and cell cycle-related genes upon perturbations by RAS and *shBmal1* in Ink4a/Arf^+/+^ and Ink4a/Arf^-/-^ MEFs were validated by RT-qPCR and visualised as the log_2_ fold change compared to the WT MEFs. Numerical values are provided in S1 Data. MEF, mouse embryonic fibroblasts; NCRG, network of circadian regulated genes.

We carried out a principal component analysis (PCA) based on genome-wide expression values (Fig 4A, S2 Fig). The eight conditions were separated into four groups along the first three principal components. Notably, the conditions with downregulated *Bmal1* are grouped with the corresponding WT counterparts, indicating a limited effect of the *Bmal1* knockdown at the whole genome level. Upon RAS overexpression, Ink4a/Arf^+/+^ MEFs and Ink4a/Arf^-/-^ MEFs are visibly separated, reinforcing our hypothesis that *Ink4a/Arf* influences the RAS-mediated effect on the system.

To explore possible correlations between components of the circadian clock and the genes most affected by RAS overexpression and/or knockdown of *Bmal1* in Ink4a/Arf^+/+^ and Ink4a/Arf^-/-^ MEFs, we used molecular interaction data from the IntAct database [62] to create a network of the two gene sets. The first gene set consists of the top 50 differentially expressed genes whose expression levels exhibit the highest variance across the four groups determined by the PCA (Fig 4B). The list of the topmost 50 differentially expressed genes is provided in S1 Table. Interestingly, 32 out of the top 50 differentially expressed genes have been shown to oscillate in the suprachiasmatic nucleus (SCN) or other peripheral tissues, such as liver, kidney, or lung, according to the database of mammalian circadian gene expression, CircaDB [63] (S1 Table). The second gene set is comprised of 166 clock and clock-controlled genes that constitute our previously published network of circadian regulated genes (NCRG) [64].

For the network, we only included elements of the NCRG connected to the set of differentially expressed genes via a direct interaction or via one connecting element and further reduced the network to one connected component. The resulting network consists of three gene/protein sets that form an undirected graph of 331 nodes, 36 of which are part of the original differentially expressed genes and 122 are part of the NCRG (S4 and S5 Figs). The remaining 173 nodes are connecting elements of the two gene sets. The genes from the mathematical model are present in all three subsets of the network (Fig 4C). Components of modules 1 and 2 can be found in the set of differentially expressed genes *(Ink4a/Arf*) and the set of connecting genes (*Cdk4*, *Cdk6*, and *Cyclin D1* of module 1 and *p53* and *Mdm2* of module 2) in addition to *Myc*. Five core-clock genes are part of the reduced NCRG (*Bmal1*, *Cry1*, *Cry2*, *Rorγ*, and *Rev-Erbβ*). The significance of the number of direct interactions between the differentially expressed genes and the circadian clock was tested by comparing the network properties to those of 100 randomly generated networks based on the set of differentially expressed genes and a set of 122 randomly chosen genes. This resulted in a considerably lower average of 3 ± 2 interactions (mean and SD) between the set of differentially expressed genes and the random gene sets as compared to 34 interactions with the NCRG based on data by iRefIndex [65].

To analyse the biological significance of the network, we used consensuspath.db to determine enriched Wikipathway terms (Fig 4D and 4F). As expected, circadian rhythm-related genes are the topmost enriched pathway. In addition, there is an overrepresentation of pathways related to various types of cancer such as pancreatic cancer, breast cancer, bladder cancer, glioblastoma, and retinoblastoma. Furthermore, there are several enriched pathways that are related to carcinogenesis, including the Wnt signalling pathway (*Gsk3β*, *Apc*), the ErbB pathway (*Erbb2*, *Gsk3*, *Myc*), the TNFα pathway (*Nfkb*, *Mapk3*), the Notch signalling pathway (*Ncor1*,*2*), and the TGF-β signalling pathway (*Smad4*).

Additionally, several cell cycle-related pathways are among the topmost enriched terms of the whole network including contrasting cell cycle-fate phenotypes, such as apoptosis signalling, G1 to S cell cycle control, and senescence. The enrichment analysis for the set of connecting elements yields similar results as the analysis for the whole network, but includes also the MAPK signalling pathway and DNA replication among its overrepresented pathways (Fig 4F).

The high number of direct interconnections and connections via one intermediate element between the two gene sets indicates a strong interplay between the mammalian circadian core-clock and the genes that were differentially expressed upon RAS overexpression and/or knockdown of *Bmal1* in Ink4a/Arf^+/+^ and Ink4a/Arf^-/-^ MEFs. The presence of various cell cycle-related genes in the set of connecting components in addition to the results of the enrichment analysis of overrepresented pathways hints at an involvement of the cell cycle in this connection. The enrichment analysis places the network connections into a potential cancer context that involves genes affected by disturbances of the circadian system and RAS oncogenic signalling, in addition to cell cycle-related genes.

### Gene expression analysis of genes from the mathematical model

To specifically investigate the connection between components of the circadian clock and the cell cycle and the effects of RAS overexpression and *Bmal1* downregulation in the system, we analysed the expression levels of 23 genes included in our mathematical model (Fig 4E, S2 Table) and validated the results for selected genes with RT-qPCR (Fig 4G). The clustering based on the gene set mimics the overall separation on the genome-wide level as determined by the PCA (Fig 4A) and is specific for the selected genes when compared to random sets of the same size (*p* = 4.54e-07). Notably, the changes in expression of clock and cell cycle-related genes upon RAS overexpression and downregulation of *Bmal1* differ between Ink4a/Arf^+/+^ MEFs and their Ink4a/Arf^-/-^ littermates.

As expected, *Ink4a/Arf* itself is up-regulated upon RAS overexpression as predicted by the model (Fig 2E), which is in agreement with published data [24]. This increase is higher when combined with the downregulation of *Bmal1*. When knocking out *Ink4a/Arf*, the connection between the clock and cell cycle via the activation of *Ink4a* by the NONO-PER protein complex is severed. This leads to an increase in the expression levels of several core-clock genes such as *Cry2* and *Per2*, while the expression level of *Bmal1* does not change significantly (Fig 4G). The knockout of *Ink4a/Arf* differentially influences a number of cell cycle genes. Despite being inhibited by *Arf*, expression of the oncogene *Mdm2* decreases upon *Ink4a/Arf* knockout, which in turn, leads to an increase of the expression levels of the tumour-suppressor gene *p53* (Fig 4G). The consistent up-regulation of *p53* in all knock-out conditions leads to a decrease in the expression levels of the tumour suppressors *p21* and *Rb1*, which might ultimately result in the proliferation effect observed for the Ink4a/Arf^-/-^ MEFs (Fig 1G).

Perturbation of the circadian clock by knockdown of the core-clock gene *Bmal1* leads to an increase in *Cry2* and *Per2* expression levels. However, the distinct up-regulation of *Per2* levels in Ink4a/Arf^+/+^ MEFs is not found in Ink4a/Arf^-/-^ MEFs. Interestingly, the tumour suppressor genes *p53* and *Rb1* are up-regulated upon *Bmal1* knockdown, which points to a possible compensation of the tumour-suppressor role of the circadian system.

RAS overexpression in the WT MEFs decreases the average expression levels of most core-clock genes, such as *Cry2* and *Per2* with the exception of *Bmal1*, which shows an increased expression (Fig 4E and 4G). This fits with our previously published data in which we showed that overexpression of RAS decreases the activity of BMAL1 [32], which should lead to a lower transcriptional activation of elements of the *Per* and *Cry* families. In the Ink4a/Arf^-/-^ MEFs, we observed a stronger RAS-induced downregulation of *Cry2* and *Per2*, indicating that the influence of RAS on the circadian clock is indeed mediated by *Ink4a/Arf*. RAS overexpression concurrent with *Bmal1* downregulation leads to an increase in the expression levels of the clock genes *Cry2* and *Per2* and the cell cycle genes *Mdm2* and *Rb1* in Ink4a/Arf^+/+^ MEFs, whereas upon knockdown of *Ink4a/Arf*, the opposite effect can be observed for *Cry2*, *Per2*, and *Rb1* (Fig 4G). This shows that the effects of RAS overexpression and *Bmal1* downregulation of the expression levels of clock and cell cycle-related genes can differ between Ink4a/Arf^+/+^ and Ink4a/Arf^-/-^ MEFs, particularly when both perturbations are combined.

### Predicted classification of the Ink4a/Arf^+/+^ and Ink4a/Arf^-/-^ MEFs’ proliferation/senescence state is in line with experimental results

**Fig 5:**
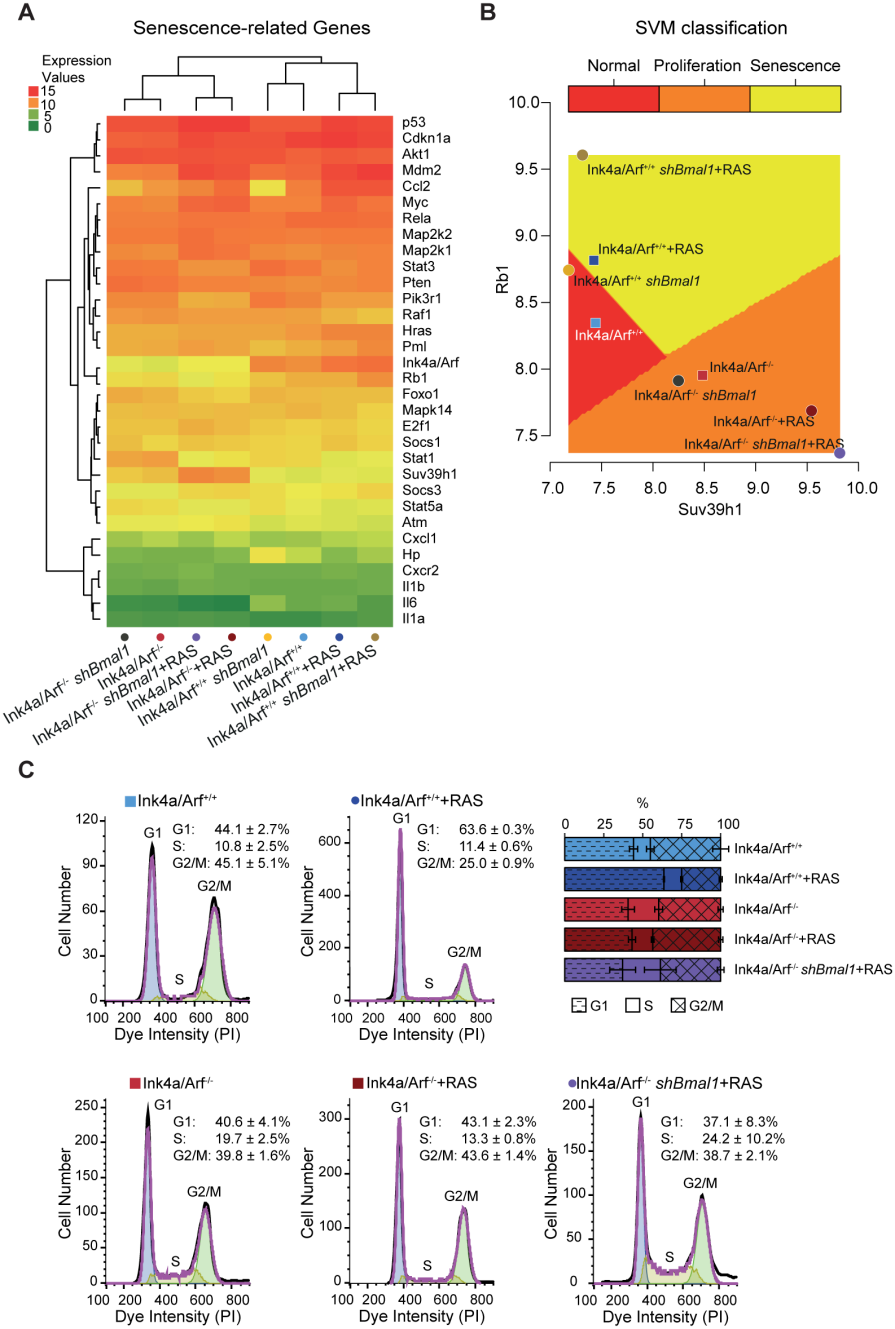
Predictive classification of cell cycle-fate phenotypes for Ink4a/Arf^+/+^ and Ink4a/Arf^-/-^ MEFs based on the expression of senescence-associated genes can be validated by experimental results. (A) A set of 32 senescence-related genes derived from the literature clusters according to the knockout of *Ink4a/Arf*. (B) Two-dimensional representation of the SVM classification based on the expression of the senescence-related genes *Rb1* and *Suv39h1* for the three training conditions (represented as squares) and the remaining five predicted conditions (represented as dots). (C) FACS analysis to determine the percentage of cells in each cell cycle phase for the three training conditions (Ink4a/Arf^+/+^, Ink4a/Arf^+/+^+RAS, and Ink4a/Arf^-/-^) and two of the predicted conditions (Ink4a/Arf^-/-^+RAS and Ink4a/Arf^-/-^ *shBmal1*+RAS) (*n* = 3; mean and SEM). The cell cycle phases were determined by fitting a univariate cell cycle model using the Watson pragmatic algorithm. Shown are representative examples for each condition. Numerical values are provided in S1 Data. FACS, fluorescence-activated cell sorting; MEF, mouse embryonic fibroblasts; PI, propidium iodide.

Given the strong influence of the *Ink4a/Arf* knockout on several clock and cell cycle genes, we further analysed the expression across the different experimental conditions of 32 manually curated genes associated with senescence [66–69] (S2 Table, Fig 5A).

In order to predict whether the Ink4a/Arf^+/+^ and Ink4a/Arf^-/-^ MEFs exhibit normal, proliferating,or senescent behaviour upon RAS overexpression and/or downregulation of *Bmal1*, a machine learning analysis with a support vector machine (SVM) was performed. The SVM algorithm creates a model based on information from a set of training conditions to predictively classify the remaining conditions based on the expression of the 32 senescence-associated genes. For the training set, we selected conditions with strong cell cycle-fate phenotypes as determined by the experimental data: Ink4a/Arf^+/+^ (normal), Ink4a/Arf^+/+^+RAS (senescence), and Ink4a/Arf^-/-^ (proliferation; Fig 1G and 1I). A two-dimensional representation of the resulting classification based on the expression of the genes *Rb1* and *Suv39h1*, a histone methyltransferase known to be essential for senescence [70], is shown in Fig 5B.

Ink4a/Arf^+/+^ *shBmal1* MEFs were classified as normal, whereas senescent behaviour was predicted for Ink4a/Arf^+/+^ *shBmal1*+RAS MEFs. This is in line with the experimental results of the SA-β-gal staining, which showed a high percentage of senescent cells in Ink4a/Arf^+/+^ *shBmal1*+RAS MEFs, but not for Ink4a/Arf^+/+^ *shBmal1* MEFs (Fig 1I) and similar cell growth for Ink4a/Arf^+/+^ *shBmal1* MEFs and their WT littermates (Fig 1G). The Ink4a/Arf^-/-^ conditions were all predicted to be proliferating. Experimentally, both Ink4a/Arf^-/-^ and Ink4a/Arf^-/-^ *shBmal1* MEFs have been shown to proliferate (Fig 1G). For the remaining conditions, we conducted a fluorescence-activated cell sorting (FACS) analysis to determine the percentage of cells in each cell cycle phase and compared them to those of the three training conditions (Ink4a/Arf^+/+^, Ink4a/Arf^+/+^+RAS, Ink4a/Arf^-/-^; Fig 5C).

FACS analysis of the WT MEFs yields a distribution of 44.1 ± 2.7% (*n* = 3, mean and SEM) cells in G1 phase, 10.8 ± 2.5% in S phase, and 45.1 ± 2.7% cells in G2/M phase. The Ink4a/Arf^+/+^+RAS MEFs show a higher percentage of cells in G1 phase (63.6 ± 0.3%) as opposed to a lesser number of cells in G2/M phase (25.0 ± 0.9%) as expected for senescent cells arrested in G1 phase. In contrast, the Ink4a/Arf^-/-^ MEFs that show a proliferating phenotype (Fig 1G) have a larger number of cells in S phase (19.7 ± 2.5%) than the WT. Similarly, Ink4a/Arf^-/-^ shBmal+RAS MEFs have a higher percentage of cells in S phase (24.2 ± 10.2%) than the WT, indicating a proliferating behaviour, as do Ink4a/Arf^-/-^+RAS MEFs, albeit to a smaller extent (13.3 ± 0.8%) (Fig 5C). This shows that the expression data of the 32 senescence-associated genes suffices to correctly predict the senescence/proliferation state of Ink4a/Arf^+/+^ and Ink4a/Arf^-/-^ MEFs upon different perturbations.

In order to compare our observations regarding the interplay between the clock and the cell cycle in the MEFs system to human experimental models, we additionally analysed available microarray gene expression data from different human cells (see Materials and methods). These include the human primary fibroblast cell line IMR-90 and the human colorectal cancer (CRC) cell lines RKO, LIM1215, Caco2, HCT116, SW403, HT29, COLO205, SW480, and SW620.

H-Ras expressing IMR-90 cells undergo oncogene-induced senescence [71] in agreement with our observations in the MEFs system. In the published array set (GEO-GSE33613), the downregulation of *ERK2* in H-Ras expressing IMR-90 cells causes the cells to bypass senescence and to proliferate instead. We analysed this dataset and found that both *Bmal1* and *Ink4a/Arf* were downregulated in the proliferating cells when compared to the H-Ras expressing IMR-90 cells as observed in the MEF cells (S6A Fig).

We further analysed available array data of human CRC cells from our own group (GEO-GSE46549) [2] and analysed the expression of all the genes, which are represented in our mathematical model. The comparison of the resulting heat map (S6F Fig) with the one of Fig 4E shows large differences between the cancer cells and the primary MEF cells analysed. Moreover, the genetic profile is very different in between the CRC cells.

In the metastatic SW620 cells, we measured an up-regulation of *Ink4a/Arf* and a downregulation of *HRas* and *Bmal1* as compared to the primary SW480 cells (S6B Fig). Furthermore, we carried out cell cycle measurements by FACS for both cell lines, which show an increase in the number of cells in S-phase and a corresponding decrease in the percentage of cells in G1-phase for SW620 (S6E Fig). This points to an increase of proliferation associated to a reduction of *Bmal1* despite an increased level of *Ink4a/Arf* in these cells. We further downregulated *Bmal1* in both SW480 and SW620 and analysed the subsequent effects on the cell cycle (S6C and S6D Fig). We measured an increase of approximately 7.5% of cells in the S-phase and a similar decrease of cells in the G1-phase for the SW480 cells (S6E Fig). No significant effect was observed in the SW620 cells in which *Bmal1* was already at low levels before its downregulation (S6B Fig). It seems that the decreased expression levels of *Bmal1* are associated to more proliferative scenarios in these cell lines.

Altogether, our results reinforce the hypothesis that *Ink4a/Ar*f—being a part of the cellular response upon RAS overexpression—acts as a regulator for the oncogene-induced effect on the clock phenotype and cell cycle-fate decision.

## Discussion

In this study, we explore a potential mechanism via which the oncogene RAS is able to dysregulate the circadian clock and the interplay of this interaction with the cell-division cycle. We build up upon previously published data, in which we showed that the overexpression of RAS in cancer cell lines leads to a lengthening of the clock period while its downregulation causes a shortening [32]. RAS is also known as an elicitor of cell cycle decisions depending on the presence of the tumour suppressor genes *Ink4a* and *Arf* [23]. Moreover, recent data points to an additional connection between the circadian clock and cell proliferation via the NONO-PER protein complex that activates the rhythmic transcription of *Ink4a* [46]. Hence, we hypothesise that the RAS-dependent dysregulation of the circadian clock might be achieved via elements involved in the cell cycle that are interlocked with the circadian system. We explored this idea in silico, by means of a novel mathematical model which connects the core-clock and the cell cycle, and experimentally, using an *Ink4a/Arf* knock-out mouse model system.

### RAS perturbs the circadian clock in an Ink4a/Arf-dependent manner

We analysed the circadian clock phenotype of WT MEFs and their littermates carrying a knockout of the tumour suppressors *Ink4a* and *Arf*, under different conditions. While the clock period is similar for the WT and the knock-out MEFs, both cell types show an RAS-dependent response of the clock phenotype with an opposite effect on the period length. As expected from our previous results [32], the WT Ink4a/Arf^+/+^ MEFs exhibit a longer period upon RAS overexpression. Interestingly, in the Ink4a/Arf^-/-^ MEFs, RAS overexpression causes a shortening of the period. We hypothesise that this differential effect might be due to a phase shift in the expression of circadian elements that is induced by the knockout of *Ink4a/Arf*. The cell cycle checkpoint and tumour suppressor gene *Ink4a/Arf* seems to influence the RAS-mediated effect on the clock phenotype, pointing to a cross-talk between the core-clock and the cell cycle in an oncogenic-dependent manner.

### The in silico results point to the role of Ink4a/Arf as a bridging element between the circadian clock and the cell cycle

A number of cell cycle regulators such as *Myc*, *Wee1*, and *Ink4a/Arf* are known to be clock-controlled [18,19], but the complex molecular mechanisms that couple these two biological oscillators are not entirely understood. Deeper insights into the underlying mechanisms might be of great advantage for understanding how a disruption of our internal timing system might induce malignant proliferation of cells in cancer.

Thus, to investigate the interplay between the circadian clock and cell cycle elements, we developed a novel mathematical model of the mammalian core-clock that includes the tumour suppressors *Ink4a/Arf*, as well as core-clock elements and cell cycle checkpoint genes, some of which have been reported to be directly regulated by the circadian clock as described above.

The model predicts RAS-induced *Ink4a/Arf*-dependent changes in the length of the clock period, as well as an RAS-induced increase of *Ink4a* transcription levels, which correlates with the experimentally observed cell cycle arrest phenotype. For the model construction, we assumed that *Bmal1* transcription can be regulated by E2F as an additional coupling element between the clock and the cell cycle based on candidate regulatory motif sites and published data. Indeed, if we increase the strength of the E2F activation on *Bmal* in the model (see control coefficient analysis, S1 Text) we obtain a longer period. This is consistent with the increase of *Bmal1* transcription in the WT MEFs after RAS induction and the longer period phenotype observed. The postulated connection between E2F activators and *Bmal1* is an interesting topic that needs further investigation.

Our in silico data show that the *Ink4a/Arf*-dependent changes in circadian phenotypes upon RAS overexpression might be due to phase shifts in the oscillations of the core-clock and subsequently also in cell cycle components. A small phase shift between the WT and the knock-out conditions observed in the modelling simulations could also be detected in time-course measurements of the core-clock gene *Per2*.

In addition, an in silico analysis that introduced RAS overexpression at different time points showed that the time of the perturbation might be essential for the differing effects on the period phenotype—a finding that deserves further experimental investigation and could have consequences regarding chronotherapy research [16], but goes beyond the scope of this study. By independently removing the ARF/MDM2/p53 pathway and the INK4a/RB1/E2F pathway from the model, we show that the presence of both pathways is necessary to reproduce the experimentally observed RAS-mediated dysregulation of the circadian phenotype. Although components of both modules oscillate in the WT simulations, the modular analysis showed that circadian oscillations of the INK4a/RB1/E2F module are sufficient to simulate the observed RAS-dependent changes in the period phenotypes. This indicates that the INK4a/RB1/E2F1 pathway is necessary for maintaining the rhythmicity of the system and that its connection to the clock might be rate limiting to control cell proliferation.

Our mathematical model describes a free-running clock, which is consistent with the experimental approach in which MEFs are placed under constant conditions. As a potential future perspective, it would be interesting to investigate how our conclusions would vary when cells are subjected to synchronising cues such as metabolic signals linked to the feeding/fasting cycles, e.g., by integrating our clock-cell cycle model with a recent model of the mammalian liver clock and metabolic sensors [72].

### High-throughput differential gene expression analysis points to a strong connection between the circadian clock and cell cycle elements

The tumour suppressor role of INK4a has been reported in different studies, e.g., *Ink4a*-deficient mice develop spontaneous melanomas and are more susceptible to carcinogens than WT mice [73]. Moreover, the tumour suppressor p53 is known to modulate *Per2*—a clock gene that interacts with NONO and *Ink4a*—therefore, it is likely that these components are relevant for controlling clock-dependent cell proliferation [30,39]. To get a deeper insight into the consequences of disturbing the coupling between the cell cycle and the core-clock, we performed a whole-genome analysis using MEFs from Ink4a/Arf^-/-^ mice and their WT littermates.

The genome-wide PCA of the expression data yields a separation of the Ink4a/Arf^+/+^ MEFs and Ink4a/Arf^-/-^ MEFs into four main groups. The knockout of *Ink4a/Arf* has the strongest effect, followed by the perturbation by RAS. The clustering of a smaller gene set derived from genes of the mathematical model yields a similar separation of Ink4a/Arf^+/+^ MEFs and Ink4a/Arf^-/-^ MEFs as does the genome-wide PCA. This indicates that the genome-wide differences caused by RAS overexpression and *Bmal1* downregulation are well mimicked in our interacting subset of clock and cell cycle genes, and reinforces the postulated interconnection between the core-clock and the selected cell cycle elements. To explore the putative effect of these correlations on the circadian system, we determined the interconnections between the 50 topmost differentially expressed genes and a set of circadian-regulated genes, recently published by our group [64]. The resulting network reveals a strong interplay of the two sets via protein–protein and protein–nucleic acid interactions, which are complemented by an additional third set of newly retrieved connecting elements. Remarkably, components of our mathematical model are distributed among the set of connecting elements, the topmost differentially expressed genes and the set of circadian-regulated genes, emphasising the strong coupling between the circadian system and cell cycle components. This synergy is further reinforced by the pathway enrichment analysis of the whole network, which highlights several cell cycle-related terms as well as cancer-related effector pathways.

### The core-clock as a RAS-dependent enhancer of cell cycle arrest

In our MEF model system, the overexpression of the oncogene RAS leads to an increased expression of the core-clock gene *Bmal1*. Even in the *Bmal1* knock-down condition, the *Bmal1* levels increase upon RAS overexpression, though they are still lower than in the WT, indicating a direct influence of RAS on clock components. Here, it is important to notice that *Bmal1* may influence biological processes in different ways: by disrupting circadian rhythms, and/or by influencing the expression of genes, which are direct targets of CLOCK/BMAL1 as a result of other potential, noncircadian functions of *Bmal1*. Therefore, the output results of *Bmal1* knock-down phenotypes must be seen in light of this wider perspective.

We further observed *Ink4a/Arf*-dependent changes in the expression levels of core-clock and cell cycle-related genes: RAS overexpression in combination with *shBmal1* leads to an increase of *Cry2* and *Per2* when *Ink4a/Arf* is present, whereas it results in its downregulation in the knock-out MEFs. The concurrent perturbations (overexpression of RAS and simultaneous knockdown of *Bmal1*) lead to an additive effect on the expression levels of genes of the *Per* family. This indicates that *Ink4a/Arf* may be involved in regulating the gene expression of core-clock genes upon RAS overexpression, including but not limited to members of the *Per* family. This correlates with changes in the expression levels of the cell cycle-related gene *Rb1* that is also up-regulated in the Ink4a/Arf^+/+^ *shBmal1*+RAS MEFs and downregulated in the corresponding knock-out condition. We speculate that upon RAS-induced dysregulation of the core-clock, the expression level changes of the clock gene *Per2* might lead to the differential regulation of *Rb1*. In addition, the predicted loss of oscillations of p53 upon *Ink4a/Arf* knockout could account for the downregulation of *Rb1* in the knock-out MEFs and be the cause for the observed experimental variations in *Rb1* gene expression upon perturbations by RAS and *shBmal1*. Ultimately, this could result in abnormal cell fate decision phenotypes, including cell-cycle arrest, which would also be in agreement with published data [74] and points to a correlation between the core-clock gene *Bmal1* and cellular senescence.

Based on a curated list of senescence-related genes derived from the literature and on our results from β-Gal-staining and growth measurements, we used a machine learning approach to classify the cell cycle fate decision of our experimental model system upon perturbations by RAS overexpression and dysregulation of the circadian system.

While in the Ink4a/Arf^+/+^ MEFs overexpression of RAS leads to oncogene-induced senescence, the Ink4a/Arf^-/-^ MEFs are classified as proliferating despite the up-regulation of the oncogene RAS. We validated the conditions that were predicted to proliferate by cell-cycle analysis and compared them to the WT and the senescent conditions. The downregulation of *Bmal1* shows an enhancing effect of the *Ink4/Arf* knockout-induced proliferation phenotype, resulting in the highest percentage of cells in the S phase and lowest in G1 phase.

**Fig 6:**
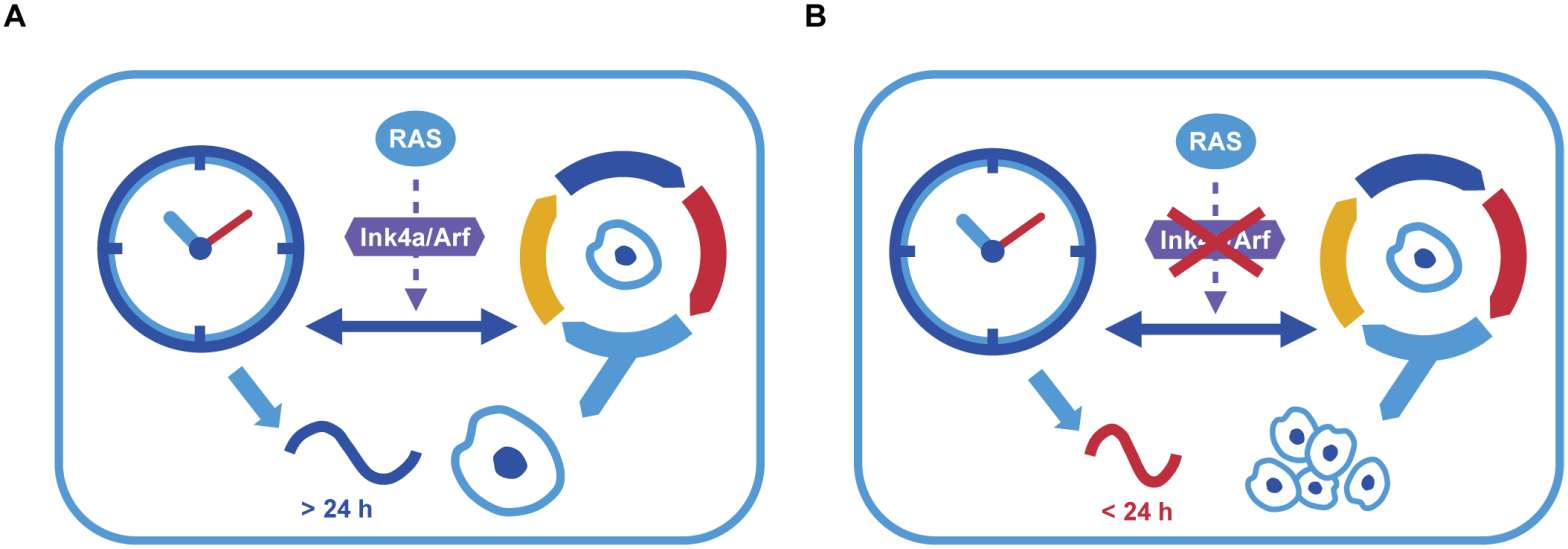
Schematic model of the Ink4a/Arf-RAS interplay and its connections to circadian clock and cell cycle phenotypes. (A) Overexpression of the oncogene RAS results in a lengthening of the circadian period in WT MEFs and leads to a senescent cell phenotype. (B) Knockout of the tumour suppressor element *Ink4a/Arf* that connects components of both cellular oscillators, leads to a change in the RAS-induced effect on the clock resulting in a shorter circadian period and proliferation of cells. MEF, mouse embryonic fibroblasts; RAS, rat sarcoma viral oncogene; WT, wild-type.

Altogether, our experimental and modelling analyses strongly point to a role for *Ink4a/Arf* in mediating the clock connection to important cell cycle checkpoints and controlling its response upon RAS oncogenic signalling. This led us to propose a model for the interplay between the cell cycle and the circadian clock that highlights *Ink4a/Arf* as an important regulator for the period-changing effect of RAS on the circadian clock as depicted in Fig 6. The *Ink4a/Arf* locus seems to act as a switch for the effect of RAS. If the *Ink4a/Arf* genes are functional while the system is perturbed by the oncogenic signalling, the period increases and the cells receive a senescence signal (Fig 6A). If on the contrary, *Ink4a/Arf* genes are impaired, then the period of the system decreases upon RAS perturbation, and the cells receive a proliferation signal (Fig 6B). Whereas some studies have questioned the role of the clock as a tumour suppressor [13], our data is in line with several publications including a recent work in which enhancing circadian clock function seems to control cancer progression [75], and points to clock dysregulation as a tumourigenesis-promoting factor. It is conceivable that the tumourigenesis promoting/inhibiting properties of the circadian clock are context dependent, and to unravel the full complexity of such interactions will require a tumour-specific circadian analysis.

Our combined theoretical and experimental approach using a primary cell model system interrelates high-throughput data with mathematical modelling, which together provide compelling evidence for the control of both cellular oscillators, the cell division cycle, and the circadian clock via the oncogene RAS. Furthermore, our data points to the existence of a fine-tuning system of circadian regulation via *Ink4a/Arf*, as a response to RAS overexpression with severe consequences on cell fate decisions.

To see whether our results hold true for other systems as well, we analysed the expression of *Bmal1* and *Ink4a/Arf* in several publicly available human cells, e.g., fibroblasts and CRC cell lines with RAS overexpression or *Bmal1* downregulation. We found that RAS overexpression leads to increased levels of *Ink4a/Arf* and *Bmal1* in the senescent human fibroblast cell line IMR-90 as compared to their proliferative counterparts. In the CRC cell line SW480 (derived from the primary tumour), *Bmal1* downregulation by shRNA lead to an increase of cells in S-phase and a decrease of cells in G1-phase resembling the cell-cycle phenotype observed in the metastasis-derived cell line SW620, in which *Bmal1* was already downregulated independent of the shRNA.

Based on our results, it seems to us that the clock is likely to act as a tumour suppressor, and that it is of advantage for cancer cells to circumvent circadian control. One cannot stop wondering whether disrupted circadian timing should be included as a next potential hallmark of cancer.

## Materials and methods

### Mathematical model

The semiquantitative mathematical model was derived by using the same approach as in our previously published model of the mammalian circadian core-clock [1,32]. It contains 46 variables and 170 parameters. A detailed representation of the underlying network is shown in S2 Fig. The model comprises a set of 45 ODEs, which were implemented using Matlab R2015a (MathWorks, Natick, MA), with ODE45, a built-in solver for non-stiff differential equations by using a Runge-Kutta method. Both the relative error tolerance and the components of the absolute error tolerance vector were set to 10^−9^. An integration step of 0.01 was used. The system of equations was assembled by using Hill-type kinetics, mass action kinetics, and Michaelis-Menten kinetics. The model was parameterised following extensive literature research. The remaining free parameters were either determined analytically or fine-tuned to fit known core-clock phase relationships (delays) for the WT scenario. Refer to S1 Text for detailed lists of model variables, parameters, and equations.

### Lentivirus production and transduction of MEFs with the Bmal1:Luc reporter and Bmal1 shRNA

HEK293T cells (human, kidney, ATCC Number: CRL-11268) were seeded in a 75 cm^2^ cell culture flask and transfected with 6 μg of psPAX packaging plasmid, 3.6 μg pMD2G envelope plasmid, and 8.4 μg of either *Bmal1*-promoter-driven luciferase expression plasmid, pLKO.1 *shBmal1* or nonsilencing *shBmal1* control (Dharmacon Inc., Lafayette, CO) using the CalPhos-mammalian transfection kit (BD Biosciences, San Jose, CA). Virus particles were harvested and spun at 4000 x g for 15 minutes to remove cell debris. Supernatant was passed through a 0.45 μm filter (Sarstedt Group, Newton, NC) and used for lentiviral transduction. WT and KO primary MEFs were isolated from embryos as previously described [76] and cultured in DMEM (Gibco Laboratories, Gaithersburg, MD) containing 10% fetal calf serum (Sigma-Aldrich, St. Louis, MO) and 1% penicillin-streptomycin (Merck Millipore, Burlington, MA). Cells were incubated at 37°C at 3% O_2_, 5% CO_2_. MEFs were transduced with 1.5 ml virus filtrate plus 8 μg/μl protamine sulfate (Sigma-Alrich) in 35 mm dishes. After 1 day, the medium was replaced with selection medium (*Bmal1*:*Luc* hygromycin 100 μg/ml, *shBmal1* puromycin 10 μg/ml).

### Cell culture of SW480 and SW620 cells

SW480 (human, colon, ATCC Number: CCL-228) and SW620 (human, colon, ATCC Number: CCL-227) cell lines were maintained in DMEM low glucose (Lonza Group, Basel, Switzerland) culture medium supplemented with 10% FBS (Life Technologies, Durham, NC), 1% penicillin-streptomycin (Life Technologies), 2 mM Ultraglutamine (Lonza Group), and 1% HEPES (Life Technologies). Cells were incubated at 37°C in a humidified atmosphere with 5% CO_2_.

### Transduction of the CRC cell lines SW480 and SW620 with Bmal1 shRNA

A TRC lentiviral shRNA glycerol set (Dharmacon) specifically for *Bmal1* was used consisting of five individual shRNAs. The construct that gave best knock-down efficiency was determined by gene expression analysis and used for further experiments. The transduction was carried out as indicated above for the MEFs.

### Cell culture and retroviral infection

IMR90 human diploid fibroblasts (HDFs) (human, lung, ATCC Number: CCL-186) were cultivated in DMEM medium, 10% FBS, 100 U/ml penicillin-streptomycin, and transduced with retroviral construct pBabe-Ras-BSD or pBabe-empty-BSD as control. After blasticidine selection, cells were harvested at different days and cell pellets were frozen in −80°C for RNA extraction.

### Retrovirus production for RAS overexpression

Low-passage Phoenix cells were grown in a 10-cm petri dish to a maximal density of 70%. 20 μg MSCV-Ras-BSD plasmid (MSCV Retroviral Expression System; Clonetech Laboratories, Mountain View, CA, and human hRas cDNA, Dharmacon), 15 μg helper plasmid, and 62.5 μl CaCl2 were mixed and adjusted with sterile water to 500 μl. Subsequently, 10 ml DMEM medium containing 25 μM chloroquine and the precipitate was added. After 12 h of incubation, virus particles were collected. MEF cells were seeded at subconfluent density 12 h after Phoenix cell transfection. The first virus supernatant was harvested within 12 h after transfection by aspiration and filtered through a 0.45 μm filter. The virus supernatant including 4 μg/ml polybrene was added to the MEFs after medium change. After 12 h of incubation, the second virus supernatant was harvested as described above, supplemented with polybrene and added to the MEFs. After spinoculation of the plates (1,500 rpm, 10 min, 32°C), cells were grown until the next round of transduction. The third and fourth virus supernatants were collected 12 h and 24 h later according to the same procedure. Following the last transduction, selection medium containing 10 μg/ml blasticidin was added and cells were selected. The protocol underlying the animal work, in compliance with Federation of European Laboratory Animal Sciences Association’s (FELASA) guidelines, was reviewed and approved by the Landesamt für Gesundheit und Soziales (LAGeSo), Berlin, with the identification number G105/10.

### Inhibition of Ras-MEK-ERK signalling

For RAS-inhibition assays, 2 × 10^5^ cells were plated in 35 mm dishes and cultured overnight. On the next day, the cells were synchronised by a single pulse of 1 μM dexamethasone (Sigma-Aldrich) for 45 min after which an MEK inhibitor (UO126 final concentrations 10 μM; Promega Corporation, Durham, NC) was added to the culture together with 2 ml of reporter medium containing luciferin (250 μM final concentration; PJK GmbH, Kleinblittersdorf, Germany). Luciferase activity was monitored for five days using a photomultiplier tube (PMT)-based device (LumiCycle; Actimetrics Inc., Evanston, IL). The Chronostar software was used for data analysis [77].

### Inducible Ras overexpression

For RAS-overexpression assays, 2 × 10^5^ cells were transduced with a construct encoding *H-RAS*^*G12V*^ as described previously [41] were plated in 35 mm dishes and cultured in medium containing 4-hydroxytamoxifen (4OHT) (Sigma-Aldrich) was used at 1 nM, 10 nM, and 100 nM for 5 days. On the sixth day, cells were synchronised by a single pulse of 1 μM dexamethasone (Sigma-Aldrich) for 45 min. Individual dishes were washed with phosphate-buffered saline, and 2 ml of reporter medium containing luciferin (250 μM final concentration, PJK GmbH) was added per dish, after which 4OHT was added again to the culture in the concentrations as previously described. Ethanol was used as a solvent control.

### Synchronisation of MEFs and monitoring of the Bmal1 promoter activity

Ink4a/Arf^+/+^ and Ink4a/Arf^-/-^ MEFs (from five WT mice and KO littermates) harbouring the *Bmal1*-promoter-driven luciferase reporter construct (two independent transductions per condition) were synchronised with dexamethasone (1 μM final concentration, Sigma-Aldrich) for 45 min. Individual dishes were washed with phosphate-buffered saline, and 2 ml of reporter medium containing luciferin (250 μM final concentration, PJK GmbH) was added per dish. Luciferase activity was monitored for five days using a PMT-based device (LumiCycle, Actimetrics). The Chronostar software was used for data analysis [77].

### SA-β-galactosidase staining

MEF cells were seeded in 6- or 12-well plates containing 5 mm round glass cover-slips and incubated under standard conditions overnight to let the cells attach. Afterwards, medium was removed and cells were fixed in freshly prepared fixation solution (0.25% Glutaraldehyde, 2% Paraformaldehyde in PBS containing 1 mM MgCl_2_) at room temperature. After 15 min incubation, fixation solution was removed and cells were washed twice in phosphate-buffered saline. Staining solution was added and plates were transferred and incubated in a humidified atmosphere at 37°C for 16 h. Cells were gently washed with PBS and mounted afterwards. On each slide 200 cells were analysed, and the blue-stained cells were counted as positive.

### Chromatin immunoprecipitation and quantitative PCR (ChIP-qPCR) analyses

ChIP-qPCR assays were performed using the iDeal ChIP-seq kit (Diagenode s.a., Liège, Belgium) with a primer pair specific for the promoters of *Bmal1*. Primer sequences are available upon request.

### RNA extraction and gene expression analysis by RT-qPCR

Total RNA from MEFs was isolated with the RNeasy Mini kit (Qiagen, Hilden, Germany) following the manufacturer instructions. Microarray hybridisation was carried out by the Labor für funktionelle Genomforschung (LFGC, Charité—Universitätsmedizin Berlin) using Affymetrix Mouse Exon 1.0 ST arrays. For RT-qPCR analysis, the extracted RNA was reverse transcribed into cDNA by using random hexamers (Eurofins MWG Operon, Huntsville, AL) and Reverse Transcriptase (Life Technologies). RT-qPCR was performed using mouse QuantiTect Primer assays (Qiagen) and SYBR Green fluorescence assays (Life Technologies). For the detection of human *Ras* expression, a human Quantitect Primer assay (Qiagen) was used. RNA was analysed with a real-time PCR System (Applied Biosystems, Foster City, CA). For selected genes, we harvested MEFs around one circadian cycle in three-h intervals and performed a time-course RT-qPCR analysis. Gene expression levels were normalised to mouse *Gapdh* mRNA (Eurofins MWG Operon, fwd: ACGGGAAGCTCACTGGCATGGCCTT rev: CATGAGGTCCACCACCCTGTTGCTG). The log_2_-fold change in expression of the target genes in the different conditions (Ink4a/Arf^+/+^/Ink4a/Arf^-/-^ MEFs with and without *Ras* and Ink4a/Arf^+/+^/Ink4a/Arf^-/-^ MEFs with and without *shBmal1*) in relation to the control (Ink4a/Arf^+/+^) was calculated as ΔΔCт. For the time-course analysis, the log_2_-fold change was calculated in comparison to the expression at 0 h. The cosine function fitted to the time-course data was calculated with the R package HarmonicRegression [78].

For the human cell lines SW480, SW620, and IMR-90 RT-qPCR was performed by using human QuantiTect Primer assays (Qiagen) and SYBR Green (Bio-Rad Laboratories, Hercules, CA) in 96-well plates. Tbp or Gapdh were used as housekeeping genes. The qPCR reaction and the subsequent melting curve were performed using a real-time PCR Detection System (Bio-Rad). Cт values were determined by using the regression method. The log_2_-fold change in gene expression in relation to the respective control was calculated as ΔΔCт.

### FACS analysis

MEF cells (from three mice and KO littermates), SW480, and SW620 cells used for cell cycle evaluations were labelled with 10 μM of BrdU/1x10^6^ cells for 60 min, then collected and washed with 1 x PBS and fixed with ice-cold 80% ethanol. After DNA denaturation with 2 N HCl/Triton x-100, samples were neutralised with 0.1 M Na_2_B_4_O_7_, washed with PBS, and stained with 3 μL of anti-BrdU-FITC (BD Biosciences Clone B44), in a 1 x PBS solution containing 0.5% Tween20, 1% BSA, and 10 mg/mL of RNase (AppliChem GmbH, Darmstadt, Germany) for 1 h at room temperature. Supernatant was removed, cells resuspended in 200 μL of 1xPBS containing 5 μg/mL of PI (Sigma-Aldrich), and read in FACSCabilur (Becton Dickinson, Franklin Lakes, NJ). The cells of interest were gated based on forward scatter/side scatter (FSC versus SSC) values (S4 Text). The cell cycle analysis was conducted by fitting a univariate cell cycle model to the previously gated population using the Watson pragmatic algorithm [79] as implemented in FlowJo v10.2 (FlowJo, LLC, Ashland, OR).

### Microarray analysis

The microarray data was analysed in R version 3.2.3 using the oligo package [80]. Expression levels of genes were calculated using the Robust Multi-Array Average (RMA) preprocessing procedure [81]. Mouse exon arrays were annotated by using the moex10sttranscriptcluster.db package. Heatmaps of clustered gene expression were generated by using the ComplexHeatmap package [82]. Hierarchical clustering with Pearson correlation was used to build the heatmaps. The R packages arrayQualityMetrics [83], affycoretools, and ReportingTools [84] were used for quality control and statistical testing of the arrays (S2 Text).

The microarray dataset has been deposited in the ArrayExpress database at EMBL-EBI (www.ebi.ac.uk/arrayexpress) under the accession number E-MTAB-5943. The calculation of the principal components was performed with built-in functions of R and visualised with the scatterplot3d package. Differentially expressed genes were determined with the limma package [85]. Analysis of the statistical significance of the resulting clusters was performed with the SigClust package [86].

We also tested the expression of our genes of interest *(Ink4a/Arf*, *Bmal1*, *HRas*) in publicly available microarrays of different human cell lines: human primary fibroblasts (IMR-90) with stably expressing H-RasV12 and shRNA against *ERK2* or a nontargeting shRNA (GEO-GSE33613), and the CRC cell lines RKO, LIM1215, Caco2, HCT116, SW403, HT29, COLO205, SW480, and SW620 (GEO-GSE46549).

### Network generation and analysis

Gene and protein regulatory networks were generated using Cytoscape version 3.4 [87]. Additional information for the network was retrieved from the IntAct database of the EBI-EMBL [62] by mapping the NCBI Gene ID to Ensembl Gene IDs and Uniprot IDs. Pathway enrichment was performed with consensuspath.db. Random network analysis was performed with the iRefR package for R and iRefIndex version 14.0 with the IntAct database.

### SVM classification

In order to predict the senescence/proliferation behaviour of the different experimental conditions, a machine learning analysis was performed on a subset of the microarray data with the SVM algorithm of the e1071 R package. The dataset consists of 32 manually curated genes associated with senescence [66–69]. The conditions used as the training set were Ink4a/Arf^+/+^, Ink4a/Arf^+/+^+RAS, and Ink4a/Arf^-/-^+RAS.

## Supporting information

S1 Fig Effects of shBmal1 and RAS inhibition/induction in MEF cells. (A) The cell viability of wild type MEFs and Ink4a/Arf^-/-^ MEFs with and without shBmal1 was monitored over five days (n = 3; mean and SEM). (B) The knockdown of *Bmal1*, the knockout of *Ink4a/Arf* and the overexpression of RAS were validated by RT-qPCR. The expression level of *Bmal1* decreases to about 30% upon its downregulation with shRNA, as compared to the wild type value. (C) Summary of circadian period phenotype measurements for Ink4a/Arf^-/-^ MEFs with and without RAS inhibition (n = 3; mean and SEM). (D,E) RAS inhibition (Ink4a/Arf^-/-^-RAS) prolongs the period of Ink4a/Arf^-/-^ MEFs (25 h, orange) compared to the corresponding control (23.2 h, red). (F) Summary of circadian period phenotype measurements for Ink4a/Arf^-/-^ MEFs with and without induction of RAS with 4OHT (n = 2; mean and SEM). (G-J) RAS induction (Ink4a/Arf^-/-^+RAS, 4OHT = 1 nM, 10 nM, 100 nM) causes different effects on the period of Ink4a/Arf^-/-^ MEFs compared to the corresponding control (26.1 h, red). Numerical values are provided in S1 Data.
(PDF)

S2 Fig Detailed diagram of the mathematical model. The network comprises two compartments, the nucleus and the cytoplasm. There are 46 variables in total. For most gene entities, the mRNA (blue), cytoplasmic protein (purple) and nuclear protein (yellow) are distinguished. The transcriptional activation, phosphorylation/dephosphorylation processes are represented in green lines, the transcriptional repressions are represented by red lines. Translation and nuclear importation/exportation processes are represented by black lines while complex formation/dissociation processes are represented using brown lines.
(PDF)

S3 Fig In silico clock phenotype variation in an Ink4a/Arf-RAS-dependent manner. (A) *In silico* simulations show that the knockout system has a phase shift in the expression patterns of core-clock genes (represented by *Bmal*). (B) The time point of introducing RAS overexpression to the system influences the resulting period changes in the simulated wild type and the knockout systems.
(PDF)

S4 Fig Network of differentially expressed genes and clock-related genes. Network connecting 36 of the top 50 differentially expressed genes across the eight experimental conditions (red) with a set of 122 clock-related genes (blue) connected by 173 connecting elements (grey). Genes from the mathematical model are framed with a green line.
(PDF)

S5 Fig Cytoscape network of differentially expressed genes and clock-related genes. Cytoscape file of the network connecting 36 of the top 50 differentially expressed genes across the eight experimental conditions (red) with a set of 122 clock-related genes (blue) connected by 173 connecting elements (grey). Genes from the mathematical model are framed with a green line.
(CYS)

S6 Fig Interplay between the circadian clock and the cell cycle in human CRC cell lines. (A) The proliferative phenotype in IMR-90 cells (shERK2+RAS) vs its senescence counterpart (shCTRL+RAS) shows similar fold changes in *Bmal1* and *Ink4a/Arf* expression as compared to the MEFs system. Analysis from published microarray data (GEO—GSE33613). (B) A downregulation of *Bmal1* expression is observed in the metastatic CRC cell line (SW620) vs the primary tumour cell line (SW480). Analysis from published microarray data (GEO—GSE46549). (C,D) Downregulation of *Bmal1* leads to an increase of the tumour suppressor *Ink4a/Arf* in SW480 (RT-qPCR data: n = 3; mean and SEM). (E) FACS analysis to determine the percentage of cells in each cell cycle phase for the CRC cell lines SW480 and SW620 (control and shBmal1, n = 3; mean and SEM). The cell cycle phases were determined by fitting a univariate cell cycle model using the Watson pragmatic algorithm. (F) Heatmap for the genes of the mathematical model in human CRC cell lines. Analysis from published microarray data (GEO—GSE46549). Numerical values are provided in S1 Data.
(PDF)

S1 Table Top 50 differentially expressed genes across all eight conditions. The 50 topmost differentially expressed genes across the eight samples were determined with the R package limma based on the four clusters as determined by the PCA (p-value < 0.005). 32 of the genes were reported to be oscillating in CircaDB.
(XLSX)

S2 Table Expression values for genes from the mathematical model and for a curated list of senescence-related genes for all eight conditions. Log_2_-normalised expression values under all eight experimental conditions for 23 genes included in the mathematical model and for a curated list of 32 senescence-related genes based on literature research.
(XLSX)

S1 Text Description of the mathematical model. Detailed description of the mathematical model’s development, variables, parameters and equations. Additional model analysis and control coefficient analysis of the mathematical model parameters.
(PDF)

S2 Text Microarray quality control. Microarray data were subjected to standard statistical tests to assess their quality.
(PDF)

S3 Text Potential link between Clock/Bmal and E2f. (PDF)

S4 Text Gating strategies for the FACS analysis. Description of the gating strategies applied for the cell cycle analysis of the MEF cells and the SW480 and SW620 cells.
(PDF)

S1 Data Data overview for numerical values in figures. (XLSX)

## Abbreviations

4OHT: 4-hydroxytamoxifen
ARF: alternate open reading frame
Bmal: brain and muscle aryl hydrocarbon receptor nuclear translocator-like protein
CDK: cyclin-dependent kinase
CHIP-qPCR: chromatin immunoprecipitation and quantitative PCR
CLOCK: circadian locomotor output cycles kaput
CRC: colorectal cancer
CRY: cryptochrome
CT: circadian time
CycD: Cyclin D
FACS: fluorescence-activated cell sorting
FELASA: Federation of European Laboratory Animal Sciences Associations
G1: Gap 1
h: hour
HDF: human diploid fibroblast
LFGC: Labor für funktionelle Genomforschung
MAPK: mitogen-activated protein kinases
MDM2: mouse double minute 2 homolog
MEF: mouse embryonic fibroblasts
MYC: avian myelocytomatosis viral oncogene homolog
NCRG: network of circadian regulated genes
NONO: non-POU domain containing octamer binding
ODE: ordinary differential equation
PCA: principal component analysis
PER: period
PMT: photomultiplier tube
RAS: rat sarcoma viral oncogene
RB1: retinoblastoma-associated protein 1
Rev-erb: reverse strand of ERBA
RMA: Robust Multi-Array Average
ROR: RAR-related orphan receptor
RORE: ROR response element
RT-qPCR: reverse transcription quantitative PCR
SCN: suprachiasmatic nucleus
shRNA: short hairpin RNA
SVM: support vector machine
