# Supplementary figures and images for: The Ink4a/Arf locus operates as a regulator of the circadian clock modulating RAS activity
Source: PLoS Biol. 2017 Dec 7;15(12):e2002940. doi: 10.1371/journal.pbio.2002940 (PMC5720494; doi:10.1371/journal.pbio.2002940)

**Figure S1. Effects of *shBmal1* and RAS inhibition/induction in MEF cells.**

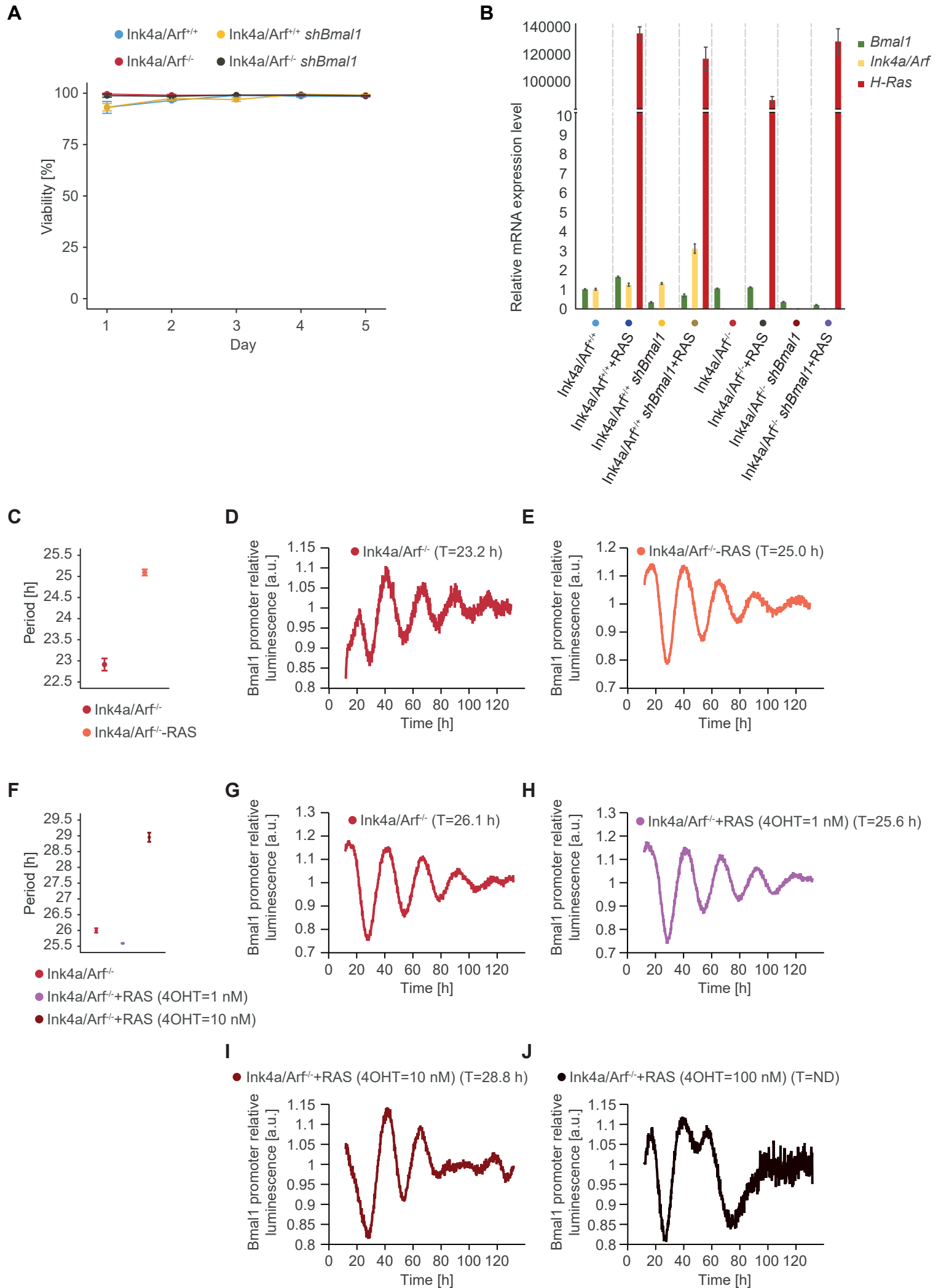

Supplement: S1 Fig — (A) The cell viability of wild type MEFs and Ink4a/Arf-/- MEFs with and without shBmal1 was monitored over five days (n = 3; mean and SEM). (B) The knockdown of Bmal1, the knockout of Ink4a/Arf and the overexpression of RAS were validated by RT-qPCR. The expression level of Bmal1 decreases to about 30% upon its downregulation with shRNA, as compared to the wild type value. (C) Summary of circadian period phenotype measurements for Ink4a/Arf-/- MEFs with and without RAS inhibition (n = 3; mean and SEM). (D,E) RAS inhibition (Ink4a/Arf-/--RAS) prolongs the period of Ink4a/Arf-/- MEFs (25 h, orange) compared to the corresponding control (23.2 h, red). (F) Summary of circadian period phenotype measurements for Ink4a/Arf-/- MEFs with and without induction of RAS with 4OHT (n = 2; mean and SEM). (G-J) RAS induction (Ink4a/Arf-/-+RAS, 4OHT = 1 nM, 10 nM, 100 nM) causes different effects on the period of Ink4a/Arf-/- MEFs compared to the corresponding control (26.1 h, red). Numerical values are provided in S1 Data. (PDF) [file pbio.2002940.s001.pdf]

Figure S2. Detailed diagram of the mathematical model.

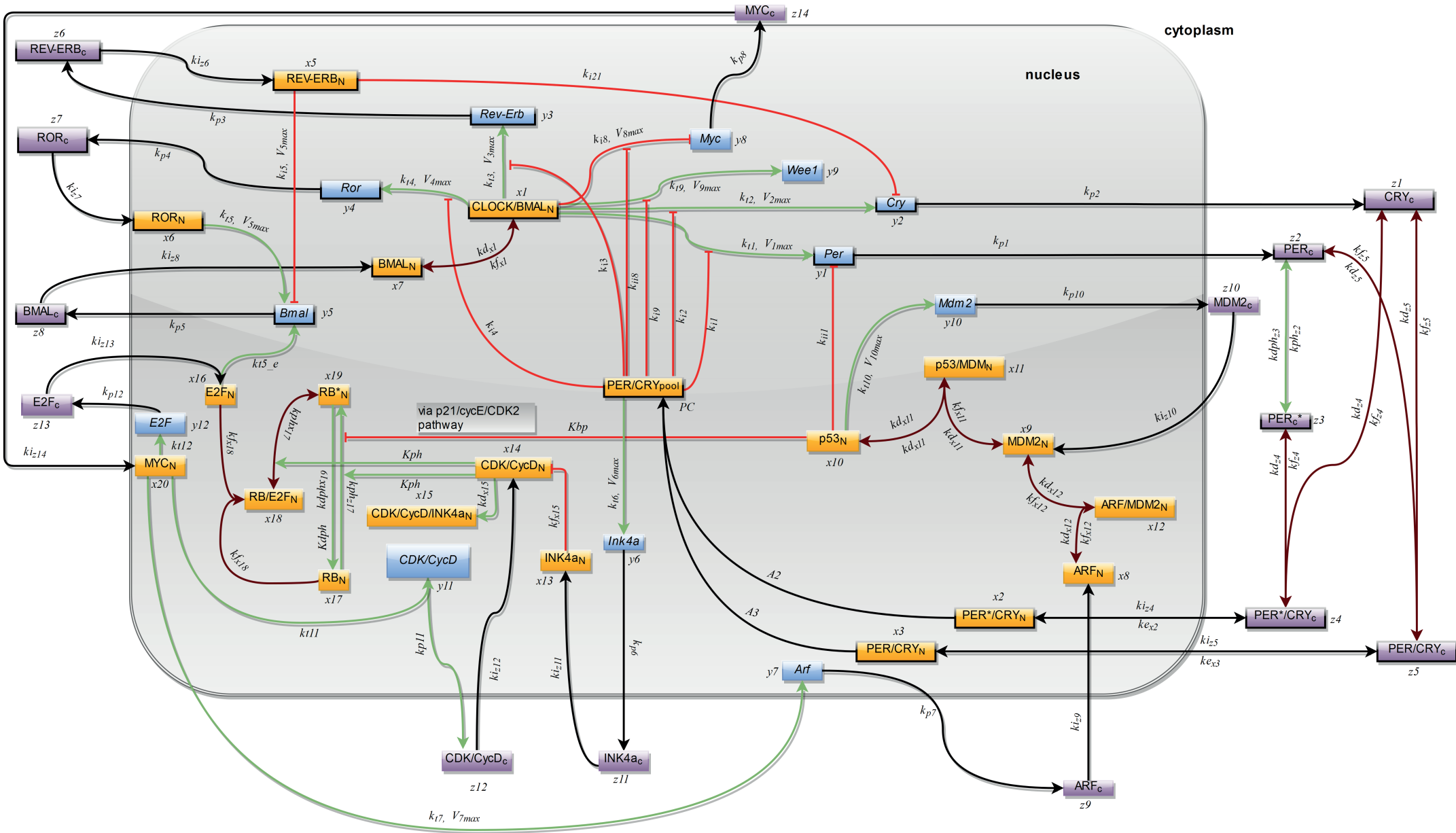

Supplement: S2 Fig — The network comprises two compartments, the nucleus and the cytoplasm. There are 46 variables in total. For most gene entities, the mRNA (blue), cytoplasmic protein (purple) and nuclear protein (yellow) are distinguished. The transcriptional activation, phosphorylation/dephosphorylation processes are represented in green lines, the transcriptional repressions are represented by red lines. Translation and nuclear importation/exportation processes are represented by black lines while complex formation/dissociation processes are represented using brown lines. (PDF) [file pbio.2002940.s002.pdf]

Figure S3. *In silico* period clock phenotype variation in an Ink4a/Arf-RAS- dependent manner.

A

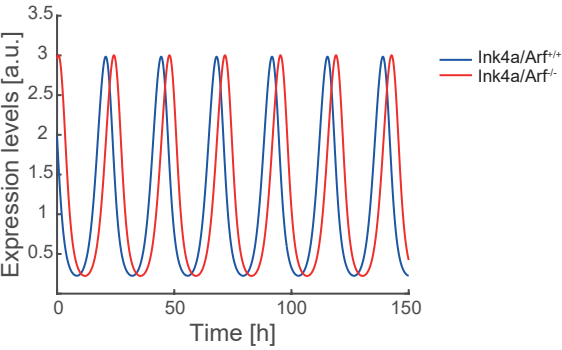

B

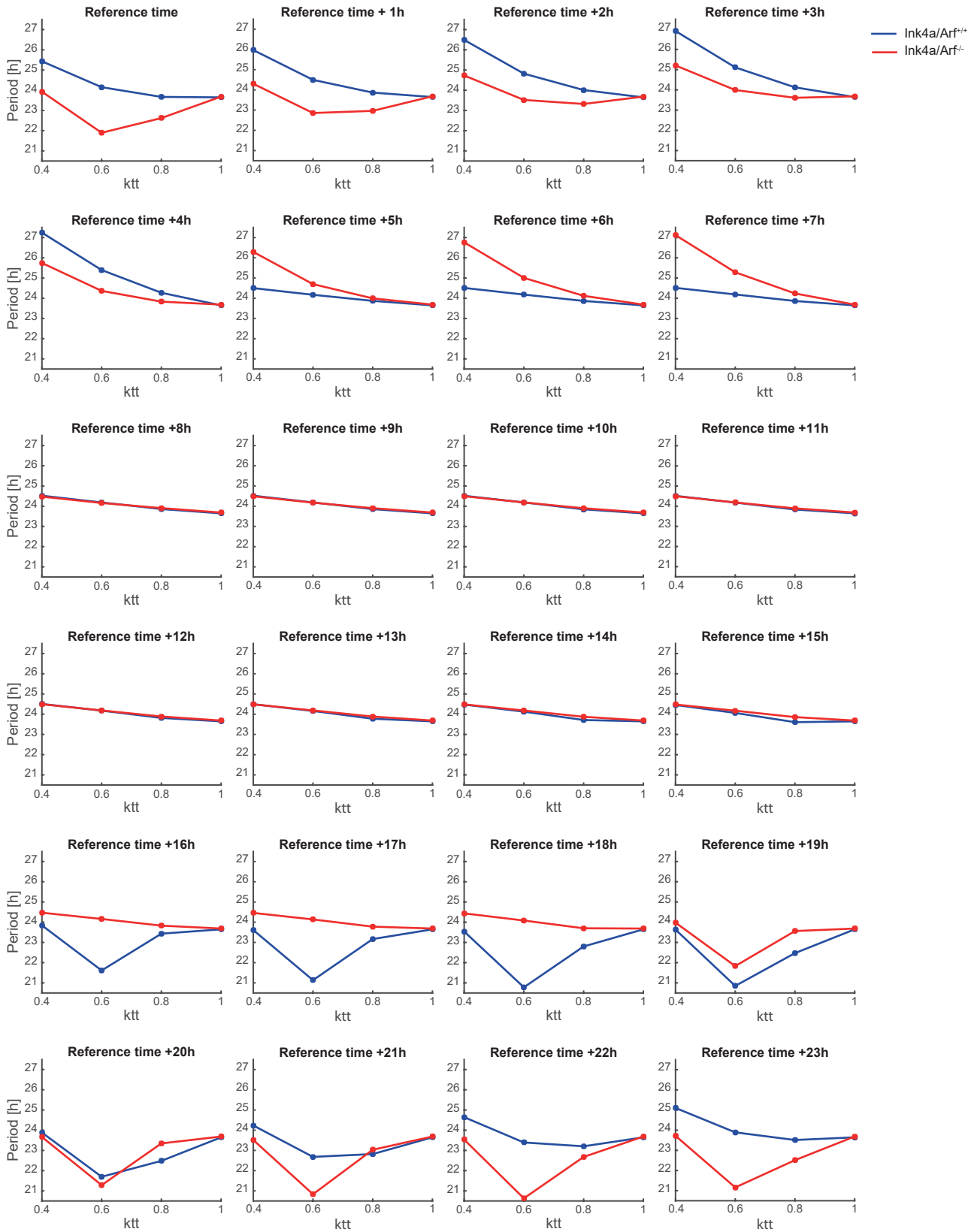

Supplement: S3 Fig — (A) In silico simulations show that the knockout system has a phase shift in the expression patterns of core-clock genes (represented by Bmal). (B) The time point of introducing RAS overexpression to the system influences the resulting period changes in the simulated wild type and the knockout systems. (PDF) [file pbio.2002940.s003.pdf]

Figure S4. Network of differentially expressed genes and clock-related genes.

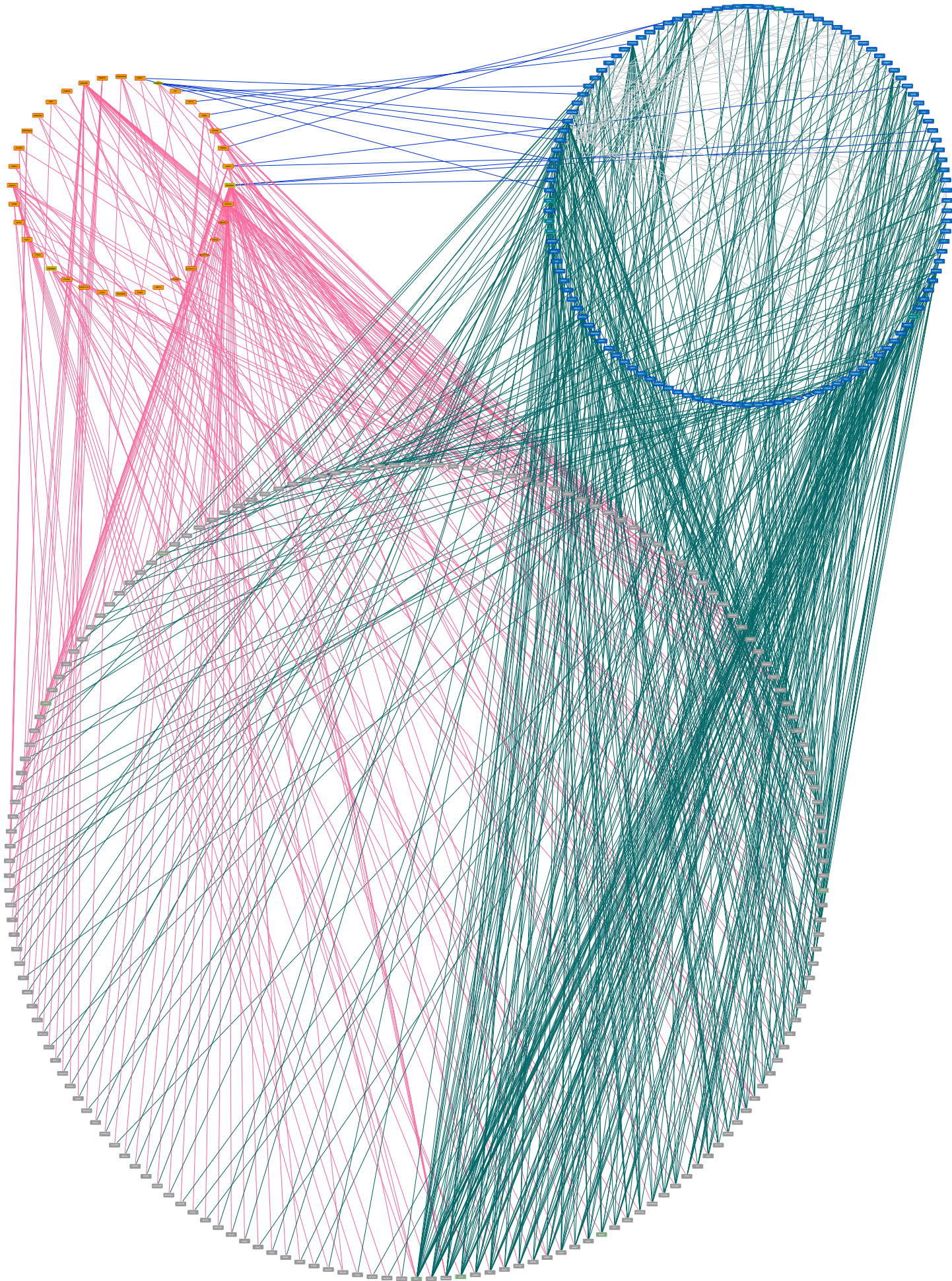

Supplement: S4 Fig — Network connecting 36 of the top 50 differentially expressed genes across the eight experimental conditions (red) with a set of 122 clock-related genes (blue) connected by 173 connecting elements (grey). Genes from the mathematical model are framed with a green line. (PDF) [file pbio.2002940.s004.pdf]

**A**

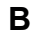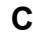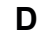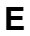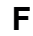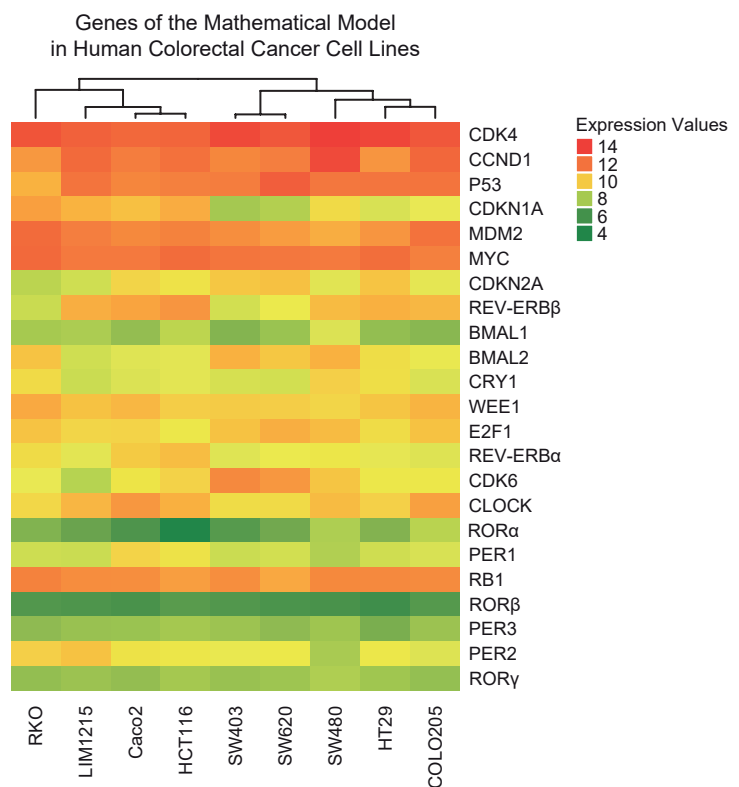

Supplement: S6 Fig — (A) The proliferative phenotype in IMR-90 cells (shERK2+RAS) vs its senescence counterpart (shCTRL+RAS) shows similar fold changes in Bmal1 and Ink4a/Arf expression as compared to the MEFs system. Analysis from published microarray data (GEO—GSE33613). (B) A downregulation of Bmal1 expression is observed in the metastatic CRC cell line (SW620) vs the primary tumour cell line (SW480). Analysis from published microarray data (GEO—GSE46549). (C,D) Downregulation of Bmal1 leads to an increase of the tumour suppressor Ink4a/Arf in SW480 (RT-qPCR data: n = 3; mean and SEM). (E) FACS analysis to determine the percentage of cells in each cell cycle phase for the CRC cell lines SW480 and SW620 (control and shBmal1, n = 3; mean and SEM). The cell cycle phases were determined by fitting a univariate cell cycle model using the Watson pragmatic algorithm. (F) Heatmap for the genes of the mathematical model in human CRC cell lines. Analysis from published microarray data (GEO—GSE46549). Numerical values are provided in S1 Data. (PDF) [file pbio.2002940.s006.pdf]
