# Supplementary material for: The Ink4a/Arf locus operates as a regulator of the circadian clock modulating RAS activity
Source: PLoS Biol. 2017 Dec 7;15(12):e2002940. doi: 10.1371/journal.pbio.2002940 (PMC5720494; doi:10.1371/journal.pbio.2002940)
Supplement: S1 Text — Detailed description of the mathematical model’s development, variables, parameters and equations. Additional model analysis and control coefficient analysis of the mathematical model parameters. (PDF) [file pbio.2002940.s009.pdf]

Supporting Information S1 Text – Model description, design and analysis

The supplementary Figure S1 represents a new model of the mammalian circadian clock. This model allows to investigate the coupling of the cell cycle to the circadian clock via the additional elements MYC, WEE1, INK4a and ARF. The circadian cell cycle regulation model (CCRM) is based on the published core-clock model (CCM) [1] from which 20 equations, 20 variables and 71 parameters were adapted. For the CCM, we used existent values for degradation rates, transcription rates etc. that were either retrieved from the literature or estimated based on known phases and amplitudes using LTI (linear-time-invariant) systems theory. First, we created a linear ODE version of both feedback loops in the network and applied LTI to the linearised system allowing for a partial determination of the parameters by an analytical calculation of amplitudes and phases as functions of the parameters. Each feedback loop was then closed, re-establishing the feedback. The parameters were optimised in order to achieve the optimal amplitude and phase-relations as retrieved from the literature. In a subsequent step, values for the corresponding parameters of the nonlinear system were determined using a Taylor expansion.

In the model, different members of one gene family are represented by a single composite variable: *Per* (*Per1,2,3*), *Cry* (*Cry1,2*), *Ror* (*Rorα,β,γ*), *Rev-Erb* (*Rev-Erbα,β*) and *Bmal* (*Bmal1,2*). The mRNA and the cytoplasmic/nuclear protein abundances are distinguished for each gene entity and the nuclear shuttling and accumulation were modelled using nuclear import and export rates. Despite the merging of clock elements that belong to the same gene family, their peak phases of expression are within the observed experimental intervals considered for the construction of the mathematical model. This allows for the appropriate assembly of phase differences between the different gene families and as such, for the generation of the necessary delays, needed for the production of a circadian output in gene and protein expression.

The new model adds 26 new ODEs and adjusts 2 ODEs for *Bmal* and *Per* from the CCM (**Table 3**). The number of variables is increased to 46 (**Table 1**) and the number of parameters to 170

(**Table 2**). The missing parameters for the new variables were estimated based on the average values of the previous parameters. We further based our calculations on key biological assumptions relevant for the mammalian circadian oscillator, such as a period of about 23.65 hours and measured phase/amplitude relations between the components of the model, for the wild type scenario.

The model comprises two major compartments, the nucleus (grey) and the cytoplasm (**Figure S1**). There are 20 species included, represented by genes (highlighted in blue boxes), their corresponding cytoplasmic proteins (highlighted in yellow boxes) and cytoplasmic protein complexes (indexed "C") and nuclear proteins and nuclear protein complexes (indexed "N"). The transcriptional activation and phosphorylation/dephosphorylation processes are represented by green lines, transcriptional repressions are represented by red lines. Translation and nuclear importation/exportation processes are represented by black lines while complex formation/dissociation processes are indicated by brown lines. Time units are given in hours and concentration units are given as arbitrary units (a.u.).

In the following section, the model design is explained in detail.

A new circadian model including the cell cycle check point elements Wee1, Myc, Ink4a/Arf

The CLOCK/BMAL complex regulates the expression of several cell cycle checkpoint genes, such as *Wee1* and *Myc* by directly binding to the E-box cis-elements in their promoter region [2, 3]. The binding of CLOCK/BMAL activates the transcription of *Wee1* while it represses *Myc* transcription. Following the design principle of the previously published core-clock model [1], the PER/CRY$_{pool}$ (which includes all possible PER/CRY heterodimers) has an inhibitory effect on the CLOCK/BMAL-mediated transcriptional regulation of target genes (**Figure 1**).

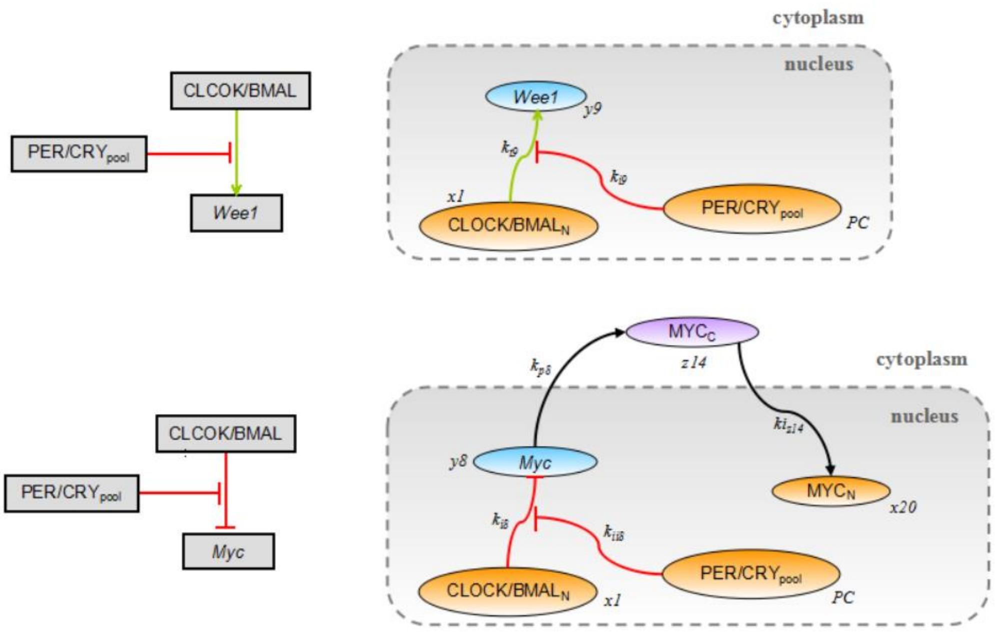

Figure 1: *Wee1* and *Myc* are regulated by the CLOCK/BMAL heterodimer complex. The transcription of *Wee1* and *Myc* is activated and inhibited by the CLOCK/BMAL complex, respectively. These regulations are indirectly repressed by PER/CRY heterodimers. Green arrows represent transcriptional activation; red lines represent transcriptional repression processes; translation and nuclear import processes are represented by black arrows.

The PER proteins, together with the nuclear protein NONO, have been found to activate the transcription of *Ink4a* by binding to its promoter in a circadian manner [4]. As the PER/CRY$_{pool}$ is positively correlated with PER, the activator of *Ink4a*, this series of interactions can be modelled as a positive correlation between the PER/CRY$_{pool}$ and *Ink4a* transcription without losing essential dynamic features of the system. The INK4a protein, which is known as a potent inhibitor of D-type cyclin-dependent kinases, competes for binding to CDK4/6 with CycD and

inhibits the subsequent phosphorylation of RB1 (**Figure 2A**) [5]. In this model, we use CDK to represent all CDKs inhibited by INK4a, namely CDK4 and CDK6.

It has been shown that the expression of ARF, another protein encoded by the CDKN2A locus, can be activated by MYC (**Figure 2B**) [6]. Even though it is not clear if this activation is achieved through a direct binding to the promoter of the *Arf* gene, it is common to model the interaction using Hill-type kinetics [7]. Accumulated ARF stabilizes p53 by binding to MDM2, a E3 ubiquitin ligase targeting p53 in the nucleus (**Figure 2B**) [8].

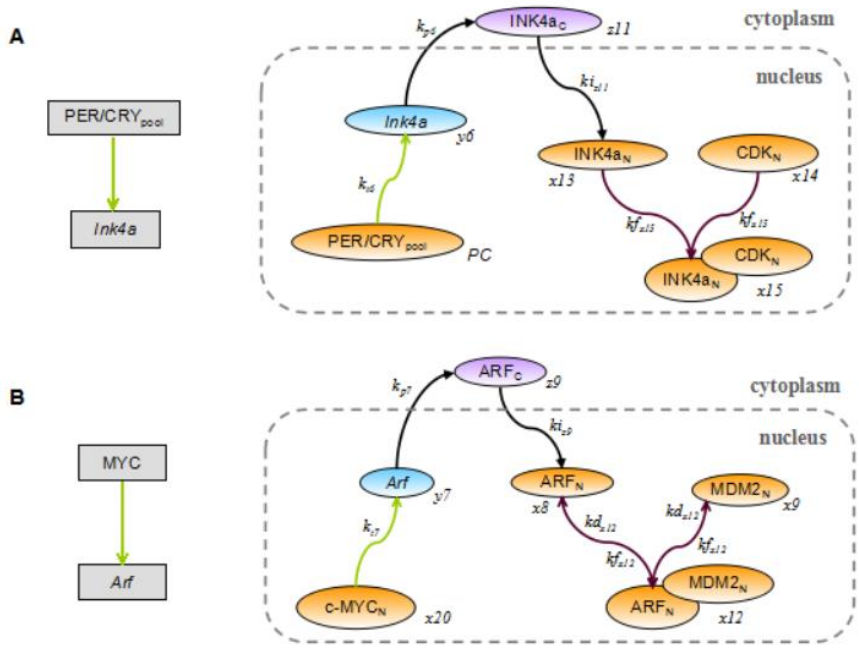

Figure 2: Schematic representation of *Ink4a* and *Arf* reactions considered in the model. Green arrows represent transcriptional activation; brown arrows represent complex formation/dissociation processes; translation and nuclear import processes are represented by black arrows.

The INK4a/RB/E2F pathway and its regulation of Bmal

In order to interpret the circadian phenotype of INK4a/ARF-knockout MEFs, it is necessary to extend the model with a feedback from INK4a and ARF to the core circadian clock. For this, we used the INK4a-CDK/CycD-Rb-E2F pathway (**Figure 3**). The transcription factor MYC directly induces the synthesis of *Cdk4* [9]. CDK4 and another cyclin D-dependent kinase, CDK6, form an active complex with CycD and play an important role in the phosphorylation of RB1, the key regulator of the E2F family of transcription factors. Once RB1 is phosphorylated, active

E2F will be released from the RB1/E2F complex [10-12]. MotifMap, a database of candidate regulatory motif sites in humans, reports that several E2F activators such as E2F1, E2F2, and E2F3a can potentially bind to the promoter of *Bmal1* to activate its transcription [13]. On the other hand, the formation of the CDKs/CycD complex is inhibited by INK4a, which has a negative effect on RB1 phosphorylation and reinforces the inhibition of E2F [5]. MYC also promotes the transcription of the three E2Fs [14, 15]. In this model, we used E2F to represent the three activators belonging to E2F family, i.e. E2F1, E2F2, and E2F3a. The heterodimer MYC:MAX has also been reported to bind to E-boxes and thereby to influence the circadian clock either by inducing REV-ERBα to dampen the expression and oscillation of BMAL1 [16] or by direct repression of BMAL1 and CLOCK via MIZ1 [17]. Moreover, MYC has been reported to repress *Per1* transcriptional activation by CLOCK/BMAL1 via competitive targeting of E-box sequences of the *Per1* promoter [18]. In the model, this connection is included implicitly via the *Bmal* inhibition rate.

In addition, the tumor suppressor protein p53 inhibits the phosphorylation of RB1 via the p21/p27-CDK/CycE-RB1 pathway. Both p21 and p27 are inhibitors of the cyclin E-dependent kinase CDK2, which regulates RB1 phosphorylation and E2F activity synergistically with CDK4/CycD and CDK6/CycD, thus influencing *Bmal* transcription [19, 20]. The transcription of p21 is induced by p53 [21]. To reduce the complexity, the effect of the p53- p21/p27-CDK/CycE arm was modelled as a negative correlation between p53 and the enzymatic activity of CDK/CycE (**Figure 3**).

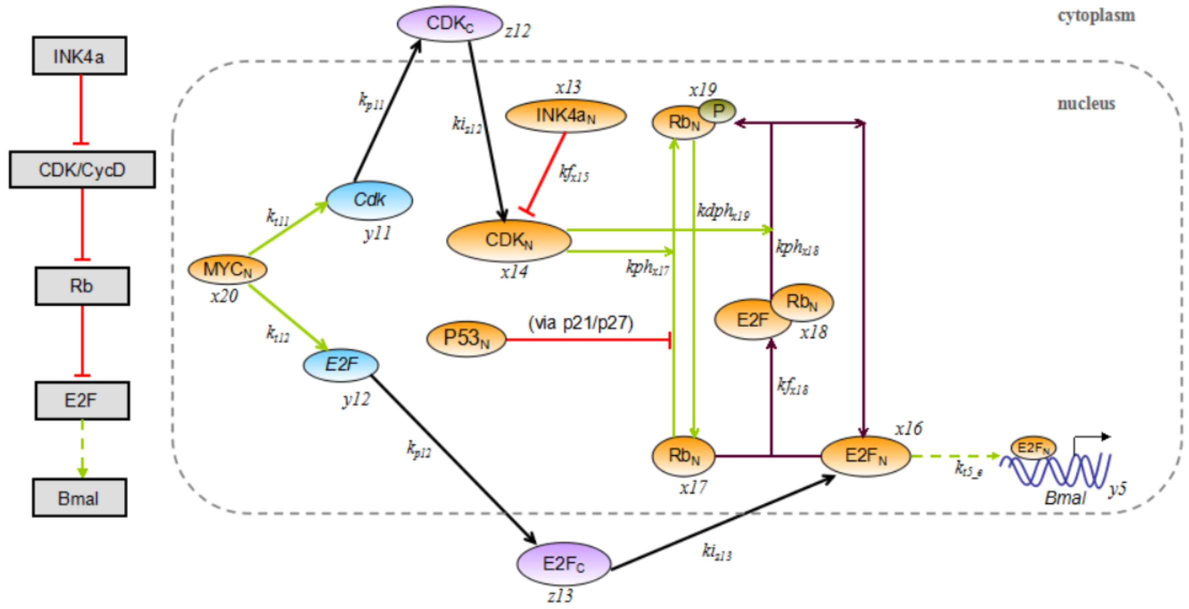

Figure 3: Schematic representation of the INK4a/RB/E2F pathway and its effect on *Bmal* transcription. Green arrows represent transcriptional activation and phosphorylation/ dephosphorylation processes; red lines represent transcriptional repression processes; brown arrows represent complex formation/dissociation processes; translation and nuclear import processes are represented by black arrows.

The ARF/MDM2/p53 pathway and its regulation of Per

The ARF/MDM2/p53/Per pathway is a feedback from ARF to the core circadian clock (**Figure 4**). The expression of ARF can be activated by MYC [6]. Accumulated ARF associates with MDM2 and leads to rapid degradation of MDM2, thereby inhibiting the MDM2-mediated degradation of p53 and promoting p53 stabilisation and accumulation [22]. Recent data showed that there is a p53 response element located in the promoter region of the *Per2* gene which overlaps with E-box cis-elements crucial for CLOCK/BMAL-mediated *Per2* transcription [23]. The binding of p53 strongly represses the transcription of *Per2* by competing with CLOCK/BMAL for binding to the *Per2* promoter [23], as a result p53 and *Per2* are out-of-phase (**Figure 5**).

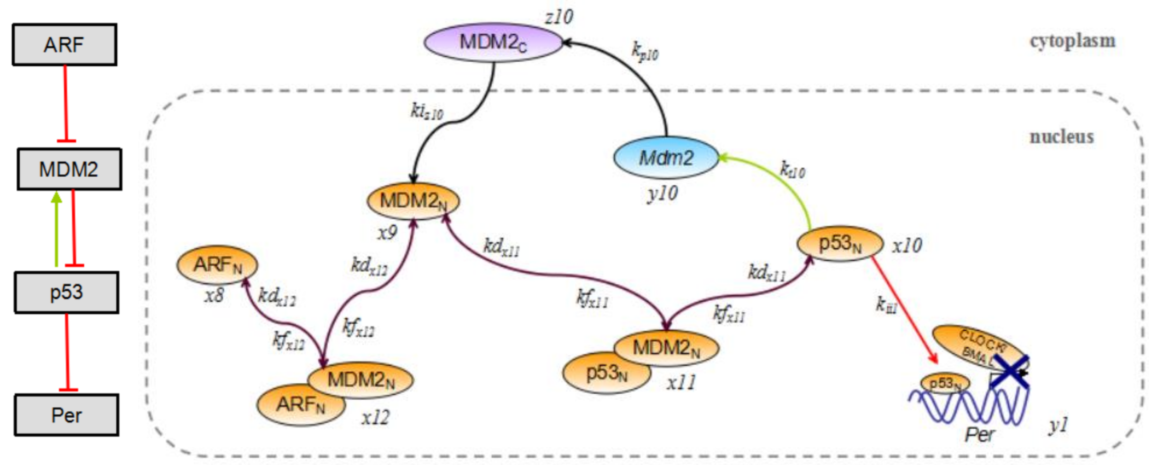

Figure 4: Schematic representation of the ARF/MDM2/p53 pathway and its effect on CLOCK/BMAL-mediated transcription of *Per*. Green arrows represent transcriptional activation; red lines represent transcriptional repression processes; brown arrows represent complex formation/dissociation processes; translation and nuclear import processes are represented by black arrows.

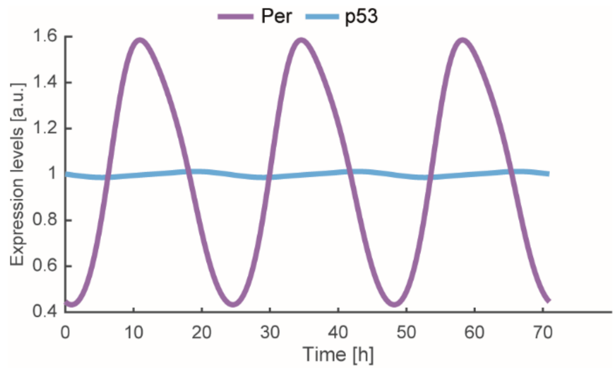

Figure 5: Simulated expression of *Per* and p53. *Per* and p53 show out-of-phase oscillations. The amplitude of p53 is much lower than that of *Per*.

Additional model analysis

To further explore the effect of RAS on the circadian clock *in silico*, we compared the *Bmal* phenotypes and the corresponding changes in period length after the perturbation by different levels of RAS overexpression represented by the parameter *ktt*<1 (**Figure 6**). When measuring the period for the first six peaks (five periods) after introducing the perturbation of RAS (represented by *ktt*<1), the same trend could be observed as for measuring the first three periods (**Figure 7**). Furthermore, we simulated the *Bmal* phenotype of the Ink4a/Arf$^{-/-}$ system following an inhibition of RAS (represented by *ktt*=1.2) which resulted in a longer period (**Figure 8**) as was also observed in our experimental data (**Figure S1C,E**).

We additionally investigated the importance of the INK4a/RB1/E2F1 pathway (module 1) and the ARF/MDM2/p53 pathway (module 2) in reproducing the effect of RAS overexpression on the *Bmal* period by either uncoupling them from the core-clock system or by setting their expression to their constitutive average value (**Figure 9**).

In the model, we measured the period in the transient region of the simulations. This is in agreement with our RT-qPCR data in IMR-90 cells on day 5 and 11 after overexpression of RAS. The data show that despite the assumed stability of retrovirus-mediated *Hras* overexpression, the expression level of *Hras* display some biological noise: it first strongly increases (day 5) and then decreases again (**Figure 10**).

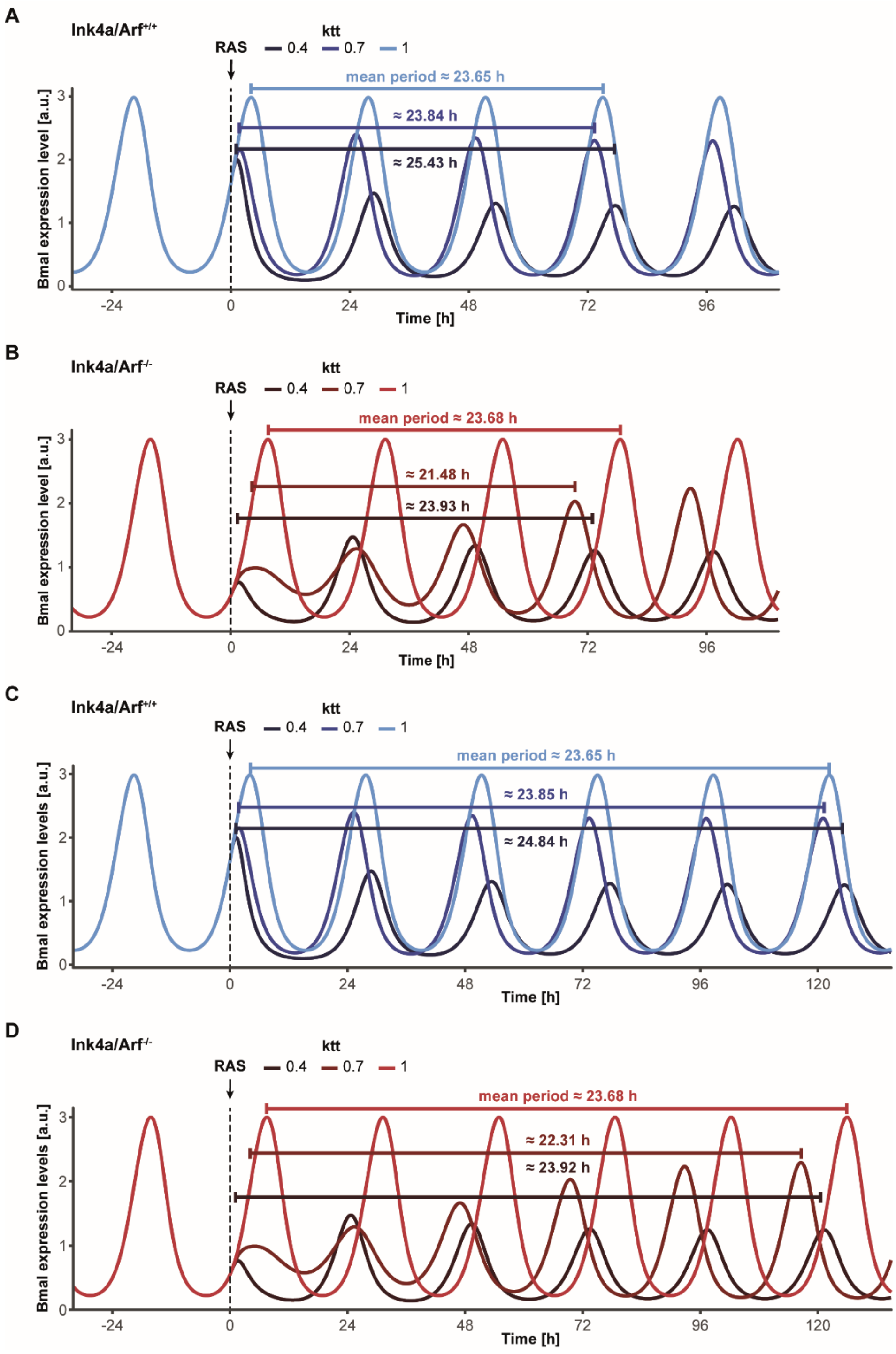

Figure 6: *In silico Bmal* phenotypes after perturbation by different levels of RAS. The period was measured for a transient region, defined as the mean of the time between the first four peaks (three periods) after introducing the perturbation of RAS (represented by *ktt*<1) for **(A)** the Ink4a/Arf$^{+/+}$ system and **(B)** the Ink4a$^{-/-}$ system. When measuring the first five periods instead, we still see the same tendency of period changes in dependency of ktt for **(C)** the Ink4a/Arf$^{+/+}$ system and **(D)** the Ink4a$^{-/-}$ system.

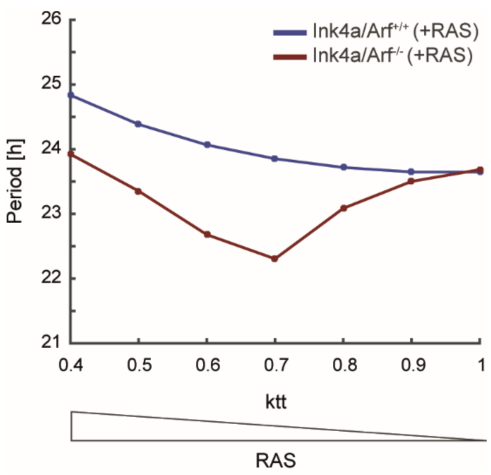

Figure 7: The model qualitatively reproduces experimental period changes upon RAS overexpression. *In silico* expression data show that upon simulation of RAS overexpression, the Ink4a/Arf$^{+/+}$ system acquires a longer and Ink4a/Arf$^{-/-}$ system a shorter period compared to the corresponding simulated wild type system. The period was measured for a transient region, defined as the mean of the time between the first six peaks (five periods) after introducing the perturbation of RAS (represented by *ktt*<1).

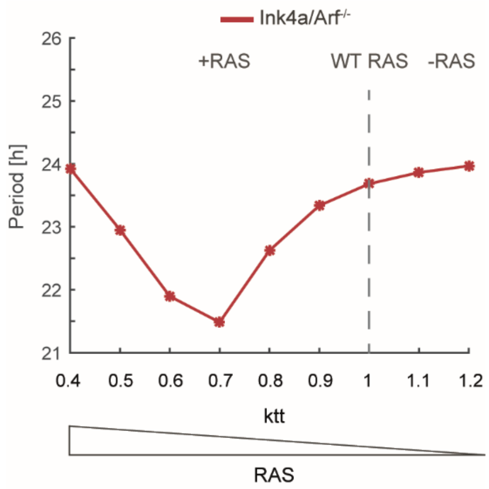

Figure 8: The model predicts an increase in period length upon RAS inhibition. *In silico* expression data show that upon simulation of RAS inhibition (-RAS), the Ink4a/Arf$^{-/-}$ system acquires a longer period compared to the corresponding system with WT RAS (*ktt*=1). The period was measured for a transient region, defined as the mean of the time between the first four peaks (three periods) after introducing the perturbation of RAS (overexpression represented by *ktt*<1 and inhibition represented by *ktt*>1).

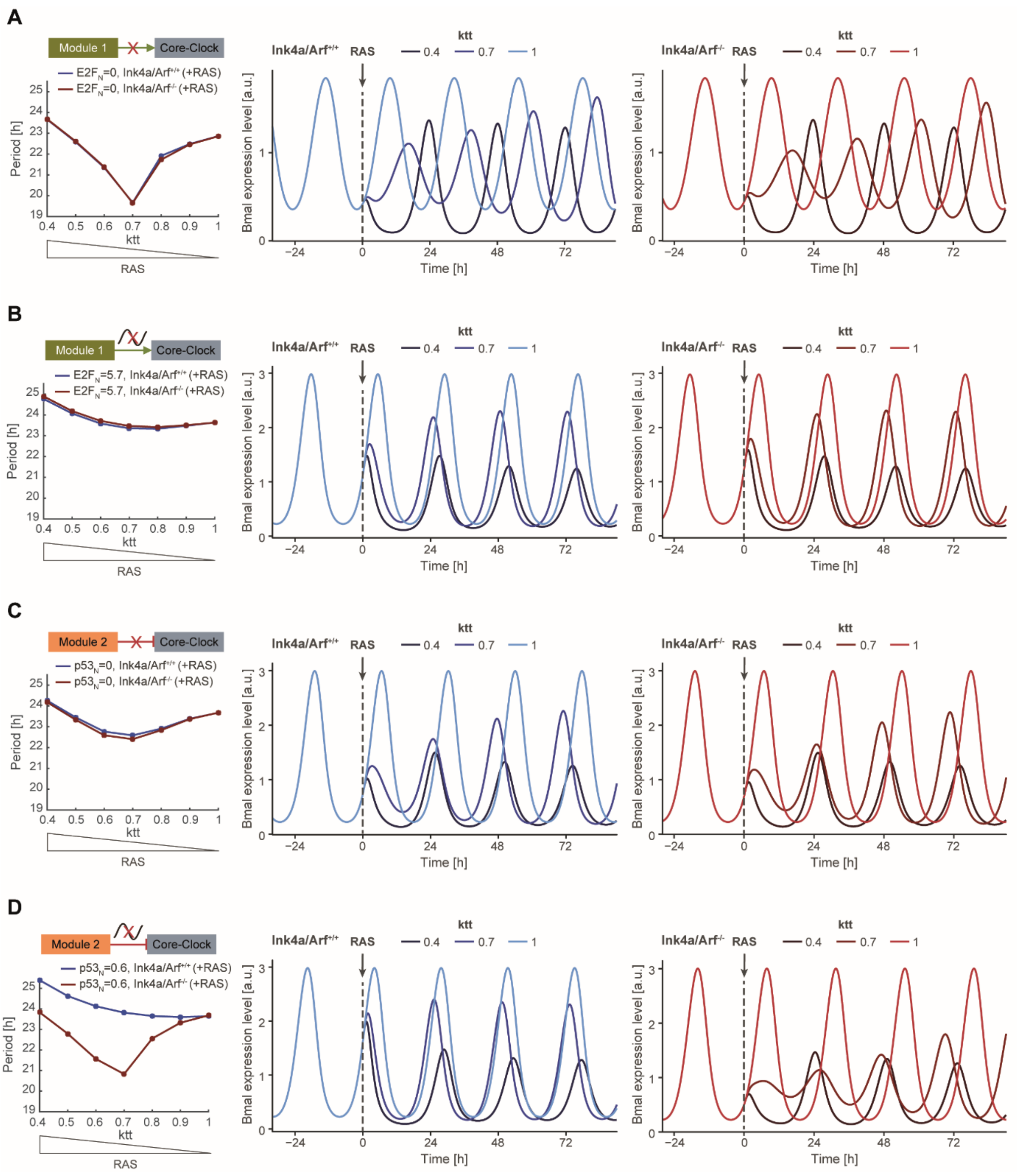

Figure 9: Modular analysis of *Bmal* expression level after perturbation by different levels of RAS. The importance of the INK4a/RB1/E2F1 pathway (module 1) and the ARF/MDM2/p53 pathway (module 2) in influencing the circadian period is analysed by simulating different scenarios *in silico*. The simulated *Bmal* expression profiles show phase-shifted oscillations that cause differing effects following the perturbation by RAS (represented by *ktt*<1). A) Module 1 is decoupled from the core-clock or B) the oscillatory expression of its connective component $E2F_N$ is clamped to its constitutive average value. C) Module 2 is decoupled from the core-clock or D) the oscillatory expression of its connective component $p53_N$ is clamped to its constitutive average value.

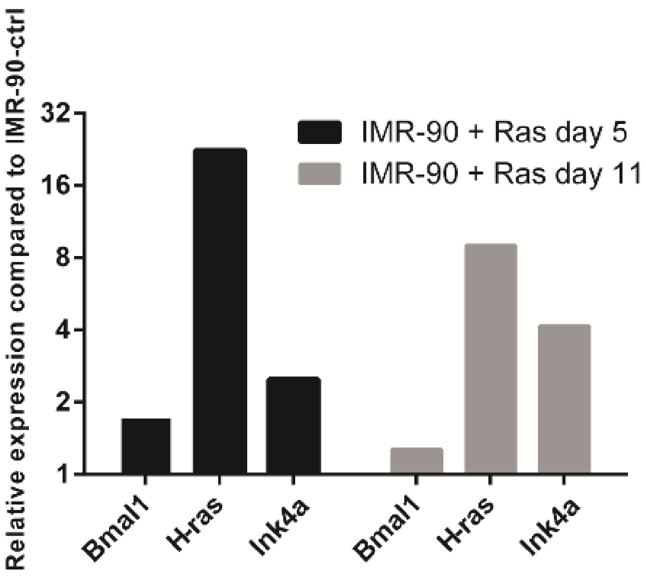

Figure 10: Time-dependent change of gene levels after Hras overexpression in IMR-90 cells. RT-qPCR data show that while *Bmal1* and *Ink4/Arf* are upregulated after retrovirus-mediated *Hras* overexpression in IMR-90 cells, their expression levels change over the course of the next 11 days, as does the expression of *Hras* itself. Numerical values are provided in S1 Data.

Table 1: List of variables. *- Phosphorylated proteins, "c"-indexed - cytoplasmic proteins, "N"-indexed - nuclear proteins.

Variable [a.u.]	Name	Note
x1	CLOCK/BMAL	CCM
x2	PER^*_N/CRY_N	CCM
x3	PER_N/CRY_N	CCM
PC	PER/CRY_{pool}	CCM
x5	$REV\text{-}ERB_N$	CCM
x6	ROR_N	CCM
x7	$BMAL_N$	CCM
x8	ARF_N	CCRM
x9	$MDM2_N$	CCRM
x10	$p53_N$	CCRM
x11	$p53/MDM2_N$	CCRM
x12	$ARF/MDM2_N$	CCRM
x13	$INK4a_N$	CCRM
x14	$CDK/CycD_N$	CCRM
x15	$CDK/CycD/INK4a_N$	CCRM
x16	$E2F_N$	CCRM
x17	RB_N	CCRM
x18	$RB\text{-}E2F_N$	CCRM
x19	RB^*_N	CCRM
x20	MYC_N	CCRM
y1	*Per*	CCM
y2	*Cry*	CCM
y3	*Rev-Erb*	CCM
y4	*Ror*	CCM

Variable [a.u.]	Name	Note
y5	*Bmal*	CCM
y6	*Ink4a*	CCRM
y7	*Arf*	CCRM
y8	*Myc*	CCRM
y9	*Wee1*	CCRM
y10	*Mdm2*	CCRM
y11	*CDK/CycD*	CCRM
y12	*E2f*	CCRM
z1	CRY_c	CCM
z2	PER_c	CCM
z3	PER^*_c	CCM
z4	PER^*_c/CRY_c	CCM
z5	PER_c/CRY_c	CCM
z6	$REV\text{-}ERB_c$	CCM
z7	ROR_c	CCM
z8	$BMAL_c$	CCM
z9	ARF_c	CCRM
z10	$MDM2_c$	CCRM
z11	$INK4a_c$	CCRM
z12	$CDK/CycD_c$	CCRM
z13	$E2F_c$	CCRM
z14	MYC_c	CCRM

Table 2: List of parameters. [a]Average value of all parameters in the same category used in [1]. [b]The hill coefficients of new components was pre-set to 1 at this stage. [c]Parameters which were fine-tuned to maintain the oscillations of the system and to fit experimental observations.

Parameters	Name	Value	Reference
**Degradation rates for nuclear proteins or nuclear protein complexes [hour^{-1}]**			
dx1	CLOCK/BMAL	0.08	[1]
dx2	$PER*_N/CRY_N$	0.06	[1]
dx3	PER_N/CRY_N	0.09	[1]
dx5	REV-ERB_N	0.17	[1]
dx6	ROR_N	0.12	[1]
dx7	$BMAL_N$	0.15	[1]
dx8	ARF_N	0.11	[24]
dx9	$MDM2_N$	0.46	[22]
dx10	$p53_N$	0.231	[22]
dx11	$p53/MDM2_N$	2.07	[25]
dx12	$ARF/MDM2_N$	1.39	[22]
dx13	$INK4a_N$	0.11	[26]
dx14	$CDK/CycD_N$	1.5	[27, 28]
dx16	$E2F_N$	0.35	[29]
dx17	RB_N	0.069	[30]
dx18	RB-$E2F_N$	0.03	[31, 32]
dx19	$RB*_N$	0.069	[32, 33]
dx20	MYC_N	1.39	[34, 35]
**Degradation rates for mRNAs [hour^{-1}]**			
dy1	*Per*	0.3	[36]
dy2	*Cry*	0.2	[1]
dy3	*Rev-Erb*	2	[1]

dy4	*Ror*	0.2	[1]
dy5	*Bmal*	1.6	[1]
dy6	*Ink4a*	0.86 [a]	
dy7	*Arf*	0.69	
dy8	*Myc*	0.86 [a]	
dy9	*Wee1*	0.86 [a]	
dy10	*Mdm2*	0.36	[37]
dy11	*CDK/CycD*	0.86 [a]	
dy12	*E2f*	0.25	
	**Degradation rates for cytoplasmic proteins [hour^{-1}]**		
dz1	CRY_c	0.23	[1]
dz2	PER_c	0.25	[1]
dz3	PER^*_c	0.6	[1]
dz4	PER^*_c/CRY_c	0.2	[1]
dz5	PER_c/CRY_c	0.2	[1]
dz6	$REV\text{-}ERB_c$	0.31	[1]
dz7	ROR_c	0.3	[1]
dz8	$BMAL_c$	0.73	[1]
dz9	ARF_c	0.3525 [a]	
dz10	$MDM2_c$	0.3525 [a]	
dz11	$INK4a_c$	0.3525 [a]	
dz12	$CDK/CycD_c$	0.7	
dz13	$E2F_c$	0.7	
dz14	MYC_c	0.7	[14]
	**Reaction rates for complex formation/dissociation**		
kfx1	CLOCK/BMAL-complex formation	2.3	[1]

kdx1	CLOCK/BMAL-complex dissociation	0.01	[1]
kfz4	PER^{*}_{c}/CRY_{c}-complex formation	1	[1]
kdz4	PER^{*}_{c}/CRY_{c}-complex dissociation	1	[1]
kfz5	PER_{c}/CRY_{c}-complex formation	1	[1]
kdz5	PER_{c}/CRY_{c}-complex dissociation	1	[1]
kfx11	$P53/MDM2_{N}$-complex formation	3.96	
kdx11	$P53/MDM2_{N}$-complex dissociation	0.0396	
kfx12	$ARF/MDM2_{N}$-complex formation	8	
kdx12	$ARF/MDM2_{N}$-complex dissociation	0.0396	
kfx15	$INK4a/CDK/CYCD_{N}$-complex formation	8	
kfx18	RB/E2F-complex formation	18	
	**Phosphorylation/dephosphorylation reaction rates [$hour^{-1}$]**		
kphz2	PER_{C} phosphorylation rate	2	[1]
kdphz3	PER_{C}* dephosphorylation rate	0.05	[1]
kphx17	RB phosphorylation rate	18	[32]
kdphx19	RB* dephosphorylation rate	3.6	[32]
Kph	activation constant for RB phosphorylation by CDK/CycD	0.92	[38]
Kdph	activation constant for RB^{*} dephosphorylation	0.01	[39]
Kbp	inhibition constant for RB phosphorylation by p53	0.2 [c]	
	**Transcription rates [a.u. $hour^{-1}$]**		
V_{1max}	*Per*	1	[1]
V_{2max}	*Cry*	2.92	[1]
V_{3max}	*Rev-Erb*	1.9	[1]
V_{4max}	*Ror*	10.9	[1]
V_{5max}	*Bmal*	1	[1]
V_{6max}	*Ink4a*	3.544 [a]	

V_{7max}	*Arf*	3.544 [a]	
V_{8max}	*Myc*	3.544 [a]	
V_{9max}	*Wee1*	3.544 [a]	
V_{10max}	*Mdm2*	5.4	[40]
V_{11max}	*Cdk/CycD*	3.544 [a]	
V_{12max}	*E2f*	3.544 [a]	
	**Activation/inhibition rates**		
kt1	*Per* activation rate	3	[1]
ki1	*Per* inhibition rate	0.9	[1]
kt2	*Cry* activation rate	2.4	[1]
ki2	*Cry* inhibition rate (by PER/CRY_{pool})	0.7	[1]
ki21	*Cry* inhibition rate (by $REV\text{-}ERB_N$)	5.2	[1]
kt3	*Rev-Erb* activation rate	2.07	[1]
ki3	*Rev-Erb* inhibition rate	3.3	[1]
kt4	*Ror* activation rate	0.9	[1]
ki4	*Ror* inhibition rate	0.4	[1]
kt5	*Bmal* activation rate	8.35	[1]
ki5	*Bmal* inhibition rate	1.94	[1]
kii1	*Per* inhibition rate 2 (by p53)	2.488 [a]	
kt5_e	*Bmal* activation rate (by E2F)	5 [c]	
kt6	*Ink4a* activation rate	3.344 [a]	
kt7	*Arf* activation rate	3.344 [a]	
ki8	*Myc* inhibition rate 1	2.488 [a]	
kii8	*Myc* inhibition rate 2 (PC to CB)	2.488 [a]	
kt9	*Wee1* activation rate	3.344 [a]	
ki9	*Wee1* inhibition rate	2.488 [a]	

kt10	*Mdm2* activation rate	1.85	[40]
kt11	*Cdk* activation rate	0.15 [c]	
kt12	*E2f* activation rate	3.344 [a]	
	**Transcription fold activation (dimensionless)**		
a	*Per*	12	[1]
d	*Cry*	12	[1]
g	*Rev-Erb*	5	[1]
h	*Ror*	5	[1]
i	*Bmal*	12	[1]
a_1	*Bmal* (by E2F)	3 [c]	
o	*Ink4a*	9.2 [a]	
l	*Arf*	9.2 [a]	
l1	*Wee1*	9.2 [a]	
r1	*Mdm2*	11	[40]
r2	*Cdk4*	9.2 [a]	
r3	*E2f*	9.2 [a]	
	**Production rates [hour^{-1}]**		
kp1	PER_c	0.4	[1]
kp2	CRY_c	0.26	[1]
kp3	$REV\text{-}ERB_c$	0.37	[1]
kp4	ROR_c	0.76	[1]
kp5	$BMAL_c$	1.21	[1]
kp6	$INK4a_c$	0.6 [a]	
kp7	ARF_c	0.6 [a]	
kp8	MYC_c	0.6 [a]	
kp10	$MDM2_c$	0.6 [a]	

kp11	CDK_c	0.6 [a]	
kp12	$E2F_c$	0.4	
**Import/Export rates [hour^{-1}]**			
kiz4	PER^*/CRY_c	0.2	[1]
kiz5	PER/CRY_c	0.1	[1]
kiz6	$REV\text{-}ERB_c$	0.5	[1]
kiz7	ROR_c	0.1	[1]
kiz8	$BMAL_c$	0.1	[1]
kex2	PER^*/CRY_N	0.02	[1]
kex3	PER/CRY_N	0.02	[1]
kiz10	$MDM2_c$	0.2 [a]	
kiz11	$INK4a_c$	0.2 [a]	
kiz9	ARF_c	0.2 [a]	
kiz12	CDK_c	0.2 [a]	
kiz13	$E2F_c$	0.2 [a]	
kiz14	MYC_c	0.2 [a]	
**Hill coefficients of transcription (dimensionless)**			
b	*Per* activation	5	[1]
c	*Per* inhibition	7	[1]
e	*Cry* activation	6	[1]
f	*Cry* inhibition	4	[1]
f1	*Cry* inhibition	1	[1]
v	*Rev-Erb* activation	6	[1]
w	*Rev-Erb* inhibition	2	[1]
p	*Ror* activation	6	[1]
q	*Ror* inhibition	3	[1]

n	*Bmal* activation	2	[1]
m	*Bmal* inhibition	5	[1]
r	*Ink4a* activation	1 [b]	
s	*Arf* activation	1 [b]	
h4	*Myc* inhibition 1	1 [b]	
h5	*Myc* inhibition 2	1 [b]	
h6	*Wee1* activation	1 [b]	
h7	*Wee1* inhibition	1 [b]	
h1	*Mdm2* activation	1.8	[41]
h8	*Per* inhibition (by p53)	1 [b]	
a_2	*Bmal* (by E2F)	1 [b]	
h2	*Cdk* activation	1 [b]	
h3	*E2F* activation	1 [b]	
	**Exogenous RNA [a.u.]**		
$y1_0$	*Per*	0	[1]
$y2_0$	*Cry*	0	[1]
$y3_0$	*Rev-Erb*	0	[1]
$y4_0$	*Ror*	0	[1]
$y5_0$	*Bmal*	0	[1]
Ink4a0	*Ink4a*	0 [a]	
Mdm0	*Mdm2*	0 [a]	
Arf0	*Arf*	0 [a]	
CDK0	*Cdk*	0 [a]	
Myc0	*Myc*	0 [a]	
E2F0	*E2f*	0 [a]	

	**Nuclear protein [a.u.]**	
source_p53	p53	4.5 [c]
source_RB	RB	1 [c]
	**Weight factors [a.u.]**	
a2	PER*/CRY_N	1 [c]
a3	PER/CRY_N	1 [c]

Table 3: Equations of the circadian cell cycle model.

	ODEs	
Ink4a	$$\frac{dy6}{dt} = (1 + ln\frac{1}{ktt})V_{6max}\frac{1 + o\left(\frac{PC}{k_{t6}}\right)^r}{1 + \left(\frac{PC}{k_{t6}}\right)^r} - d_{y6}y6$$	**(1)**
Arf	$$\frac{dy7}{dt} = V_{7max}\frac{1 + l\left(\frac{x20}{k_{t7}}\right)^s}{1 + \left(\frac{x20}{k_{t7}}\right)^s} - d_{y7}y7$$	**(2)**
Myc	$$\frac{dy8}{dt} = V_{8max}\frac{1}{1 + \frac{k_{ii8}^{h5}}{k_{ii8}^{h5} + PC^{h5}}\left(\frac{x1}{ktt \cdot k_{i8}}\right)^{h4}} - d_{y8}y8$$	**(3)**
Wee1	$$\frac{dy9}{dt} = (1 + ln\frac{1}{ktt})V_{9max}\frac{1 + l1\left(\frac{x1}{ktt \cdot k_{t9}}\right)^{h6}}{1 + \left(\frac{x1}{ktt \cdot k_{t9}}\right)^{h6} + \left(\frac{PC}{k_{i9}}\right)^{h7}\left(\frac{x1}{ktt \cdot k_{t9}}\right)^{h6}} - d_{y9}y9$$	**(4)**
Mdm2	$$\frac{dy10}{dt} = V_{10max}\frac{1 + r1\left(\frac{x10}{k_{t10}}\right)^{h1}}{1 + \left(\frac{x10}{k_{t10}}\right)^{h1}} - d_{y10}y10$$	**(5)**
CDK/CycD	$$\frac{dy11}{dt} = V_{11max}\frac{1 + r2\left(\frac{x20}{k_{t11}}\right)^{h2}}{1 + \left(\frac{x20}{k_{t11}}\right)^{h2}} - d_{y11}y11$$	**(6)**
E2f	$$\frac{dy12}{dt} = V_{12max}\frac{1 + r3\left(\frac{x20}{k_{t12}}\right)^{h3}}{1 + \left(\frac{x20}{k_{t12}}\right)^{h3}} - d_{y12}y12$$	**(7)**
**ARF$_c$**	$$\frac{dz9}{dt} = k_{p7}(y7 + y7_0) - ki_{z9}z9 - d_{z9}z9$$	**(8)**
**MDM2$_c$**	$$\frac{dz10}{dt} = k_{p10}(y10 + y10_0) - ki_{z10}z10 - d_{z10}z10$$	**(9)**
**INK4a$_c$**	$$\frac{dz11}{dt} = k_{p6}(y6 + y6_0) - ki_{z11}z11 - d_{z11}z11$$	**(10)**
**CDK/CycD$_c$**	$$\frac{dz12}{dt} = k_{p11}(y11 + y11_0) - ki_{z12}z12 - d_{z12}z12$$	**(11)**
**E2F$_c$**	$$\frac{dz13}{dt} = k_{p12}(y12 + y12_0) - ki_{z13}z13 - d_{z13}z13$$	**(12)**

**MYC_c**	$$\frac{dz14}{dt} = k_{p8}(y8 + y8_0) - ki_{z14}z14 - d_{z14}z14$$	**(13)**
**ARF_N**	$$\frac{dx8}{dt} = ki_{z9}z9 + kd_{x12}x12 - kf_{x12}x8x9 - d_{x8}x8$$	**(14)**
**$MDM2_N$**	$$\frac{dx9}{dt} = ki_{z10}z10 + kd_{x11}x11 + kd_{x12}x12 - kf_{x11}x9x10 - kf_{x12}x8x9 - d_{x9}x9$$	**(15)**
**$P53_N$**	$$\frac{dx10}{dt} = source\_p53 + kd_{x11}x11 - kf_{x11}x9x10 - d_{x10}x10$$	**(16)**
**$MDM2/p53_N$**	$$\frac{dx11}{dt} = kf_{x11}x9x10 - kd_{x11}x11 - d_{x11}x11$$	**(17)**
**$ARF/MDM2_N$**	$$\frac{dx12}{dt} = kf_{x12}x8x9 - kd_{x12}x12 - d_{x12}x12$$	**(18)**
**$INK4a_N$**	$$\frac{dx13}{dt} = ki_{z11}z11 - kf_{x15}x13x14 - d_{x13}x13$$	**(19)**
**$CDK/CycD_N$**	$$\frac{dx14}{dt} = ki_{z12}z12 - kf_{x15}x13x14 - d_{x14}x14$$	**(20)**
**$CDK/CycD/INK4_N$**	$$\frac{dx15}{dt} = kd_{x15}x15x14 - d_{x15}x15$$	**(21)**
**$E2F_N$**	$$\frac{dx16}{dt} = ki_{z13}z13 - kph_{x17}\left(x14 + \frac{Kbp}{Kbp + x10}\right)\frac{x18}{x18 + Kph} - kf_{x18}x16x17 - d_{x16}x16$$	**(22)**
**RB_N**	$$\frac{dx17}{dt} = source_{Rb} + kdph_{x19}\frac{x19}{x19 + Kdph} - kph_{x17}\left(x14 + \frac{Kbp}{Kbp + x10}\right)\frac{x17}{x17 + Kph} - kf_{x18}x16x17 - d_{x17}x17$$	**(23)**
**$RB/E2F_N$**	$$\frac{dx18}{dt} = kf_{x18}x16x17 - kph_{x17}\left(x14 + \frac{Kbp}{Kbp + x10}\right)\frac{x18}{x18 + Kph} - d_{x18}x18$$	**(24)**
**RB_N***	$$\frac{dx19}{dt} = kph_{x17}\left(x14 + \frac{Kbp}{Kbp + x10}\right)\left(\frac{x17}{x17 + Kph} + \frac{x18}{x18 + Kph}\right) - kdph_{x19}\frac{x19}{x19 + Kdph} - d_{x19}x19$$	**(25)**
**MYC_N**	$$\frac{dx20}{dt} = ki_{z14}z14 - d_{x20}x20$$	**(26)**
Bmal	$$\frac{dy5}{dt} = V_{5max}\frac{1 + i\left(\frac{x6}{k_{t5}}\right)^n}{1 + \left(\frac{x5}{k_{i5}}\right)^m + \left(\frac{x6}{k_{t5}}\right)^n}\frac{1 + a\_1\left(\frac{x16}{k_{t5\_e}}\right)^{a\_2}}{1 + \left(\frac{x16}{k_{t5\_e}}\right)^{a\_2}} - d_{y5}y5$$	**(27)**

Per	$$\frac{dy1}{dt} = V_{1max}\frac{1 + a\left(\frac{x1}{ktt \cdot k_{t1}}\right)^{b}}{1 + \left(\frac{PC}{k_{i1}}\right)^{c}\left(\frac{x1}{ktt \cdot k_{t1}}\right)^{b} + \left(\frac{x1}{ktt \cdot k_{t1}}\right)^{b} + \left(\frac{x1}{ktt \cdot k_{t1}}\right)^{b}\left(\frac{x10}{k_{ii1}}\right)^{h8}} - d_{y1}y1 \quad (28)$$
**CLOCK/BMAL**	$$\frac{dx1}{dt} = kf_{x1}x7 - kd_{x1}x1 - d_{x1}x1 \quad (29)$$
Rev-Erb	$$\frac{dy3}{dt} = V_{3max}\frac{1 + g\left(\frac{x1}{ktt \cdot k_{t3}}\right)^{v}}{1 + \left(\frac{PC}{k_{i3}}\right)^{w}\left(\frac{x1}{ktt \cdot k_{t3}}\right)^{v} + \left(\frac{x1}{ktt \cdot k_{t3}}\right)^{v}} - d_{y3}y3 \quad (30)$$
Ror	$$\frac{dy4}{dt} = V_{4max}\frac{1 + h\left(\frac{x1}{ktt \cdot k_{t4}}\right)^{p}}{1 + \left(\frac{PC}{k_{i4}}\right)^{q}\left(\frac{x1}{ktt \cdot k_{t4}}\right)^{p} + \left(\frac{x1}{ktt \cdot k_{t4}}\right)^{p}} - d_{y4}y4 \quad (31)$$
**REV-ERB$_C$**	$$\frac{dz6}{dt} = k_{p3}(y3 + y3_0) - ki_{z6}z6 - d_{z6}z6 \quad (32)$$
**ROR$_C$**	$$\frac{dz7}{dt} = k_{p4}(y4 + y4_0) - ki_{z7}z7 - d_{z7}z7 \quad (33)$$
**REV-ERB$_N$**	$$\frac{dx5}{dt} = ki_{z6}z6 - d_{x5}x5 \quad (34)$$
**ROR$_N$**	$$\frac{dx6}{dt} = ki_{z7}z7 - d_{x6}x6 \quad (35)$$
**BMAL$_C$**	$$\frac{dz8}{dt} = k_{p5}(y5 + y5_0) - ki_{z8}z8 - d_{z8}z8 \quad (36)$$
**BMAL$_N$**	$$\frac{dx7}{dt} = ki_{z8}z8 + kd_{x1}x1 - kf_{x1}x7 - d_{x7}x7 \quad (37)$$
Cry	$$\frac{dy2}{dt} = V_{2max}\frac{1 + d\left(\frac{x1}{ktt \cdot k_{t2}}\right)^{e}}{1 + \left(\frac{PC}{k_{i2}}\right)^{f}\left(\frac{x1}{ktt \cdot k_{t2}}\right)^{e} + \left(\frac{x1}{ktt \cdot k_{t2}}\right)^{e}}\frac{1}{1 + (\frac{x5}{k_{i21}})^{f1}} - d_{y2}y2 \quad (38)$$
**CRY$_C$**	$$\frac{dz1}{dt} = k_{p2}(y2 + y2_0) + kd_{z4}z4 + kd_{z5}z5 - kf_{z5}z1z2 - kf_{z4}z1z3 - d_{z1}z1 \quad (39)$$
**PER$_C$**	$$\frac{dz2}{dt} = k_{p1}(y1 + y1_0) + kd_{z5}z5 + kd_{phz3}z3 - kf_{z5}z2z1 - kph_{z2}z2 - d_{z2}z2 \quad (40)$$
**PER$_C^*$**	$$\frac{dz3}{dt} = kph_{z2}z2 + kd_{z4}z4 - kd_{phz3}z3 + kf_{z4}z3z1 - d_{z3}z3 \quad (41)$$
**PER*/CRY$_C$**	$$\frac{dz3}{dt} = kf_{z4}z1z3 + ke_{x2}x2 - ki_{z4}z4 + kd_{z4}z4 - d_{z4}z4 \quad (42)$$
**PER/CRY$_C$**	$$\frac{dz5}{dt} = kf_{z5}z1z2 + ke_{x3}x3 - ki_{z5}z5 + kd_{z5}z5 - d_{z5}z5 \quad (43)$$

**PER*/CRY$_N$**	$\frac{dx2}{dt} = ki_{z4}z4 - ke_{x2}x2 - d_{x2}x2$	**(44)**
**PER/CRY$_N$**	$\frac{dx3}{dt} = ki_{z5}z5 - ke_{x3}x3 - d_{x3}x3$	**(45)**
	**non-ODEs**	
**PER/CRY$_{pool}$**	$PC = x2 + x3$	**(46)**

Table 4: Robustness analysis of the model parameters. The robustness analysis was conducted to investigate how minor changes in the parameter values effect on the overall system. The parameter values were both decreased and increased by 10% and the subsequent variation of the overall system period compared to the wild type period. -10%: 10% decrease in the parameter value; +10%: 10% increase in the parameter value; T_{new}: new value for T after the perturbation; DT%: variation of the new period value to the wild type value. The wild-type period is 23.65 h.

Parameter	-10%		+10%	
	T_{new}	DT%	T_{new}	DT%
dx1	23.89	1.019	23.49	-0.693
dx2	23.85	0.846	23.52	-0.554
dx3	23.7	0.224	23.62	-0.144
dx5	24.04	1.653	23.19	-1.953
dx6	23.82	0.723	23.49	-0.698
dx7	23.66	0.059	23.64	-0.059
dx8	23.65	0	23.65	0
dx9	23.65	0.004	23.65	-0.004
dx10	23.65	0	23.65	0
dx11	23.65	0	23.65	0
dx12	23.65	0	23.65	0
dx13	23.65	-0.008	23.65	0.008
dx14	23.65	0	23.65	0
dx16	23.67	0.068	23.63	-0.068
dx17	23.65	0	23.65	0
dx18	23.65	0	23.65	0
dx19	23.65	0	23.65	0
dx20	23.67	0.072	23.64	-0.063
dy1	23.78	0.529	23.62	-0.14
dy2	23.66	0.03	23.65	-0.021
dy3	23.95	1.277	23.36	-1.209
dy4	23.82	0.706	23.49	-0.685
dy5	23.94	1.222	23.42	-0.989
dy6	23.64	-0.051	23.67	0.08
dy7	23.65	-0.004	23.65	0.004
dy8	23.67	0.072	23.64	-0.063
dy9	23.65	0	23.65	0
dy10	23.66	0.021	23.65	-0.021
dy11	23.67	0.093	23.64	-0.047
dy12	23.67	0.076	23.63	-0.08

Parameter	-10%		+10%	
	T_{new}	DT%	T_{new}	DT%
dz1	23.66	0.0381	23.64	-0.038
dz2	23.65	-0.0085	23.65	0.008
dz3	23.67	0.0719	23.64	-0.051
dz4	23.68	0.1184	23.63	-0.076
dz5	23.65	-0.0085	23.65	0.013
dz6	23.82	0.7019	23.48	-0.702
dz7	23.75	0.4144	23.55	-0.406
dz8	24.04	1.649	23.33	-1.336
dz9	23.65	-0.004	23.65	0.004
dz10	23.65	0.013	23.65	-0.013
dz11	23.64	-0.047	23.66	0.059
dz12	23.67	0.076	23.64	-0.047
dz13	23.67	0.068	23.63	-0.068
dz14	23.66	0.055	23.64	-0.051
kfx1	23.77	0.5116	23.55	-0.427
kdx1	23.65	0.0085	23.65	-0.008
kfz4	23.65	-0.0211	23.66	0.021
kdz4	23.66	0.0381	23.64	-0.034
kfz5	23.65	0.0169	23.65	-0.017
kdz5	23.65	-0.0169	23.65	0.017
kfx11	23.65	-0.004	23.65	0
kdx11	23.65	0	23.65	0
kfx12	23.65	0	23.65	0
kdx12	23.65	0	23.65	0
kfx15	23.65	0.013	23.65	-0.013
kfx18	23.65	0.004	23.65	-0.004
kphz2	23.65	0.0042	23.65	-0.004
kdphz3	23.65	0	23.65	0
kphx17	23.63	-0.068	23.66	0.038
kdphx19	23.66	0.03	23.64	-0.042
Kph	23.65	0.017	23.65	-0.017
Kdph	23.65	0	23.65	0
Kbp	23.64	-0.03	23.66	0.021
V1max	23.7	0.224	23.6	-0.199
V2max	23.67	0.093	23.64	-0.063
V3max	23.48	-0.727	23.81	0.672
V4max	23.6	-0.199	23.68	0.131

Parameter	-10%		+10%	
	T_{new}	DT%	T_{new}	DT%
V5max	23.57	-0.342	23.72	0.288
V6max	23.67	0.085	23.64	-0.051
V7max	23.65	0.004	23.65	-0.004
V8max	23.63	-0.068	23.67	0.063
V9max	23.65	0	23.65	0
V10max	23.64	-0.025	23.65	0.017
kt1	23.68	0.118	23.83	0.77
ki1	23.66	0.042	23.69	0.182
kt2	23.6	-0.199	23.69	0.186
ki2	23.77	0.486	23.61	-0.161
ki21	23.67	0.063	23.64	-0.051
kt3	24.01	1.522	23.55	-0.44
ki3	23.64	-0.034	23.66	0.03
kt4	23.64	-0.047	23.67	0.072
ki4	23.47	-0.753	23.69	0.165
kt5	23.68	0.14	23.61	-0.178
ki5	23.81	0.681	23.51	-0.6
kii1	23.65	0.004	23.65	-0.004
kt5_e	23.67	0.076	23.63	-0.072
kt6	23.64	-0.034	23.66	0.042
kt7	23.65	0	23.65	0
ki8	23.65	-0.004	23.65	0.004
kii8	23.65	0.004	23.65	-0.004
kt9	23.65	0	23.65	0
ki9	23.65	0	23.65	0
kt10	23.65	0.017	23.65	-0.017
kt11	23.66	0.03	23.65	-0.021
kt12	23.66	0.042	23.64	-0.038
a	23.67	0.101	23.63	-0.08
d	23.68	0.135	23.63	-0.097
g	23.32	-1.404	23.95	1.285
h	23.61	-0.186	23.68	0.118
i	23.59	-0.245	23.7	0.211
a_1	23.59	-0.266	23.71	0.233
o	23.67	0.076	23.64	-0.055
l	23.65	0	23.65	-0.004
l1	23.65	0	23.65	0

Parameter	-10%		+10%	
	T_{new}	DT%	T_{new}	DT%
r1	23.65	-0.013	23.65	0.008
r2	23.64	-0.047	23.67	0.08
r3	23.64	-0.055	23.66	0.051
kp1	23.7	0.224	23.6	-0.199
kp2	23.67	0.093	23.64	-0.063
kp3	23.48	-0.727	23.81	0.672
kp4	23.6	-0.199	23.68	0.131
kp5	23.57	-0.342	23.72	0.288
kp6	23.67	0.085	23.64	-0.051
kp7	23.65	0.004	23.65	-0.004
kp8	23.63	-0.068	23.67	0.063
kp10	23.64	-0.025	23.65	0.017
kp11	23.64	-0.047	23.67	0.085
kp12	23.63	-0.097	23.67	0.08
kiz4	23.68	0.11	23.63	-0.106
kiz5	23.69	0.182	23.61	-0.161
kiz6	23.78	0.562	23.55	-0.423
kiz7	23.64	-0.051	23.65	0.008
kiz8	23.62	-0.144	23.67	0.085
kex2	23.68	0.14	23.62	-0.127
kex3	23.65	0.008	23.65	-0.008
kiz10	23.65	-0.017	23.65	0.013
kiz11	23.66	0.059	23.64	-0.042
kiz9	23.65	0.004	23.65	-0.004
kiz12	23.64	-0.047	23.67	0.068
kiz13	23.63	-0.076	23.67	0.063
kiz14	23.64	-0.051	23.66	0.047
b	23.65	-0.013	23.79	0.575
c	24	1.476	23.47	-0.753
e	23.63	-0.106	23.68	0.114
f	23.65	-0.021	23.67	0.063
f1	23.65	-0.017	23.65	0.013
v	23.41	-1.006	23.83	0.748
w	23.57	-0.33	23.71	0.254
p	23.66	0.03	23.64	-0.025
q	23.74	0.381	23.34	-1.332
n	23.75	0.44	23.56	-0.389

Parameter	-10%		+10%	
	T_{new}	DT%	T_{new}	DT%
m	23.19	-1.928	24.05	1.674
r	23.65	-0.004	23.65	0.017
s	23.65	-0.004	23.65	0.004
h4	23.65	-0.017	23.65	0.017
h5	23.65	-0.008	23.65	0.004
h6	23.65	0	23.65	0
h7	23.65	0	23.65	0
h1	23.66	0.021	23.65	-0.021
h8	23.65	0.008	23.65	-0.008
a_2	23.65	-0.017	23.65	0.017
h2	23.65	-0.017	23.65	0.017
h3	23.67	0.08	23.63	-0.085
source_p53	23.65	0.017	23.65	-0.017
source_Rb	23.65	0.008	23.65	-0.008

Table 5: Effect of gene knock-outs on RNA circadian period – comparison of *in silico* with experimental data. WT, wild type; +, period increase; -, period increase; AR, arrhythmic phenotype; - then AR, decrease in the period followed by arrhythmic phenotype; + then AR, increase in the period followed by arrhythmic phenotype; nd, not defined.

**Gene**	**Mutation phenotype animal model – mouse**	***in silico* data mutants** transcription rate (-90%)	knock-out
Bmal1	AR [42, 43]	AR	AR
Bmal2	nd		
Per1	- then AR [42, 43]	+ then AR	AR
Per2	- then AR [42, 43]		
Per3	- [42, 43]		
Per1+Per3	- then AR [43]		
Per2+Per3	- then AR [43]		
Per1+Per2	AR [43]		
Cry1	- [42, 43]	AR	+
Cry2	+ [42, 43]		
Cry1+Cry2	AR [43]		
Rev-erbα	- [42, 43]	AR	AR
Rev-erbβ	nd		
Rorα	- [44]	AR	AR
Rorβ	+ [45]		
Rorγ	nd		
Ink4a	WT	+	+
Arf	nd	WT	WT
Myc	nd	-	-
Mdm2	nd	-	+
CDK/CycD	nd	WT	WT
E2f	nd	-	-
p53	nd	nd	+

References

1. Relógio, A., et al., *Tuning the mammalian circadian clock: robust synergy of two loops.* PLoS Comput Biol, 2011. **7**(12): p. e1002309.
2. Fu, L., et al., *The circadian gene Period2 plays an important role in tumor suppression and DNA damage response in vivo.* Cell, 2002. **111**(1): p. 41-50.
3. Matsuo, T., et al., *Control mechanism of the circadian clock for timing of cell division in vivo.* Science, 2003. **302**(5643): p. 255-9.
4. Kowalska, E., et al., *NONO couples the circadian clock to the cell cycle.* Proc Natl Acad Sci U S A, 2013. **110**(5): p. 1592-9.
5. McConnell, B.B., et al., *Induced expression of p16 INK4a inhibits both CDK4-and CDK2-associated kinase activity by reassortment of cyclin-CDK-inhibitor complexes.* Molecular and cellular biology, 1999. **19**(3): p. 1981-1989.
6. Zindy, F., et al., *Myc signaling via the ARF tumor suppressor regulates p53-dependent apoptosis and immortalization.* Genes Dev, 1998. **12**(15): p. 2424-33.
7. Wong, J.V., et al., *Viral-mediated noisy gene expression reveals biphasic E2f1 response to MYC.* Mol Cell, 2011. **41**(3): p. 275-85.
8. Weber, J.D., et al., *Nucleolar Arf sequesters Mdm2 and activates p53.* Nat Cell Biol, 1999. **1**(1): p. 20-6.
9. Hermeking, H., et al., *Identification of CDK4 as a target of c-MYC.* Proc Natl Acad Sci U S A, 2000. **97**(5): p. 2229-34.
10. Nobori, T., et al., *Deletions of the cyclin-dependent kinase-4 inhibitor gene in multiple human cancers.* Nature, 1994. **368**(6473): p. 753-6.
11. Grandinetti, K.B. and G. David, *Sin3B: an essential regulator of chromatin modifications at E2F target promoters during cell cycle withdrawal.* Cell Cycle, 2008. **7**(11): p. 1550-4.
12. Rayman, J.B., et al., *E2F mediates cell cycle-dependent transcriptional repression in vivo by recruitment of an HDAC1/mSin3B corepressor complex.* Genes Dev, 2002. **16**(8): p. 933-47.
13. Xie, X., P. Rigor, and P. Baldi, *MotifMap: a human genome-wide map of candidate regulatory motif sites.* Bioinformatics, 2009. **25**(2): p. 167-74.
14. Sears, R., et al., *Ras enhances Myc protein stability.* Mol Cell, 1999. **3**(2): p. 169-79.
15. Leone, G., et al., *Myc requires distinct E2F activities to induce S phase and apoptosis.* Mol Cell, 2001. **8**(1): p. 105-13.
16. Altman, B.J., et al., *MYC disrupts the circadian clock and metabolism in cancer cells.* Cell metabolism, 2015. **22**(6): p. 1009-1019.
17. Shostak, A., et al., *MYC/MIZ1-dependent gene repression inversely coordinates the circadian clock with cell cycle and proliferation.* Nature communications, 2016. **7**.
18. Repouskou, A. and A. Prombona, *c-MYC targets the central oscillator gene Per1 and is regulated by the circadian clock at the post-transcriptional level.* Biochimica et Biophysica Acta (BBA)-Gene Regulatory Mechanisms, 2016. **1859**(4): p. 541-552.
19. Sherr, C.J. and J.M. Roberts, *CDK inhibitors: positive and negative regulators of G1-phase progression.* Genes & development, 1999. **13**(12): p. 1501-1512.
20. Harbour, J.W. and D.C. Dean, *The Rb/E2F pathway: expanding roles and emerging paradigms.* Genes & development, 2000. **14**(19): p. 2393-2409.
21. Polager, S. and D. Ginsberg, *p53 and E2f: partners in life and death.* Nature Reviews Cancer, 2009.

9(10): p. 738-748.
22. Zhang, Y., Y. Xiong, and W.G. Yarbrough, *ARF promotes MDM2 degradation and stabilizes p53: ARF-INK4a locus deletion impairs both the Rb and p53 tumor suppression pathways.* Cell, 1998. **92**(6): p. 725-34.
23. Miki, T., et al., *p53 regulates Period2 expression and the circadian clock.* Nature communications, 2013. **4**.
24. Kuo, M.L., et al., *N-terminal polyubiquitination and degradation of the Arf tumor suppressor.* Genes Dev, 2004. **18**(15): p. 1862-74.
25. Finlay, C.A., *The mdm-2 oncogene can overcome wild-type p53 suppression of transformed cell growth.* Mol Cell Biol, 1993. **13**(1): p. 301-6.
26. Chen, X., et al., *Ubiquitin-independent degradation of cell-cycle inhibitors by the REGgamma proteasome.* Mol Cell, 2007. **26**(6): p. 843-52.
27. Bates, S., et al., *Absence of cyclin D/cdk complexes in cells lacking functional retinoblastoma protein.* Oncogene, 1994. **9**(6): p. 1633-40.
28. Diehl, J.A., F. Zindy, and C.J. Sherr, *Inhibition of cyclin D1 phosphorylation on threonine-286 prevents its rapid degradation via the ubiquitin-proteasome pathway.* Genes Dev, 1997. **11**(8): p. 957-72.
29. Helin, K., *Regulation of cell proliferation by the E2F transcription factors.* Curr Opin Genet Dev, 1998. **8**(1): p. 28-35.
30. Mihara, K., et al., *Cell cycle-dependent regulation of phosphorylation of the human retinoblastoma gene product.* Science, 1989. **246**(4935): p. 1300-3.
31. Buchler, N.E., U. Gerland, and T. Hwa, *Nonlinear protein degradation and the function of genetic circuits.* Proc Natl Acad Sci U S A, 2005. **102**(27): p. 9559-64.
32. Yao, G., et al., *A bistable Rb-E2F switch underlies the restriction point.* Nat Cell Biol, 2008. **10**(4): p. 476-82.
33. von Willebrand, M., et al., *The tyrphostin AG1024 accelerates the degradation of phosphorylated forms of retinoblastoma protein (pRb) and restores pRb tumor suppressive function in melanoma cells.* Cancer Res, 2003. **63**(6): p. 1420-9.
34. Ramsay, G., G.I. Evan, and J.M. Bishop, *The protein encoded by the human proto-oncogene c-myc.* Proc Natl Acad Sci U S A, 1984. **81**(24): p. 7742-6.
35. Gregory, M.A. and S.R. Hann, *c-Myc proteolysis by the ubiquitin-proteasome pathway: stabilization of c-Myc in Burkitt's lymphoma cells.* Mol Cell Biol, 2000. **20**(7): p. 2423-35.
36. Relogio, A., et al., *Tuning the mammalian circadian clock: robust synergy of two loops.* PLoS Comput Biol, 2011. **7**(12): p. e1002309.
37. Mendrysa, S.M., M.K. McElwee, and M.E. Perry, *Characterization of the 5' and 3' untranslated regions in murine mdm2 mRNAs.* Gene, 2001. **264**(1): p. 139-46.
38. Grafstrom, R.H., W. Pan, and R.H. Hoess, *Defining the substrate specificity of cdk4 kinase-cyclin D1 complex.* Carcinogenesis, 1999. **20**(2): p. 193-8.
39. Kholodenko, B.N., *Cell-signalling dynamics in time and space.* Nat Rev Mol Cell Biol, 2006. **7**(3): p. 165-76.
40. Leenders, G.B. and J.A. Tuszynski, *Stochastic and Deterministic Models of Cellular p53 Regulation.* Front Oncol, 2013. **3**: p. 64.
41. Weinberg, R.L., et al., *Comparative binding of p53 to its promoter and DNA recognition elements.* J Mol Biol, 2005. **348**(3): p. 589-96.

42. Lowrey, P.L. and J.S. Takahashi, *Mammalian circadian biology: elucidating genome-wide levels of temporal organization.* Annual review of genomics and human genetics, 2004. **5**: p. 407.

43. Reppert, S.M. and D.R. Weaver, *Coordination of circadian timing in mammals.* Nature, 2002. **418**(6901): p. 935-941.

44. Sato, T.K., et al., *A functional genomics strategy reveals Rora as a component of the mammalian circadian clock.* Neuron, 2004. **43**(4): p. 527-537.

45. André, E., et al., *Disruption of retinoid - related orphan receptor β changes circadian behavior, causes retinal degeneration and leads to vacillans phenotype in mice.* The EMBO Journal, 1998. **17**(14): p. 3867-3877.
