# Supplementary material for: The Ink4a/Arf locus operates as a regulator of the circadian clock modulating RAS activity
Source: PLoS Biol. 2017 Dec 7;15(12):e2002940. doi: 10.1371/journal.pbio.2002940 (PMC5720494; doi:10.1371/journal.pbio.2002940)
Supplement: S2 Text — Microarray data were subjected to standard statistical tests to assess their quality. (PDF) [file pbio.2002940.s010.pdf]

Supporting Information S2 Text - Quality control of microarrays

The following quality control procedure was carried out in all microarrays the Bioconductor package arrayQualityMetrics [1]. A total of five quality tests were performed (**Figures 1 – 12**). Since the arrays passed all tests, they were all used for the subsequent analysis (**Table 1**). The outlier detection criteria are explained below in the respective sections.

Sample Name	Condition	Outlier detection by distances between arrays	Principal component analysis	Outlier detection by boxplots
1	Ink4a/Arf$^{-/-}$	passed	passed	passed
2	Ink4a/Arf$^{-/-}$+RAS	passed	passed	passed
3	Ink4a/Arf$^{-/-}$ shBmal1	passed	passed	passed
4	Ink4a/Arf$^{-/-}$ shBmal1+RAS	passed	passed	passed
5	Ink4a/Arf$^{+/+}$	passed	passed	passed
6	Ink4a/Arf$^{+/+}$+RAS	passed	passed	passed
7	Ink4a/Arf$^{+/+}$ shBmal1	passed	passed	passed
8	Ink4a/Arf$^{+/+}$ shBmal1+RAS	passed	passed	passed

Table 1. Array metadata and outlier detection overview.

1 – Outlier detection by distance between arrays

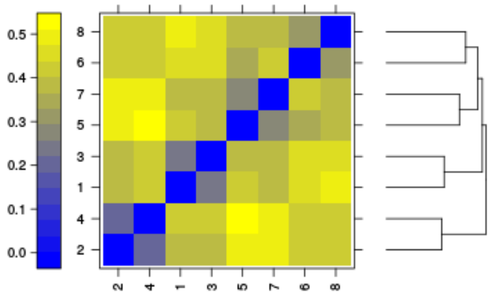

Figure 1. False colour heatmap of the distances between arrays. The colour scale is chosen to cover the range of distances encountered in the dataset. Patterns in this plot can indicate clustering of the arrays either because of intended biological or unintended experimental factors (batch effects). The distance d_{ab} between two arrays a and b is computed as the mean absolute difference (L_1-distance) between the data of the arrays (using the data from all probes without filtering). In formula, d_{ab} = mean | M_{ai} - M_{bi} |, where M_{ai} is the value of the i-th probe on the a-th array. Outlier detection was performed by looking for arrays for which the sum of the distances to all other arrays, $S_a = \Sigma_b d_{ab}$ was exceptionally large. No such arrays were detected.

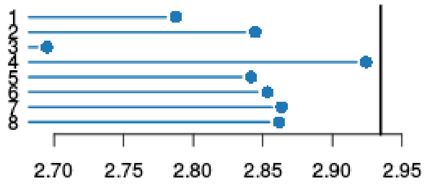

Figure 2. Bar chart of the sum of distances to other arrays S_a, the outlier detection criterion from **Figure 1**. Based on the distribution of the values across all arrays, a threshold of 2.93 was determined, which is indicated by the vertical line. None of the arrays exceeded the threshold.

2 – Principal Component Analysis

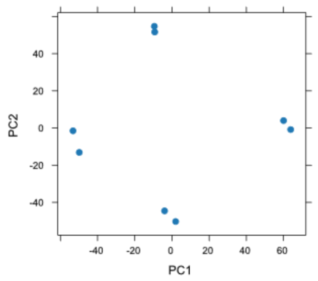

Figure 3. Scatterplot of the arrays along the first two principal components. This plot can be used to explore if the arrays cluster, and whether this is according to an intended experimental factor or according to unintended causes such as batch effects. Principal component analysis is a dimension reduction and visualisation technique that is here used to project the multivariate data vector of each array into a two-dimensional plot, such that the spatial arrangement of the points in the plot reflects the overall data (dis)similarity between the arrays.

3 - Outlier detection by Boxplots

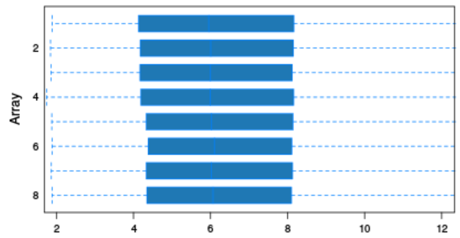

Figure 4. Boxplot representing summaries of the signal intensity distributions of the arrays. Each box corresponds to one array. Typically, it is expected that the boxes have similar positions and widths. If the distribution of an array is very different from the others, this may indicate an experimental problem. Outlier detection was performed by computing the Kolmogorov-Smirnov statistic K_a between each array's distribution and the distribution of the pooled data.

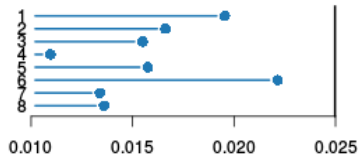

Figure 5. Bar chart of the Kolmogorov-Smirnov statistic K_a, the outlier detection criterion from Figure 4. Based on the distribution of the values across all arrays, a threshold of 0.0.25 was determined, which is indicated by the vertical line. None of the arrays exceeded the threshold.

4 – Density Plot

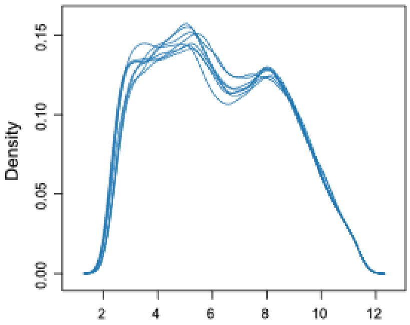

Figure 6. Density estimates (smoothed histograms) of the data. Typically, the distributions of the arrays should have similar shapes and ranges. Arrays whose distributions are very different from the others should be considered for possible problems. Various features of the distributions can be indicative of quality related phenomena. For instance, high levels of background will shift an array's distribution to the right. Lack of signal diminishes its right tail. A bulge at the upper end of the intensity range often indicates signal saturation.

5 – Standard deviation versus rank of the mean

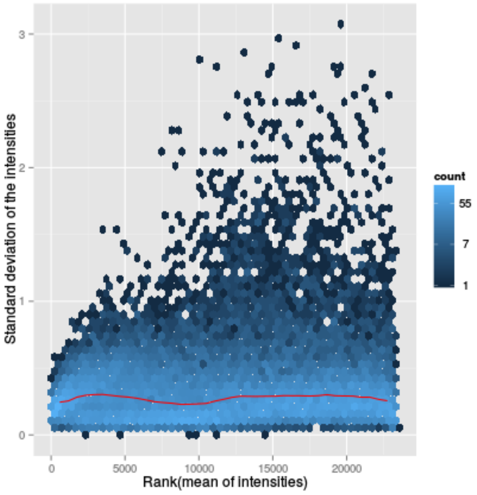

Figure 7. Density plot of the standard deviation of the intensities across arrays on the *y*-axis versus the rank of their mean on the *x*-axis. The red dots, connected by lines, show the running median of the standard deviation. After normalisation and transformation to a logarithm(-like) scale, one typically expects the red line to be approximately horizontal and to show no substantial trend. In some cases, a hump on the right hand of the x-axis can be observed and is symptomatic of a saturation of the intensities.

6 - Outlier detection by MA plots

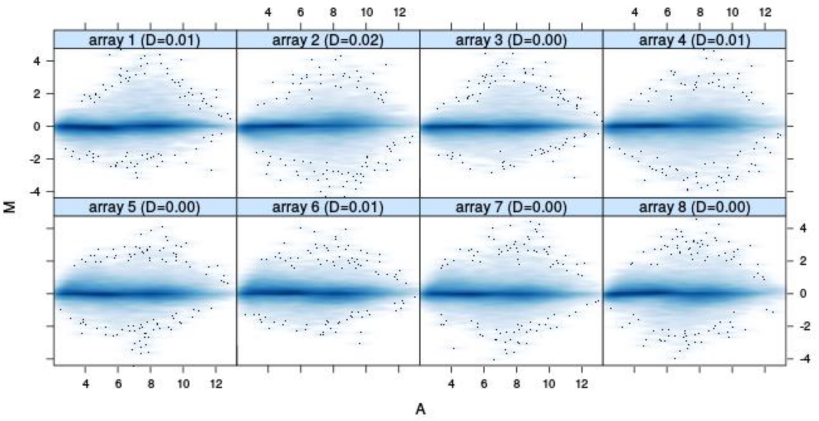

Figure 8. MA plots. M and A are defined as: $M = \log_2(I_1) - \log_2(I_2)$ $A = 1/2\ (\log_2(I_1)+\log_2(I_2))$, where I_1 is the intensity of the array studied, and I_2 is the intensity of a "pseudo"-array that consists of the median across arrays. Typically, we expect the mass of the distribution in an MA plot to be concentrated along the M = 0 axis, and there should be no trend in M as a function of A. If there is a trend in the lower range of A, this often indicates that the arrays have different background intensities; this may be addressed by background correction. A trend in the upper range of A can indicate saturation of the measurements; in mild cases, this may be addressed by non-linear normalisation (e.g. quantile normalisation). Outlier detection was performed by computing Hoeffding's statistic D_a on the joint distribution of A and M for each array. Shown are the 4 arrays with the highest value of D_a (top row), and the 4 arrays with the lowest value (bottom row). The value of D_a is shown in the panel headings. 6 arrays had D_a>0.15 and were marked as outliers.

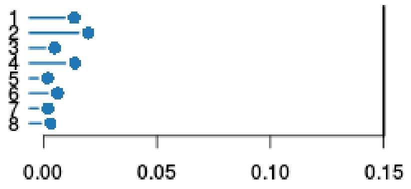

Figure 9. Bar chart of the Hoeffding's statistic D_a, the outlier detection criterion from the previous figure. The bars are shown in the original order of the arrays. A threshold of 0.15 was used, which is indicated by the vertical line. 6 arrays exceeded the threshold.

References

1. Kauffmann A, Gentleman R, Huber W (2009) arrayQualityMetrics--a bioconductor package for quality assessment of microarray data. Bioinformatics 25: 415-416.
