# Supplementary material for: The Ink4a/Arf locus operates as a regulator of the circadian clock modulating RAS activity
Source: PLoS Biol. 2017 Dec 7;15(12):e2002940. doi: 10.1371/journal.pbio.2002940 (PMC5720494; doi:10.1371/journal.pbio.2002940)
Supplement: S3 Text — (PDF) [file pbio.2002940.s011.pdf]

Supporting Information S3 Text – Potential link between Clock/Bmal and E2f

To investigate the coupling between the circadian clock and the cell cycle *in silico*, we have assembled a mathematical model containing the core-clock and connecting elements to the cell cycle that allow for the inclusion of the genes *Ink4a* and *Arf*, corresponding to our experimental model. Several bridging points between both oscillators have been reported in the literature, these include Myc, Wee1, p53, Ink4a, Arf (via Myc) [1-5]. Myc is known to downregulate CLOCK/BMAL-mediated transcription via competitive targeting of E-box sequences [6]. Myc also activates E2F [7, 8] which was found to act as an important binding element between the clock and the cell cycle in eukaryotes. For example, in the unicellular red alga *Cyanidioschyzon merolae* where time-dependent phosphorylation of E2F promotes the G1/S transition, and a mutation of the E2F phosphorylation sites results in a uncoupling of cell cycle progression from the circadian clock [9]. Furthermore, a bioinformatics analysis by the MotifMap database (that uses databases of transcription factor binding motifs, refined genome alignments, and a comparative genomic statistical approach to identify candidate regulatory motif sites [10]) has identified *Bmal1* as a potential E2F target gene in humans. E2F is a known regulator of G1/S transition of the cell cycle. Instead of transitioning to the S-phase, cells can also go into senescence as an alternative cell cycle fate decision, a process that is known to be induced by RAS (oncogene induced senescence, OIS) in MEFs. Moreover, upon RAS induction in WT MEFs, we observe a change in the expression levels of *Bmal1*. This is further supported by our ChIP-Seq analysis of the H3K9me decoration of the *Bmal1* promoter regions which shows that *Bmal1* transcript expression is enhanced when RAS is overexpressed in normal fibroblasts (**Figure 1**). Altogether, this data points to a connection between E2F and the clock which – given the existing data – can be postulated to happen via activation of *Bmal1*.

Interestingly, if we increase the strength of the E2F activation on *Bmal* in the model (see control coefficient analysis, **Text S1 Table 4**) we obtain a longer period, which is consistent with the increase of *Bmal1* in the WT MEFs after RAS induction and the longer period phenotype observed. This connection may be more complex than we hypothesise in our current model, and should be seen as one of the predictions generated by our model that might be experimentally tested in future work.

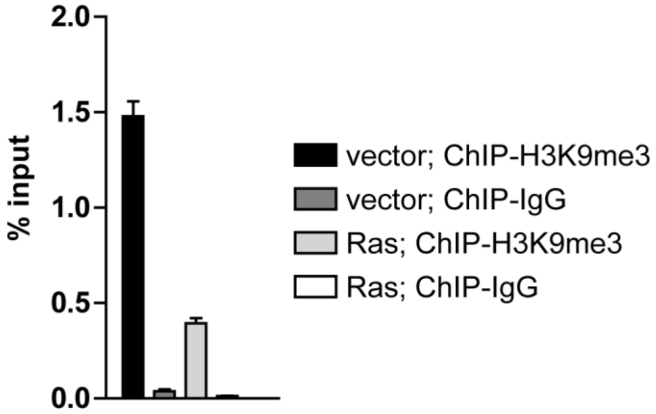

Figure 1: ChIP assays of human diploid fibroblasts (HDFs) IMR-90 transduced with oncogenic RAS or empty vector as control, using an anti-H3K9me3 antibody and PCR primer specific for the *Bmal1* promoter. Input DNA (no immunoprecipitation) was used as internal control and anti-IgG was used as a negative control. Numerical values are provided in S1 Data.

References

1. Fu L, Kettner NM. The circadian clock in cancer development and therapy. Progress in molecular biology and translational science. 2013;119:221.
2. Sahar S, Sassone-Corsi P. Metabolism and cancer: the circadian clock connection. Nature Reviews Cancer. 2009;9(12):886-96.
3. Kelleher FC, Rao A, Maguire A. Circadian molecular clocks and cancer. Cancer letters. 2014;342(1):9-18.
4. Fu L, Lee CC. The circadian clock: pacemaker and tumour suppressor. Nature reviews Cancer. 2003;3(5):350.
5. Kowalska E, Ripperger JA, Hoegger DC, Bruegger P, Buch T, Birchler T, et al. NONO couples the circadian clock to the cell cycle. Proceedings of the National Academy of Sciences. 2013;110(5):1592-9.
6. Repouskou A, Prombona A. c-MYC targets the central oscillator gene Per1 and is regulated by the circadian clock at the post-transcriptional level. Biochimica et Biophysica Acta (BBA)-Gene Regulatory Mechanisms. 2016;1859(4):541-52.
7. Leone G, Sears R, Huang E, Rempel R, Nuckolls F, Park C-H, et al. Myc requires distinct E2F activities to induce S phase and apoptosis. Molecular cell. 2001;8(1):105-13.
8. Sears R, Leone G, DeGregori J, Nevins JR. Ras enhances Myc protein stability. Molecular cell. 1999;3(2):169-79.
9. Miyagishima S-y, Fujiwara T, Sumiya N, Hirooka S, Nakano A, Kabeya Y, et al. Translation-independent circadian control of the cell cycle in a unicellular photosynthetic eukaryote. Nature communications. 2014;5:3807.
10. Daily K, Patel VR, Rigor P, Xie X, Baldi P. MotifMap: integrative genome-wide maps of regulatory motif sites for model species. BMC bioinformatics. 2011;12(1):495.
