# Supplementary material for: The Ink4a/Arf locus operates as a regulator of the circadian clock modulating RAS activity
Source: PLoS Biol. 2017 Dec 7;15(12):e2002940. doi: 10.1371/journal.pbio.2002940 (PMC5720494; doi:10.1371/journal.pbio.2002940)

Supporting Information S4 Text – Gating strategies for the FACS analysis

The cells of interest were gated based on forward scatter/side scatter (FSC vs. SSC) values. The cell cycle analysis was conducted by fitting a univariate cell cycle model to the previously gated population using the Watson pragmatic algorithm as implemented in FlowJo v10.2 (FlowJo LLC).

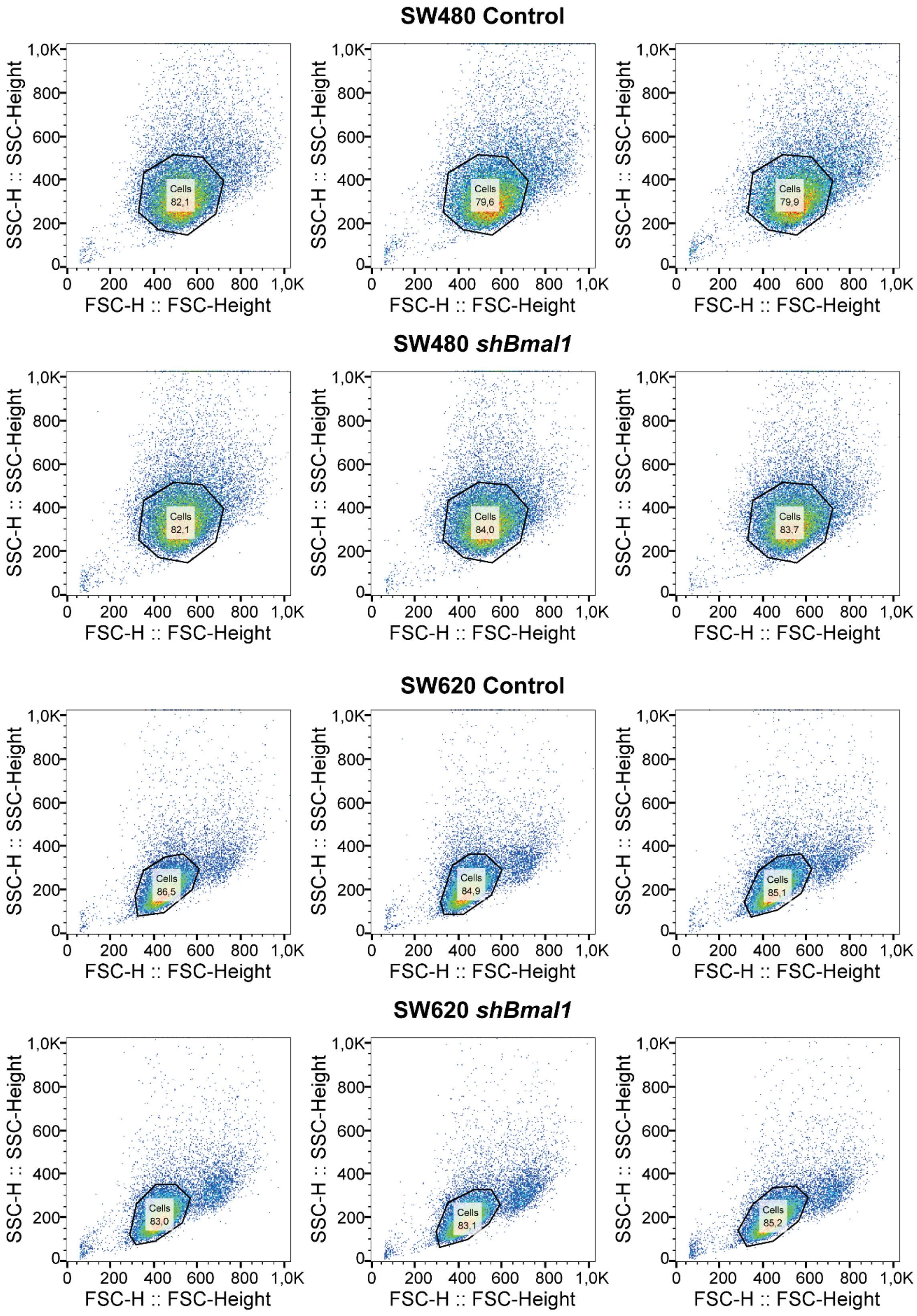

Ink4a/Arf$^{+/+}$ MEFs

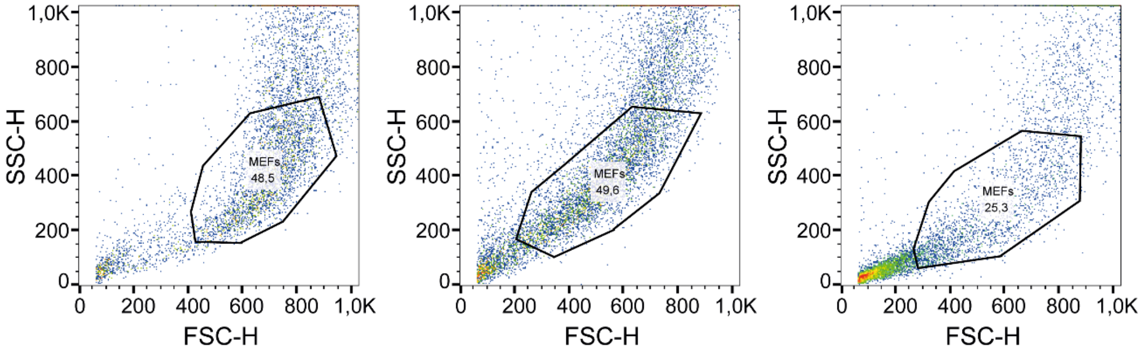

Ink4a/Arf$^{+/+}$+RAS MEFs

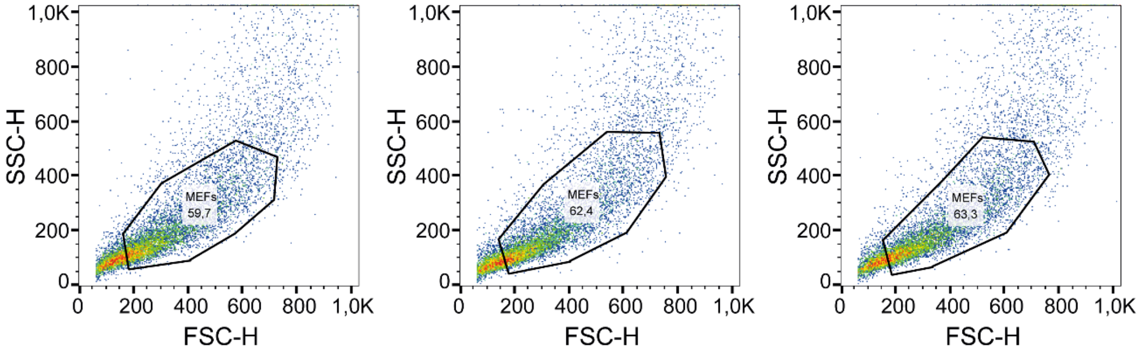

Ink4a/Arf$^{-/-}$ MEFs

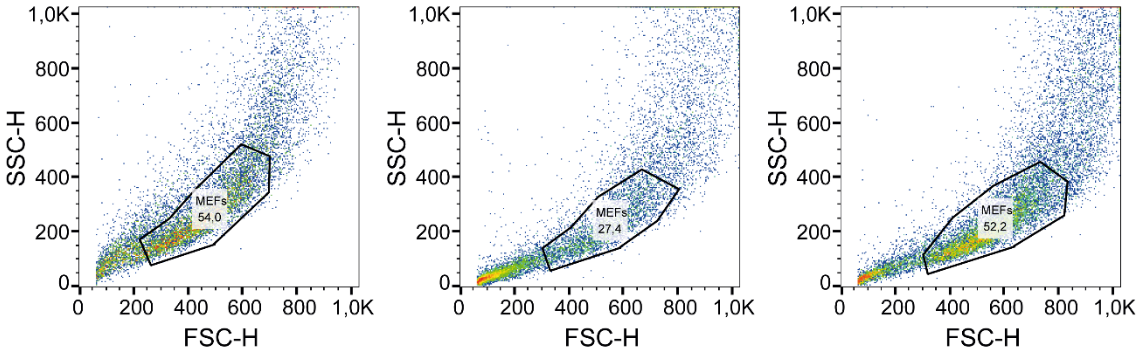

Ink4a/Arf$^{-/-}$+RAS MEFs

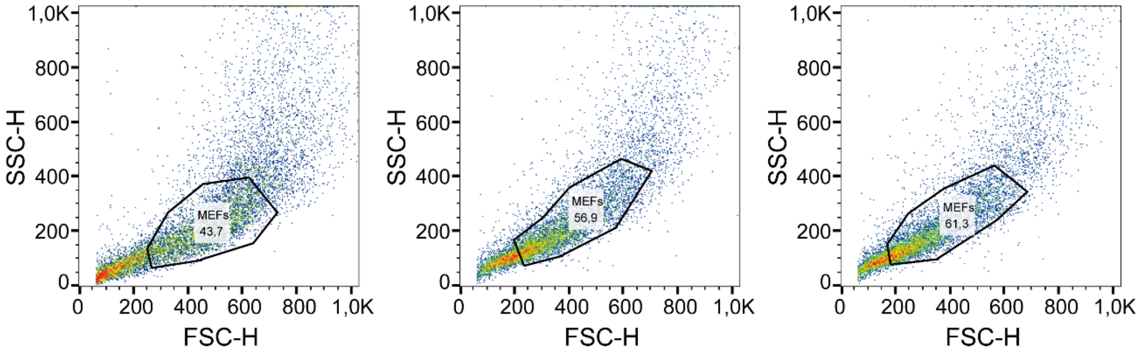

Ink4a/Arf$^{-/-}$ *shBmal1*+RAS

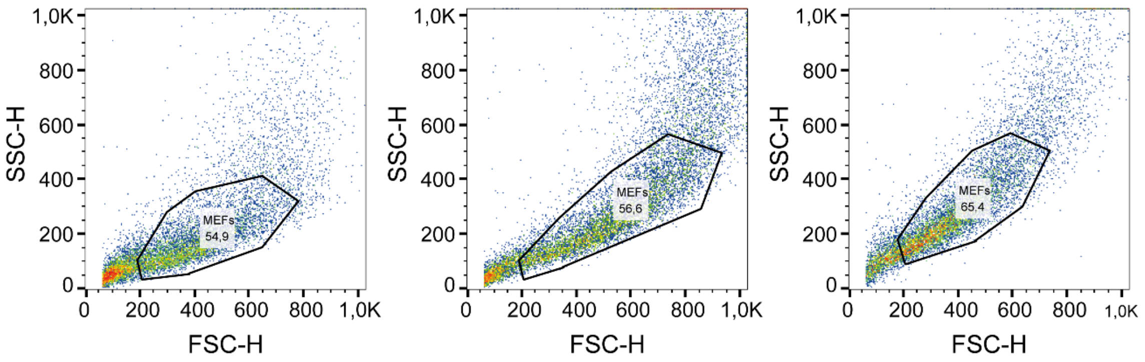

Supplement: S4 Text — Description of the gating strategies applied for the cell cycle analysis of the MEF cells and the SW480 and SW620 cells. (PDF) [file pbio.2002940.s012.pdf]
